# Supplementary material for: Design, Synthesis and Bioactive Evaluation of Topo I/c-MYC Dual Inhibitors to Inhibit Oral Cancer via Regulating the PI3K/AKT/NF-κB Signaling Pathway
Source: Molecules. 2025 Feb 14;30(4):894. doi: 10.3390/molecules30040894 (PMC11858653; doi:10.3390/molecules30040894)
Supplement: Supplementary file 1 [file molecules-30-00894-s001.zip › molecules-3417859-supplementary.pdf]

Supporting Information

Design, Synthesis and Bioactive Evaluation of Topo I/*c-MYC* Dual Inhibitors to Inhibit Oral Cancer via Regulating the PI3K/AKT/NF-κB Signaling Pathway

Bin Zheng, Yi-Xiao Wang, Zi-Yan Wu, Xin-Wei Li, Li-Qing Qin, Nan-Ying Chen, Gui-Fa Su, Jun-Cheng Su and Cheng-Xue Pan*

State Key Laboratory for Chemistry and Molecular Engineering of Medicinal Resources, Key Laboratory for Chemistry and Molecular Engineering of Medicinal Resources (Ministry of Education of China), Collaborative Innovation Center for Guangxi Ethnic Medicine, School of Chemistry and Pharmaceutical Sciences, Guangxi Normal University, 15 Yu Cai Road, Guilin, 541004, China

*Correspondence: chengxuepan@163.com; Tel./Fax: +86-773-2535678

1. General for the synthesis and characterization

The major reagents employed in this work are commercially available and do not require further purification unless otherwise noted. The NMR spectra were recorded on Bruker Advance (400 MHz and 600 MHz), with $CDCl_3$ or DMSO-d_6 as a solvent. Chemical shifts were recorded in δ values. HRESIMS data were acquired on a Thermo-Scientific Exactive mass spectrometer.

2. X-ray crystallography of compounds **7ag**, **7ai** and **7be**

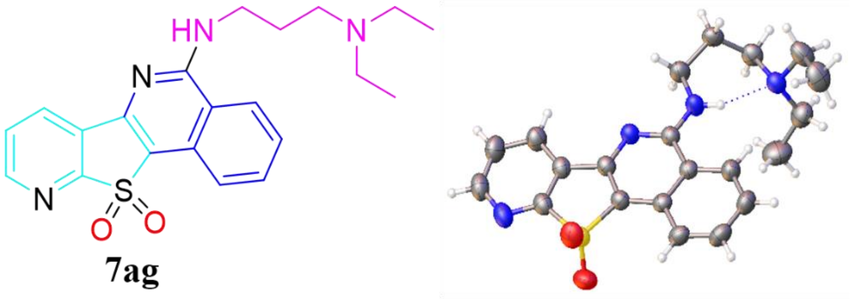

Figure S1. ORTEP diagram of **7ag** at 50% ellipsoid probability

CCDC No: 2341050, the detailed crystallographic data can be obtained free of charge from The Cambridge Crystallographic Data Centre.

Preparation of **7ag** crystal. **7ag** (30 mg) was dissolved in toluene (5 ml) at room temperature and left to precipitate monoclinic crystals after 1-3 days.

Table S1. Crystal data and structure refinement for **7ag**

Compound	**7ag**
Empirical formula	$C_{21}H_{24}N_4O_2S$
Formula weight	396.50
Temperature/K	293
Crystal system	monoclinic
Space group	$P2_1/c$
a/Å	13.0482 (2)
b/Å	7.5185 (1)
c/Å	20.1642 (3)
α/°	90
β/°	92.986 (1)
γ/°	90
Volume/Å^3	1975.48 (5)
Z	4
ρ_{calc}g/cm^3	1.333
μ/mm^{-1}	1.655
F(000)	840
Crystal size/mm^3	-
Radiation	Cu Kα (λ = 1.54184)
2Θ range for data collection/°	6.784 to 151.242
Index ranges	$-16 \le h \le 15$, $-9 \le k \le 4$, $-25 \le l \le 25$
Reflections collected	14157
Independent reflections	3926 [R_{int} = 0.0209, R_{sigma} = 0.0193]
Data/restraints/parameters	3926/0/256
Goodness-of-fit on F^2	1.087
Final R indexes [I>=2σ (I)]	R_1 = 0.0357, wR_2 = 0.1043
Final R indexes [all data]	R_1 = 0.0383, wR_2 = 0.1068
Largest diff. peak/hole / e Å^{-3}	0.30/-0.28

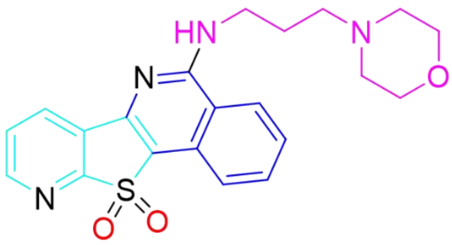

7ai

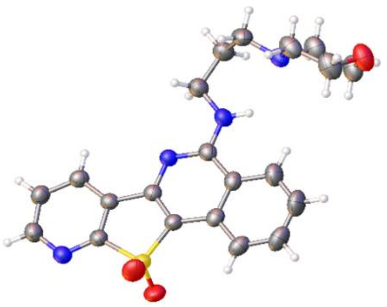

Figure S2. ORTEP diagram of **7ai** at 50% ellipsoid probability

CCDC No: 2405703, the detailed crystallographic data can be obtained free of charge from The Cambridge Crystallographic Data Centre.

Preparation of **7ai** crystal. **7ai** (30 mg) was dissolved in toluene (5 ml) at room temperature and left to precipitate monoclinic crystals after 1-3 days.

Table S2. Crystal data and structure refinement for **7ai**

Compound	**7ai**
Empirical formula	$C_{21}H_{22}N_4O_3S$
Formula weight	410.48
Temperature/K	298
Crystal system	monoclinic
Space group	$P2_1/n$
a/Å	12.3082 (2)
b/Å	10.5428 (1)
c/Å	16.2830 (2)
α/°	90
β/°	111.573 (2)
γ/°	90
Volume/Å^3	1964.92 (5)
Z	4
ρ_{calc}g/cm^3	1.388
μ/mm^{-1}	1.73
F(000)	864
Crystal size/mm^3	0.2 × 0.1 × 0.1
Radiation	Cu Kα (λ = 1.54184)
2Θ range for data collection/°	7.784 to 154.242
Index ranges	$-15 \leq h \leq 14$, $-13 \leq k \leq 12$, $-14 \leq l \leq 20$
Reflections collected	17639
Independent reflections	3966 [R_{int} = 0.043, R_{sigma} = 0.0262]
Data/restraints/parameters	3966/0/263
Goodness-of-fit on F^2	1.061
Final R indexes [I>=2σ (I)]	R_1 = 0.0426, wR_2 = 0.1204
Final R indexes [all data]	R_1 = 0.0465, wR_2 = 0.1233
Largest diff. peak/hole / e Å^{-3}	0.26/-0.31

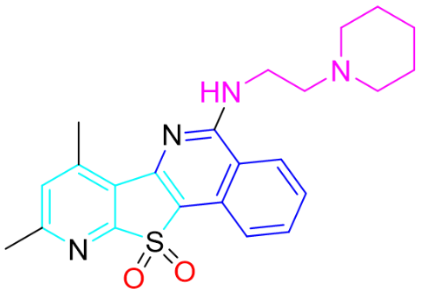

7be

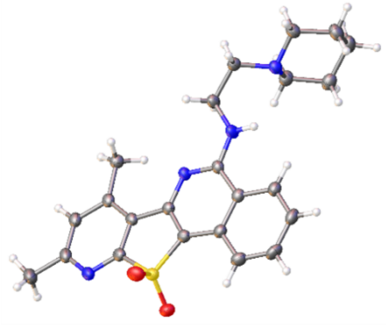

Figure S3. ORTEP diagram of **7be** at 50% ellipsoid probability

CCDC No: 2341051, the detailed crystallographic data can be obtained free of charge from The Cambridge Crystallographic Data Centre.

Preparation of **7be** crystal. **7be** (30 mg) was dissolved in toluene (5 ml) at room temperature and left to precipitate monoclinic crystals after 1-3 days.

Table S3. Crystal data and structure refinement for **7be**

Compound	**7be**
Empirical formula	$C_{46}H_{52}N_8O_4S_2$
Formula weight	845.07
Temperature/K	100
Crystal system	monoclinic
Space group	$P2_1/n$
a/Å	17.6237 (1)
b/Å	13.2073 (1)
c/Å	17.6569 (2)
α/°	90
β/°	91.413 (1)
γ/°	90
Volume/Å^3	4108.60(6)
Z	4
ρ_{calc}g/cm^3	1.366
μ/mm^{-1}	1.63
F(000)	1792
Crystal size/mm^3	0.3 × 0.2 × 0.1
Radiation	Cu Kα (λ = 1.54184)
2Θ range for data collection/°	7 to 154.492
Index ranges	-20 ≤ h ≤ 22, -16 ≤ k ≤ 16, -21 ≤ l ≤ 22
Reflections collected	58281
Independent reflections	8522 [R_{int} = 0.0834, R_{sigma} = 0.0423]

Data/restraints/parameters	8522/0/545
Goodness-of-fit on F^2	1.079
Final R indexes [I>=2σ (I)]	R_1 = 0.0467, wR_2 = 0.1286
Final R indexes [all data]	R_1 = 0.0545, wR_2 = 0.1335
Largest diff. peak/hole / e Å^{-3}	0.35/-0.56

checkCIF/PLATON report

Structure factors have been supplied for datablock(s) 20220408-pcx-zb-154

THIS REPORT IS FOR GUIDANCE ONLY. IF USED AS PART OF A REVIEW PROCEDURE FOR PUBLICATION, IT SHOULD NOT REPLACE THE EXPERTISE OF AN EXPERIENCED CRYSTALLOGRAPHIC REFEREE.

No syntax errors found. CIF dictionary Interpreting this report

Datablock: 20220408-pcx-zb-154

Bond precision:	C-C = 0.0020 A		Wavelength=1.54184
Cell:	a=13.0482(2)	b=7.5185(1)	c=20.1642(3)
	alpha=90	beta=92.986(1)	gamma=90
Temperature:	293 K		

	Calculated	Reported
Volume	1975.48(5)	1975.48(5)
Space group	P 21/c	P 1 21/c 1
Hall group	-P 2ybc	-P 2ybc
Moiety formula	C21 H24 N4 O2 S	C21 H24 N4 O2 S
Sum formula	C21 H24 N4 O2 S	C21 H24 N4 O2 S
Mr	396.50	396.50
Dx,g cm-3	1.333	1.333
Z	4	4
Mu (mm-1)	1.655	1.655
F000	840.0	840.0
F000'	843.59	
h,k,lmax	16,9,25	16,9,25
Nref	4102	3926
Tmin,Tmax		
Tmin'		

Correction method= Not given

Data completeness= 0.957 Theta(max)= 75.621

R(reflections)= 0.0357(3578) wR2(reflections)= 0.1068(3926)

S = 1.087 Npar= 256

The following ALERTS were generated. Each ALERT has the format
test-name_ALERT_alert-type_alert-level.
Click on the hyperlinks for more details of the test.

Alert level C

```
PLAT052_ALERT_1_C Info on Absorption Correction Method   Not Given     Please Do !
PLAT053_ALERT_1_C Minimum Crystal Dimension Missing (or Error) ...     Please Check
PLAT054_ALERT_1_C Medium  Crystal Dimension Missing (or Error) ...     Please Check
PLAT055_ALERT_1_C Maximum Crystal Dimension Missing (or Error) ...     Please Check
PLAT911_ALERT_3_C Missing FCF Refl Between Thmin & STh/L=    0.600         13 Report
```

Alert level G

```
PLAT007_ALERT_5_G Number of Unrefined Donor-H Atoms .............           1 Report
PLAT199_ALERT_1_G Reported _cell_measurement_temperature ..... (K)        293 Check
PLAT200_ALERT_1_G Reported   _diffrn_ambient_temperature ..... (K)        293 Check
PLAT883_ALERT_1_G No Info/Value for _atom_sites_solution_primary .     Please Do !
PLAT912_ALERT_4_G Missing # of FCF Reflections Above STh/L=  0.600        164 Note
PLAT941_ALERT_3_G Average HKL Measurement Multiplicity ..........         3.6 Low
PLAT978_ALERT_2_G Number C-C Bonds with Positive Residual Density.          9 Info
```

0 **ALERT level A** = Most likely a serious problem - resolve or explain
0 **ALERT level B** = A potentially serious problem, consider carefully
5 **ALERT level C** = Check. Ensure it is not caused by an omission or oversight
7 **ALERT level G** = General information/check it is not something unexpected

7 ALERT type 1 CIF construction/syntax error, inconsistent or missing data
1 ALERT type 2 Indicator that the structure model may be wrong or deficient
2 ALERT type 3 Indicator that the structure quality may be low
1 ALERT type 4 Improvement, methodology, query or suggestion
1 ALERT type 5 Informative message, check

It is advisable to attempt to resolve as many as possible of the alerts in all categories. Often the minor alerts point to easily fixed oversights, errors and omissions in your CIF or refinement strategy, so attention to these fine details can be worthwhile. In order to resolve some of the more serious problems it may be necessary to carry out additional measurements or structure refinements. However, the purpose of your study may justify the reported deviations and the more serious of these should normally be commented upon in the discussion or experimental section of a paper or in the "special_details" fields of the CIF. checkCIF was carefully designed to identify outliers and unusual parameters, but every test has its limitations and alerts that are not important in a particular case may appear. Conversely, the absence of alerts does not guarantee there are no aspects of the results needing attention. It is up to the individual to critically assess their own results and, if necessary, seek expert advice.

Publication of your CIF in IUCr journals

A basic structural check has been run on your CIF. These basic checks will be run on all CIFs submitted for publication in IUCr journals (*Acta Crystallographica*, *Journal of Applied Crystallography*, *Journal of Synchrotron Radiation*); however, if you intend to submit to *Acta Crystallographica Section C* or *E* or *IUCrData*, you should make sure that full publication checks are run on the final version of your CIF prior to submission.

Publication of your CIF in other journals

Please refer to the *Notes for Authors* of the relevant journal for any special instructions relating to CIF submission.

PLATON version of 06/07/2023; check.def file version of 30/06/2023

Datablock 20220408-pcx-zb-154 - ellipsoid plot

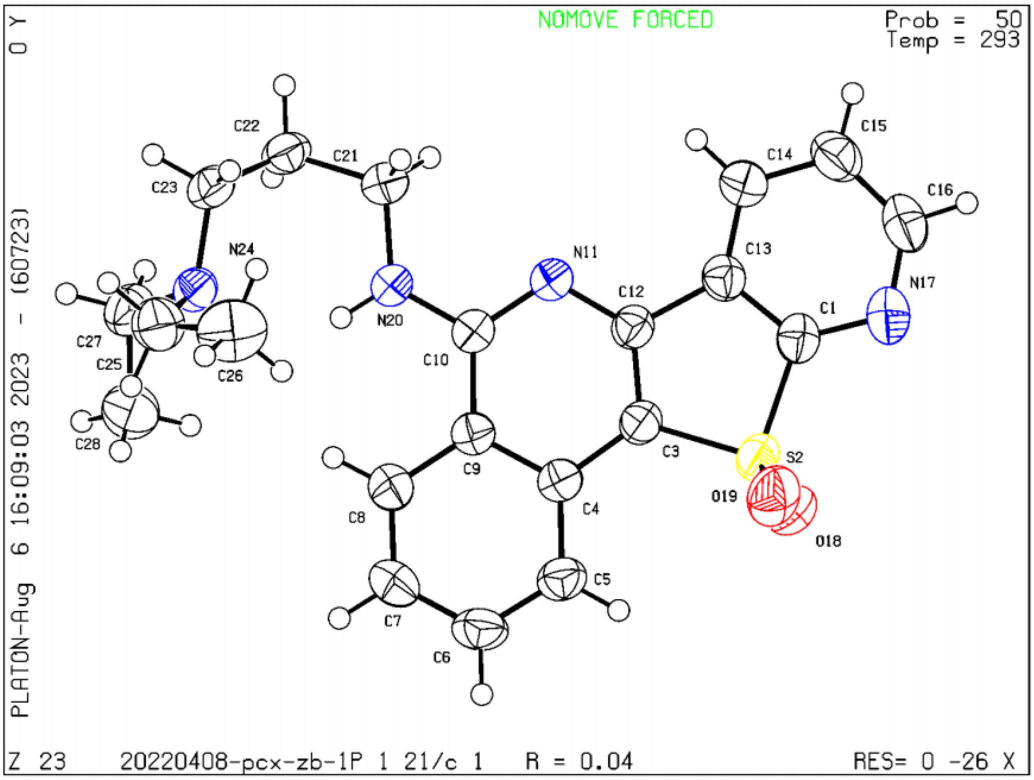

checkCIF/PLATON report

Structure factors have been supplied for datablock(s) zbb-157_auto

THIS REPORT IS FOR GUIDANCE ONLY. IF USED AS PART OF A REVIEW PROCEDURE FOR PUBLICATION, IT SHOULD NOT REPLACE THE EXPERTISE OF AN EXPERIENCED CRYSTALLOGRAPHIC REFEREE.

No syntax errors found. CIF dictionary Interpreting this report

Datablock: zbb-157_auto

Bond precision: C-C = 0.0027 A Wavelength=1.54184

Cell:	a=12.3082(2)	b=10.5428(1)	c=16.2830(2)
	alpha=90	beta=111.573(2)	gamma=90
Temperature:	298 K		

	Calculated	Reported
Volume	1964.92(5)	1964.92(5)
Space group	P 21/n	P 1 21/n 1
Hall group	-P 2yn	-P 2yn
Moiety formula	C21 H22 N4 O3 S	C21 H22 N4 O3 S
Sum formula	C21 H22 N4 O3 S	C21 H22 N4 O3 S
Mr	410.49	410.48
Dx,g cm-3	1.388	1.388
Z	4	4
Mu (mm-1)	1.726	1.726
F000	864.0	864.0
F000'	867.78	
h,k,lmax	15,13,20	15,13,20
Nref	4169	3966
Tmin,Tmax	0.813,0.841	0.387,1.000
Tmin'	0.708	

Correction method= # Reported T Limits: Tmin=0.387 Tmax=1.000
AbsCorr = MULTI-SCAN

Data completeness= 0.951 Theta(max)= 77.121

R(reflections)= 0.0426(3552) wR2(reflections)= 0.1233(3966)

S = 1.061 Npar= 263

```
The following ALERTS were generated. Each ALERT has the format
       test-name_ALERT_alert-type_alert-level.
Click on the hyperlinks for more details of the test.
```

Alert level C

```
PLAT911_ALERT_3_C Missing FCF Refl Between Thmin & STh/L=    0.600         10 Report
                 2  2  0,  13  4  0,   5  0  1, -13  5  2,   6  0  2, -12  7  3,
                 7  8  5,  -2 12  6,  -1 12  6,   7  5  6,
```

Alert level G

```
PLAT007_ALERT_5_G Number of Unrefined Donor-H Atoms .............          1 Report
              H008
PLAT142_ALERT_4_G s.u. on b - Axis Small or Missing .............    0.00010 Ang.
PLAT398_ALERT_2_G Deviating  C-O-C   Angle From 120 for O006     .     109.0 Degree
PLAT720_ALERT_4_G Number of Unusual/Non-Standard Labels .........         51 Note
              S001    O002    O003    N004    N005    O006    N007    N008
              H008    C009    C00A    C00B    C00C    C00D    H00D    C00E
              C00F    C00G    C00H    H00H    C00I    H00I    C00J    H00A
              H00B    C00K    H00C    H00E    C00L    H00L    C00M    H00M
              C00N    H00F    H00G    C00O    H00J    H00K    C00P    H00N
              H00O    C00Q    H00Q    C00R    H00R    C00S    H00P    H00S
              C00T    H00T    H00U
PLAT912_ALERT_4_G Missing # of FCF Reflections Above STh/L=  0.600        193 Note
PLAT933_ALERT_2_G Number of HKL-OMIT Records in Embedded .res File          2 Note
                 7  5  6,   7  8  5,
PLAT941_ALERT_3_G Average HKL Measurement Multiplicity ..........        4.4 Low
PLAT969_ALERT_5_G The 'Henn et al.' R-Factor-gap value ..........      4.337 Note
              Predicted wR2: Based on SigI**2  2.84 or SHELX Weight 11.62
PLAT978_ALERT_2_G Number C-C Bonds with Positive Residual Density.         11 Info
```

0 **ALERT level A** = Most likely a serious problem - resolve or explain
0 **ALERT level B** = A potentially serious problem, consider carefully
1 **ALERT level C** = Check. Ensure it is not caused by an omission or oversight
9 **ALERT level G** = General information/check it is not something unexpected

0 ALERT type 1 CIF construction/syntax error, inconsistent or missing data
3 ALERT type 2 Indicator that the structure model may be wrong or deficient
2 ALERT type 3 Indicator that the structure quality may be low
3 ALERT type 4 Improvement, methodology, query or suggestion
2 ALERT type 5 Informative message, check

It is advisable to attempt to resolve as many as possible of the alerts in all categories. Often the minor alerts point to easily fixed oversights, errors and omissions in your CIF or refinement strategy, so attention to these fine details can be worthwhile. In order to resolve some of the more serious problems it may be necessary to carry out additional measurements or structure refinements. However, the purpose of your study may justify the reported deviations and the more serious of these should normally be commented upon in the discussion or experimental section of a paper or in the "special_details" fields of the CIF. checkCIF was carefully designed to identify outliers and unusual parameters, but every test has its limitations and alerts that are not important in a particular case may appear. Conversely, the absence of alerts does not guarantee there are no aspects of the results needing attention. It is up to the individual to critically assess their own results and, if necessary, seek expert advice.

Publication of your CIF in IUCr journals

A basic structural check has been run on your CIF. These basic checks will be run on all CIFs submitted for publication in IUCr journals (*Acta Crystallographica*, *Journal of Applied Crystallography*, *Journal of Synchrotron Radiation*); however, if you intend to submit to *Acta Crystallographica Section C* or *E* or *IUCrData*, you should make sure that full publication checks are run on the final version of your CIF prior to submission.

Publication of your CIF in other journals

Please refer to the *Notes for Authors* of the relevant journal for any special instructions relating to CIF submission.

PLATON version of 22/08/2024; check.def file version of 21/08/2024

Datablock zbb-157_auto - ellipsoid plot

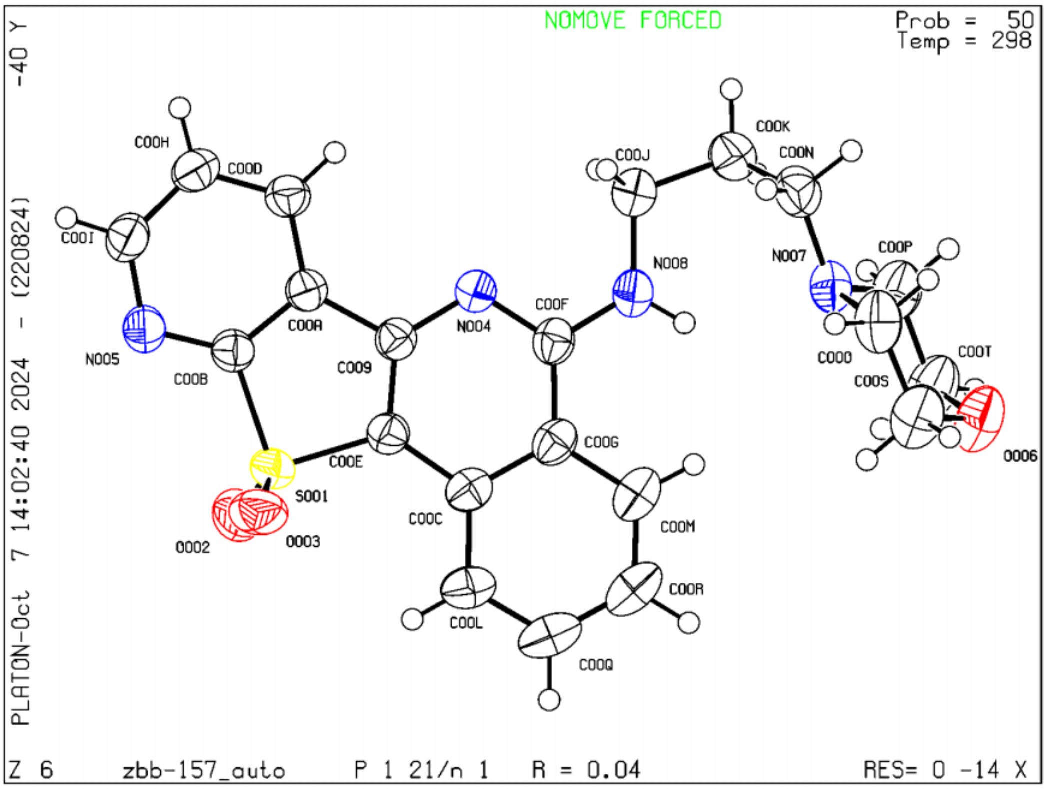

checkCIF/PLATON report

You have not supplied any structure factors. As a result the full set of tests cannot be run.

THIS REPORT IS FOR GUIDANCE ONLY. IF USED AS PART OF A REVIEW PROCEDURE FOR PUBLICATION, IT SHOULD NOT REPLACE THE EXPERTISE OF AN EXPERIENCED CRYSTALLOGRAPHIC REFEREE.

No syntax errors found. CIF dictionary Interpreting this report

Datablock: 20230721-pcx-zb-7be_auto

Bond precision:	C-C = 0.0022 A		Wavelength=1.54184
Cell:	a=17.6237(1)	b=13.2073(1)	c=17.6569(2)
	alpha=90	beta=91.413(1)	gamma=90
Temperature:	100 K		

	Calculated	Reported
Volume	4108.60(6)	4108.60(6)
Space group	P 21/n	P 1 21/n 1
Hall group	-P 2yn	-P 2yn
Moiety formula	C23 H26 N4 O2 S	2(C23 H26 N4 O2 S)
Sum formula	C23 H26 N4 O2 S	C46 H52 N8 O4 S2
Mr	422.54	845.07
Dx,g cm-3	1.366	1.366
Z	8	4
Mu (mm-1)	1.627	1.627
F000	1792.0	1792.0
F000'	1799.45	
h,k,lmax	22,16,22	22,16,22
Nref	8694	8522
Tmin,Tmax	0.711,0.850	0.373,1.000
Tmin'	0.585	

Correction method= # Reported T Limits: Tmin=0.373 Tmax=1.000
AbsCorr = MULTI-SCAN

Data completeness= 0.980 Theta(max)= 77.246

R(reflections)= 0.0467(7182) wR2(reflections)= 0.1335(8522)

S = 1.079 Npar= 545

The following ALERTS were generated. Each ALERT has the format
test-name_ALERT_alert-type_alert-level.
Click on the hyperlinks for more details of the test.

Alert level C

```
PLAT413_ALERT_2_C Short Inter XH3 .. XHn     H19A     ..H19A     .       2.10 Ang.
                                               1-x,2-y,1-z  =      3_676 Check
```

Alert level G

```
PLAT007_ALERT_5_G Number of Unrefined Donor-H Atoms .............          2 Report
PLAT045_ALERT_1_G Calculated and Reported Z Differ by a Factor ...          2 Check
PLAT142_ALERT_4_G s.u. on b - Axis Small or Missing .............    0.00010 Ang.
PLAT933_ALERT_2_G Number of HKL-OMIT Records in Embedded .res File          1 Note
```

0 **ALERT level A** = Most likely a serious problem - resolve or explain
0 **ALERT level B** = A potentially serious problem, consider carefully
1 **ALERT level C** = Check. Ensure it is not caused by an omission or oversight
4 **ALERT level G** = General information/check it is not something unexpected

1 ALERT type 1 CIF construction/syntax error, inconsistent or missing data
2 ALERT type 2 Indicator that the structure model may be wrong or deficient
0 ALERT type 3 Indicator that the structure quality may be low
1 ALERT type 4 Improvement, methodology, query or suggestion
1 ALERT type 5 Informative message, check

It is advisable to attempt to resolve as many as possible of the alerts in all categories. Often the minor alerts point to easily fixed oversights, errors and omissions in your CIF or refinement strategy, so attention to these fine details can be worthwhile. In order to resolve some of the more serious problems it may be necessary to carry out additional measurements or structure refinements. However, the purpose of your study may justify the reported deviations and the more serious of these should normally be commented upon in the discussion or experimental section of a paper or in the "special_details" fields of the CIF. checkCIF was carefully designed to identify outliers and unusual parameters, but every test has its limitations and alerts that are not important in a particular case may appear. Conversely, the absence of alerts does not guarantee there are no aspects of the results needing attention. It is up to the individual to critically assess their own results and, if necessary, seek expert advice.

Publication of your CIF in IUCr journals

A basic structural check has been run on your CIF. These basic checks will be run on all CIFs submitted for publication in IUCr journals (*Acta Crystallographica*, *Journal of Applied Crystallography*, *Journal of Synchrotron Radiation*); however, if you intend to submit to *Acta Crystallographica Section C* or *E* or *IUCrData*, you should make sure that full publication checks are run on the final version of your CIF prior to submission.

Publication of your CIF in other journals

Please refer to the *Notes for Authors* of the relevant journal for any special instructions relating to CIF submission.

PLATON version of 06/07/2023; check.def file version of 30/06/2023

Datablock 20230721-pcx-zb-7be_auto - ellipsoid plot

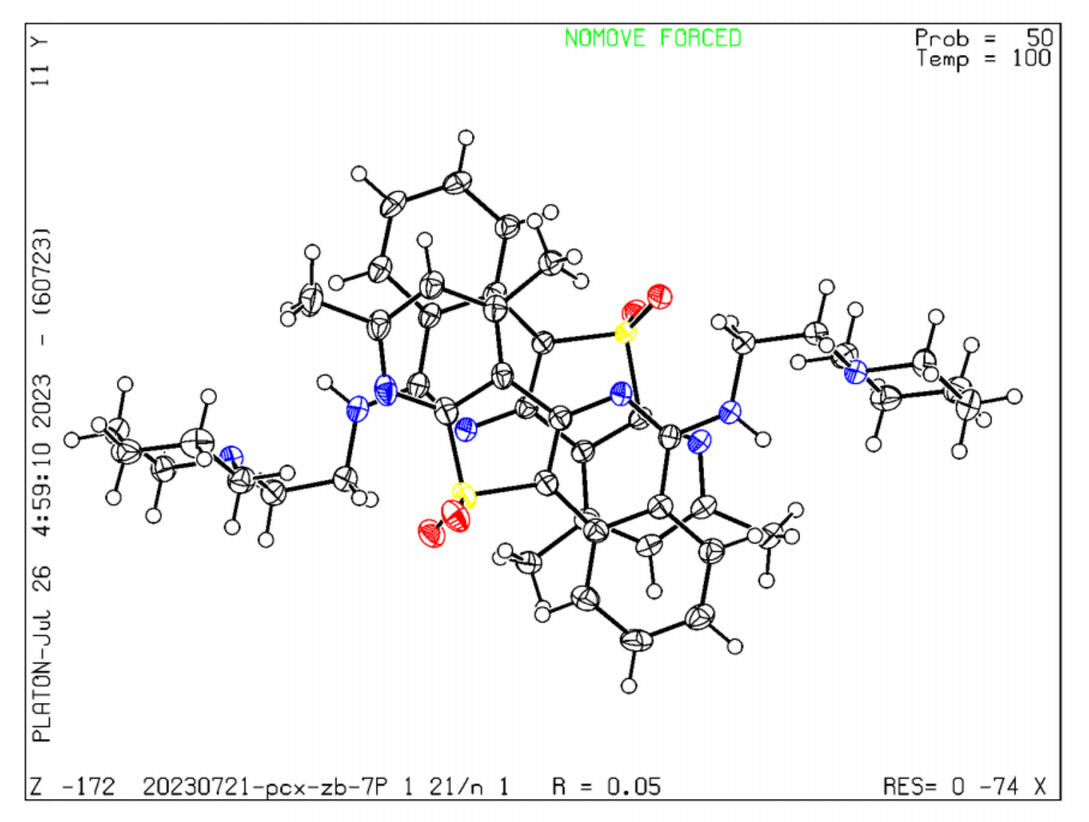

3. **Table S4.** Primers used in this study

Primer	Sequence
c-myc-F	5′-ATCCTGTCCGTCCAAGCA-3′
c-myc-R	5′-CGCACAAGAGTTCCGTAG-3′
GAPDH-F	5′-CAGGAGGCATTGCTGATGAT-3′
GAPDH-R	5′-GAAGGCTGGGGCTCATTT-3′

4. **Figure S4.** NMR and HRESIMS spectra for compounds **7ab**–**7al**, **7ba**–**7bj**, **7cb**, **7cf**–**7ci**.

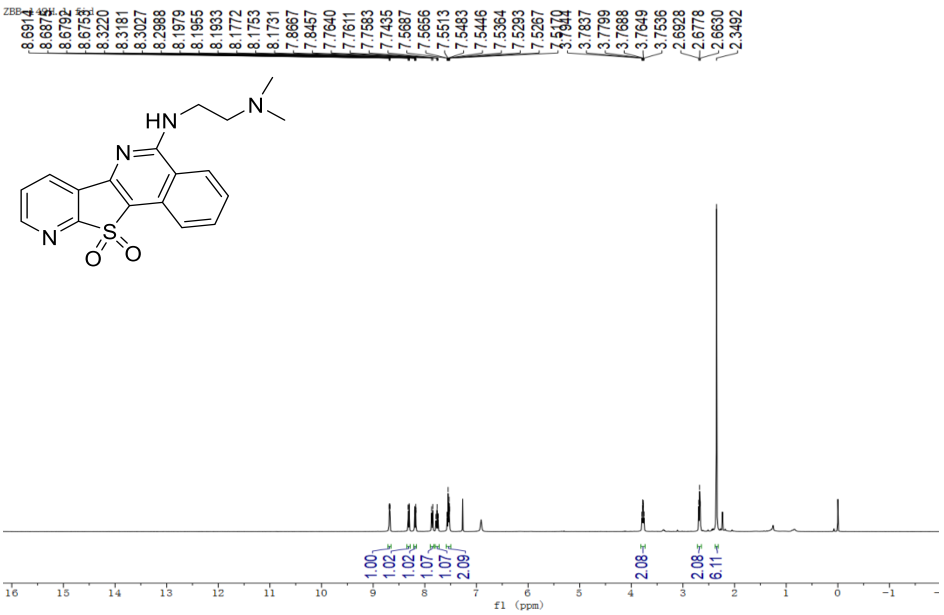

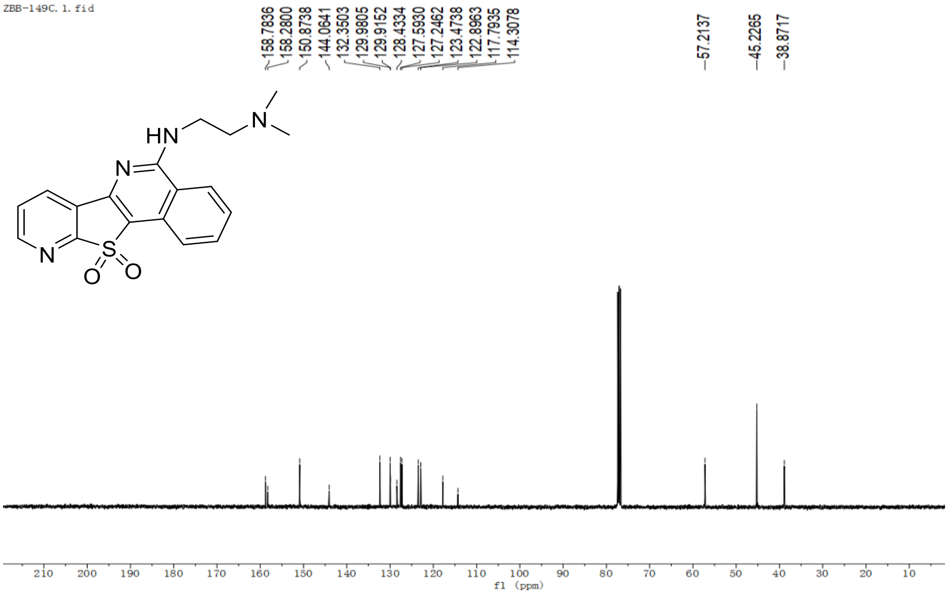

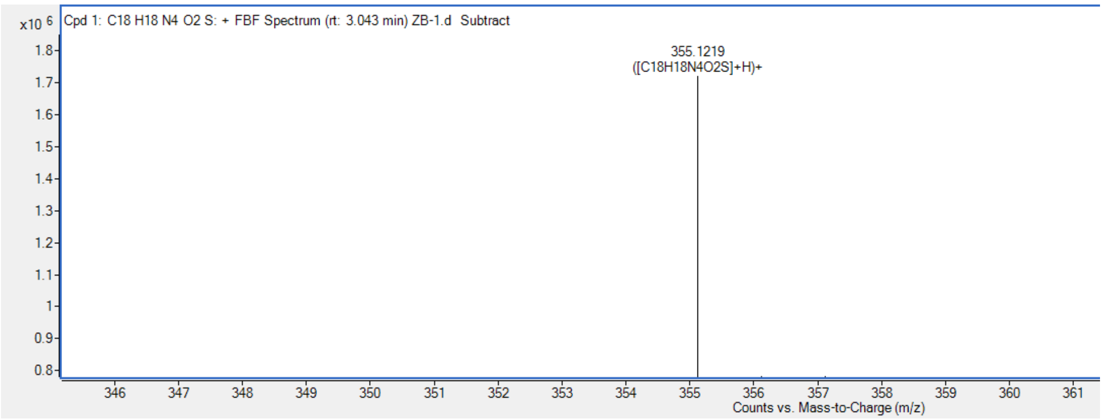

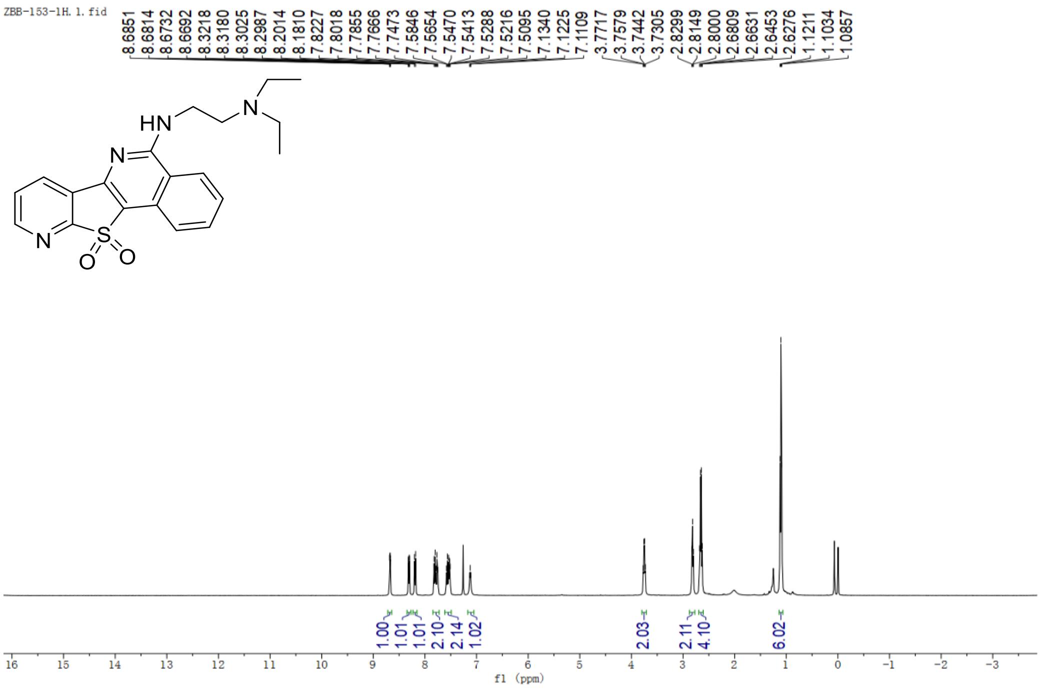
ZBB-153-1H. 1. fid
8.6851
8.6814
8.6732
8.6692
8.3218
8.3180
8.3025
8.2987
8.2014
8.1810
7.8227
7.8018
7.7855
7.7666
7.7473
7.5846
7.5654
7.5470
7.5413
7.5288
7.5216
7.5095
7.1340
7.1225
7.1109
3.7717
3.7579
3.7442
3.7305
2.8299
2.8149
2.8000
2.6809
2.6631
2.6453
2.6276
1.1211
1.1034
1.0857
HN
N
N
N
S
O
O
1.00
1.01
1.01
2.10
2.14
1.02
2.03
2.11
4.10
6.02
f1 (ppm)

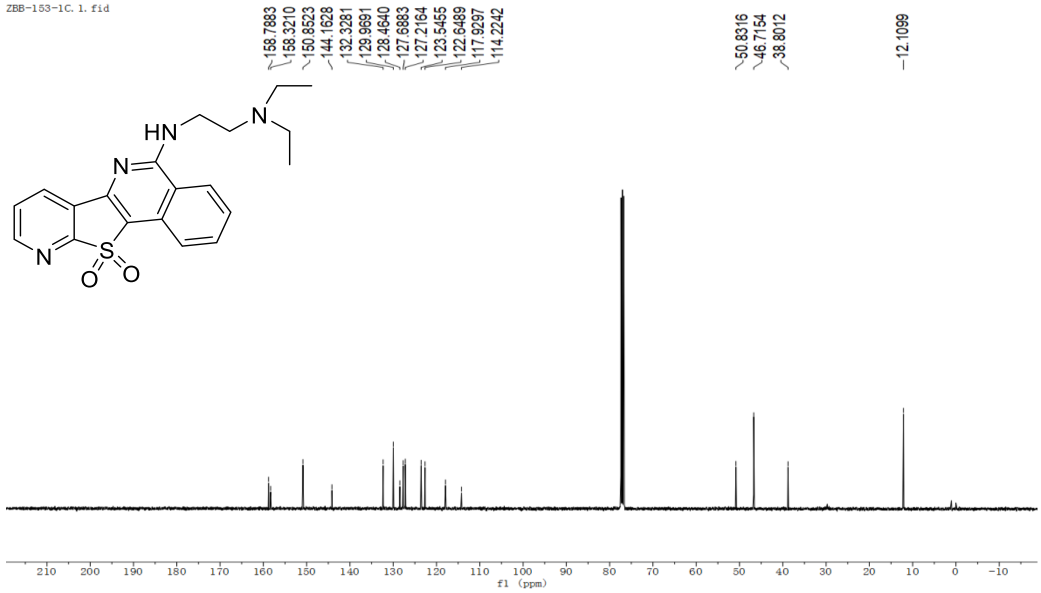
ZBB-153-1C. 1. fid
158.7883
158.3210
150.8523
144.1628
132.3281
129.9691
128.4640
127.6883
127.2164
123.5455
122.6489
117.9297
114.2242
50.8316
46.7154
38.8012
12.1099
HN
N
N
N
S
O
O
f1 (ppm)

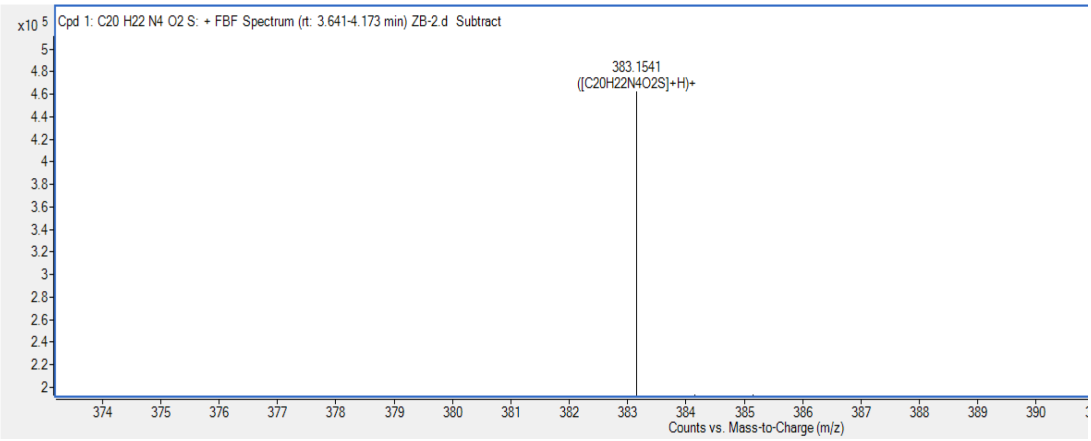
x10 5
Cpd 1: C20 H22 N4 O2 S: + FBF Spectrum (rt: 3.641-4.173 min) ZB-2.d Subtract
383.1541
([C20H22N4O2S]+H)+
Counts vs. Mass-to-Charge (m/z)

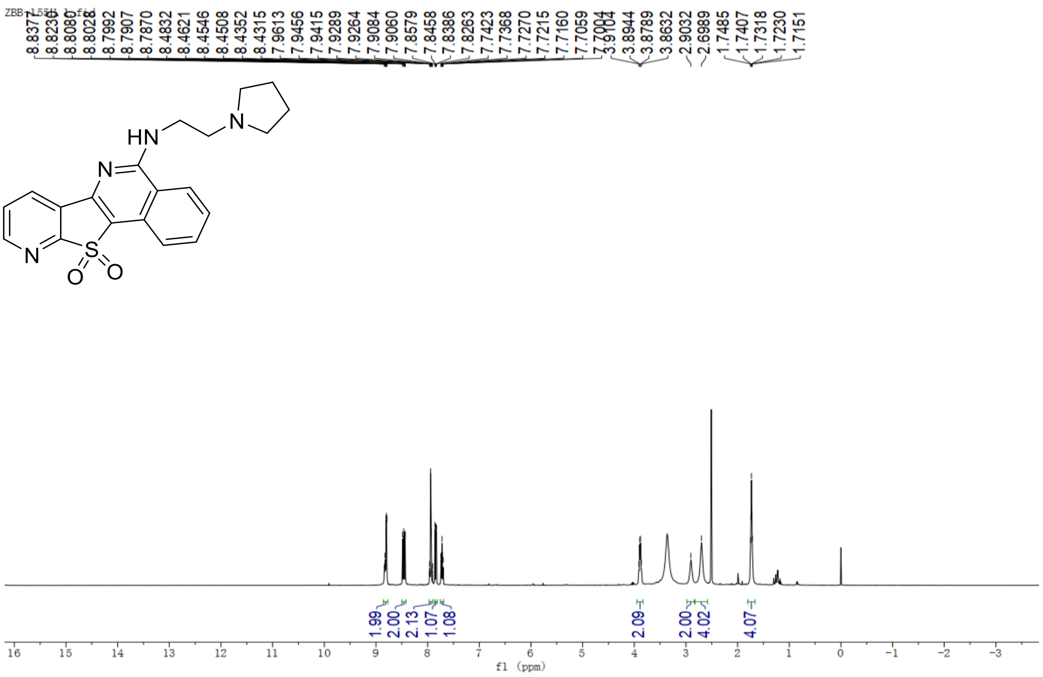
ZBB-155H.1.fid
8.8371 8.8230 8.8080 8.8028 8.7992 8.7907 8.7870 8.4832 8.4621 8.4546 8.4508 8.4352 8.4315 7.9613 7.9456 7.9415 7.9289 7.9264 7.9084 7.9060 7.8579 7.8458 7.8386 7.8263 7.7423 7.7368 7.7270 7.7215 7.7160 7.7059 7.7004 3.9104 3.8944 3.8789 3.8632 2.9032 2.6989 1.7485 1.7407 1.7318 1.7230 1.7151
HN
N
N
S
O
O
1.99 2.00 2.13 1.07 1.08 2.09 2.00 4.02 4.07
f1 (ppm)

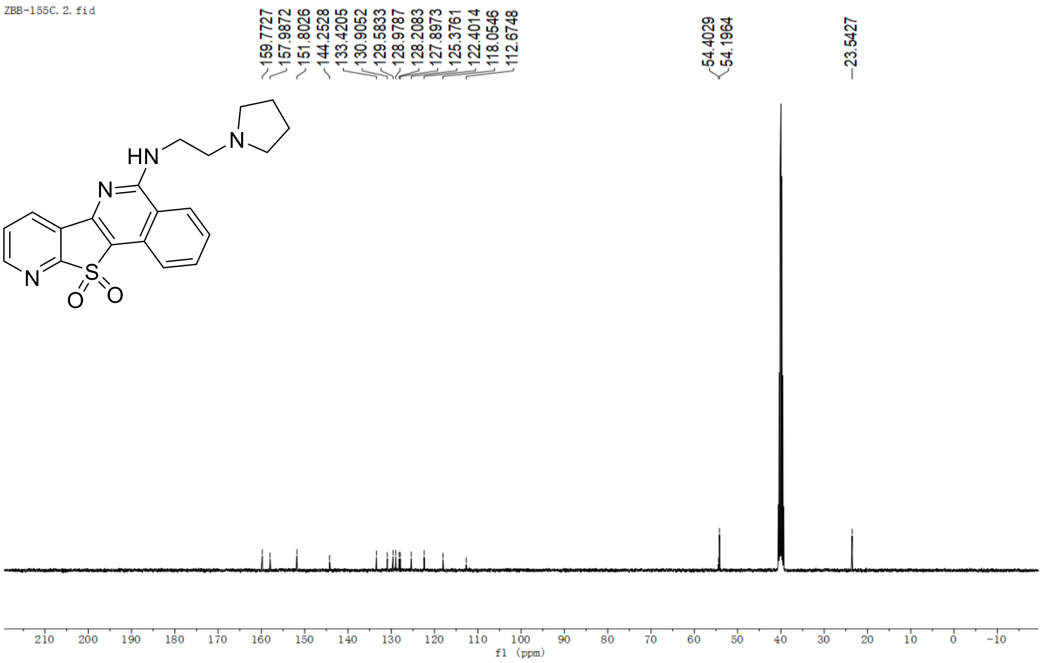
ZBB-155C.2.fid
159.7727 157.9872 151.8026 144.2528 133.4205 130.9052 129.5833 128.9787 128.2083 127.8973 125.3761 122.4014 118.0546 112.6748
54.4029 54.1964
23.5427
HN
N
N
S
O
O
f1 (ppm)

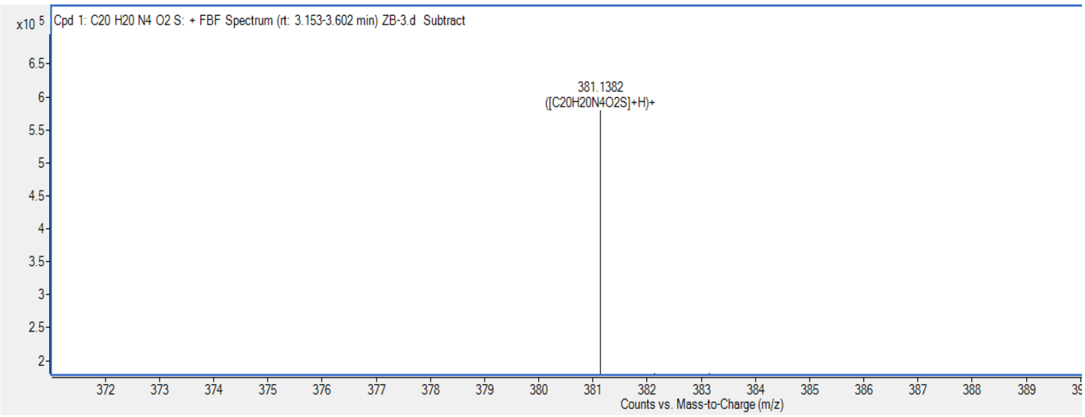
x10 5
Cpd 1: C20 H20 N4 O2 S: + FBF Spectrum (rt: 3.153-3.602 min) ZB-3.d Subtract
381.1382
([C20H20N4O2S]+H)+
Counts vs. Mass-to-Charge (m/z)

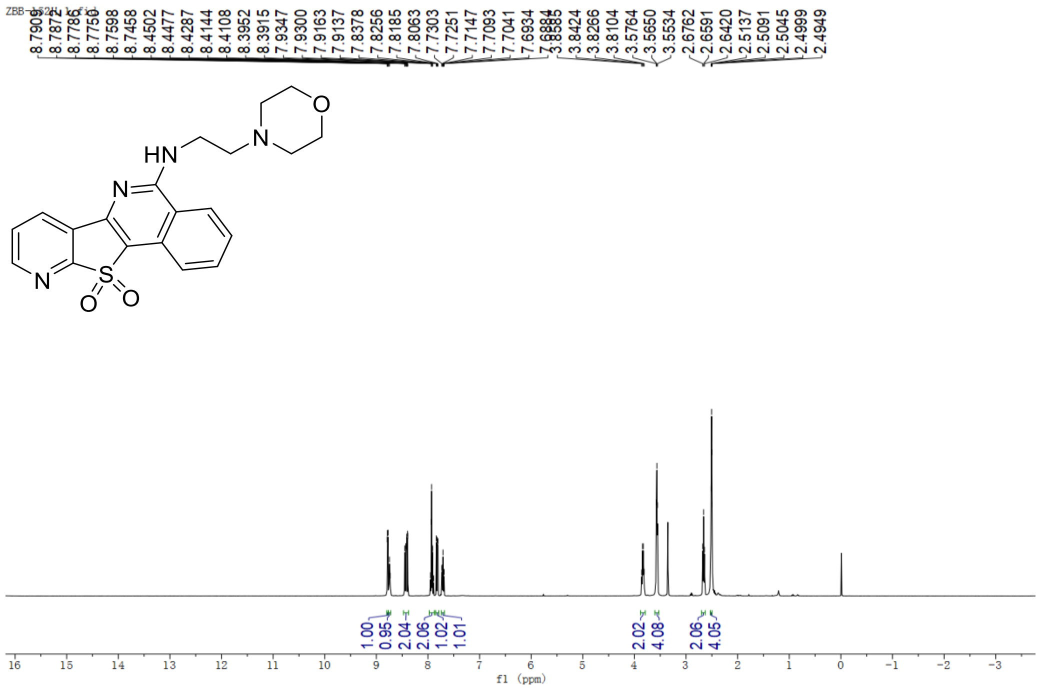
8.7871
8.7786
8.7750
8.7598
8.7458
8.4502
8.4477
8.4287
8.4144
8.4108
8.3952
8.3915
7.9347
7.9300
7.9163
7.9137
7.8378
7.8256
7.8185
7.8063
7.7303
7.7251
7.7147
7.7093
7.7041
7.6934
3.8424
3.8266
3.8104
3.5764
3.5650
3.5534
2.6762
2.6591
2.6420
2.5137
2.5091
2.5045
2.4999
2.4949
HN
N
O
N
N
S
O
O
1.00
0.95
2.04
2.06
1.02
1.01
2.02
4.08
2.06
4.05
f1 (ppm)

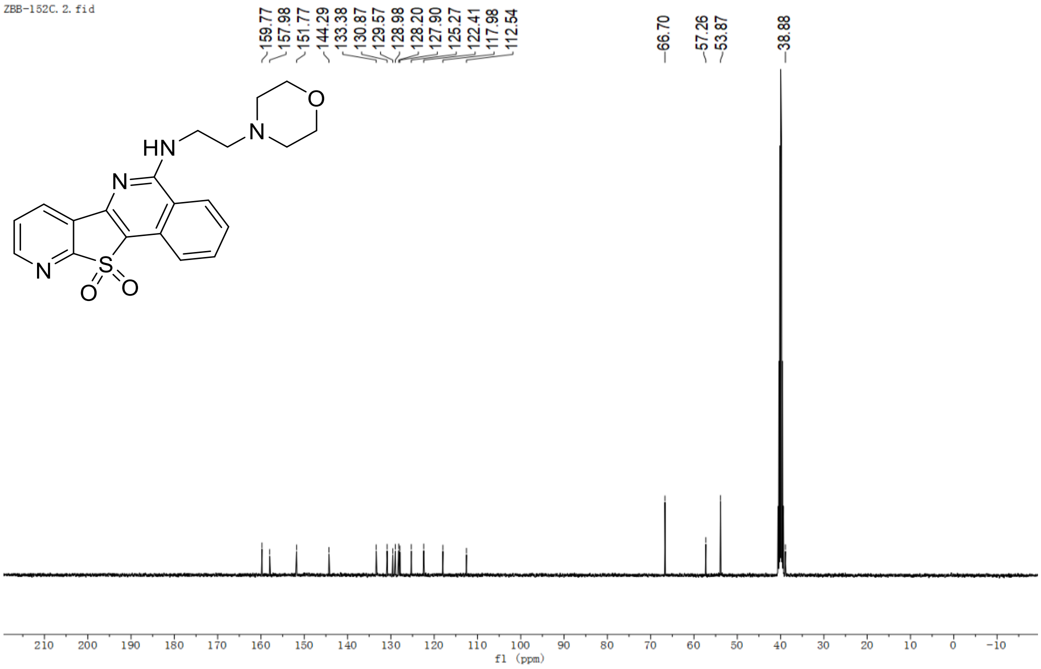
ZBB-152C.2.fid
159.77
157.98
151.77
144.29
133.38
130.87
129.57
128.98
128.20
127.90
125.27
122.41
117.98
112.54
66.70
57.26
53.87
38.88
HN
N
O
N
N
S
O
O
f1 (ppm)

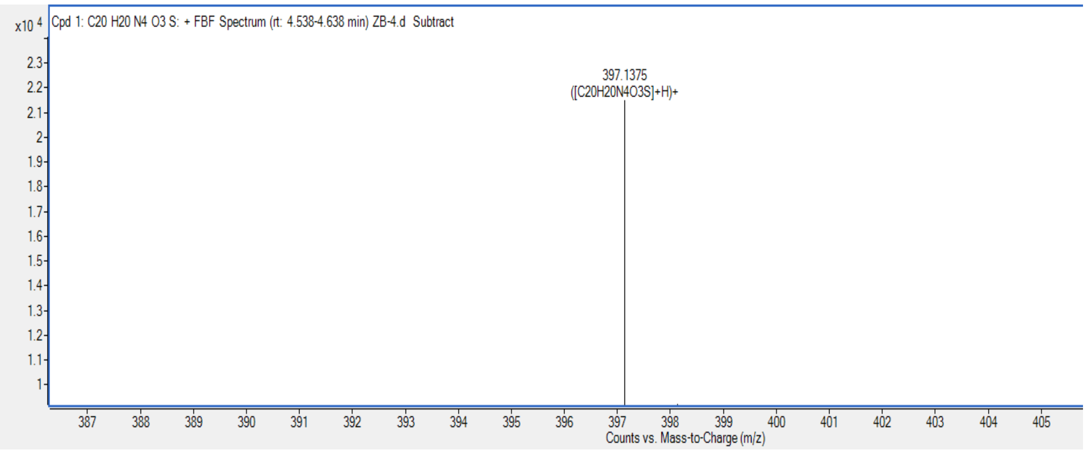
x10 4
Cpd 1: C20 H20 N4 O3 S: + FBF Spectrum (rt: 4.538-4.638 min) ZB-4.d Subtract
397.1375
([C20H20N4O3S]+H)+
Counts vs. Mass-to-Charge (m/z)

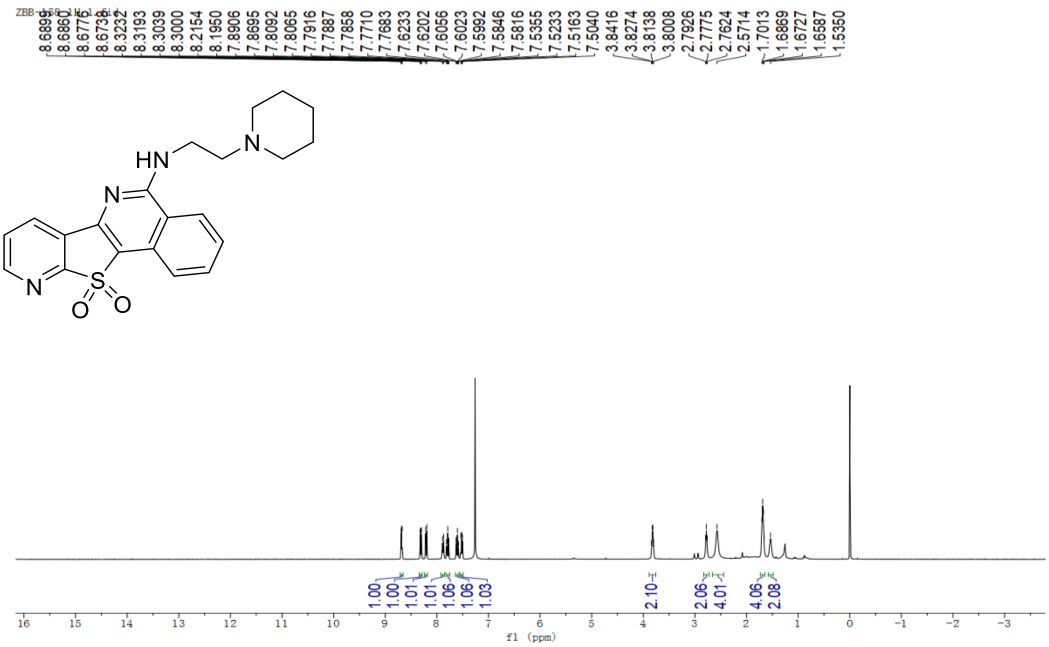
8.6899
8.6860
8.6776
8.6736
8.3232
8.3193
8.3039
8.3000
8.2154
8.1950
7.8906
7.8695
7.8092
7.8065
7.7916
7.7887
7.7858
7.7710
7.7683
7.6233
7.6202
7.6056
7.6023
7.5992
7.5846
7.5816
7.5355
7.5233
7.5163
7.5040
3.8416
3.8274
3.8138
3.8008
2.7926
2.7775
2.7624
2.5714
1.7013
1.6869
1.6727
1.6587
1.5350
HN
N
N
N
S
O
O
1.00
1.00
1.01
1.01
1.06
1.06
1.03
2.10
2.06
4.01
4.06
2.08
f1 (ppm)

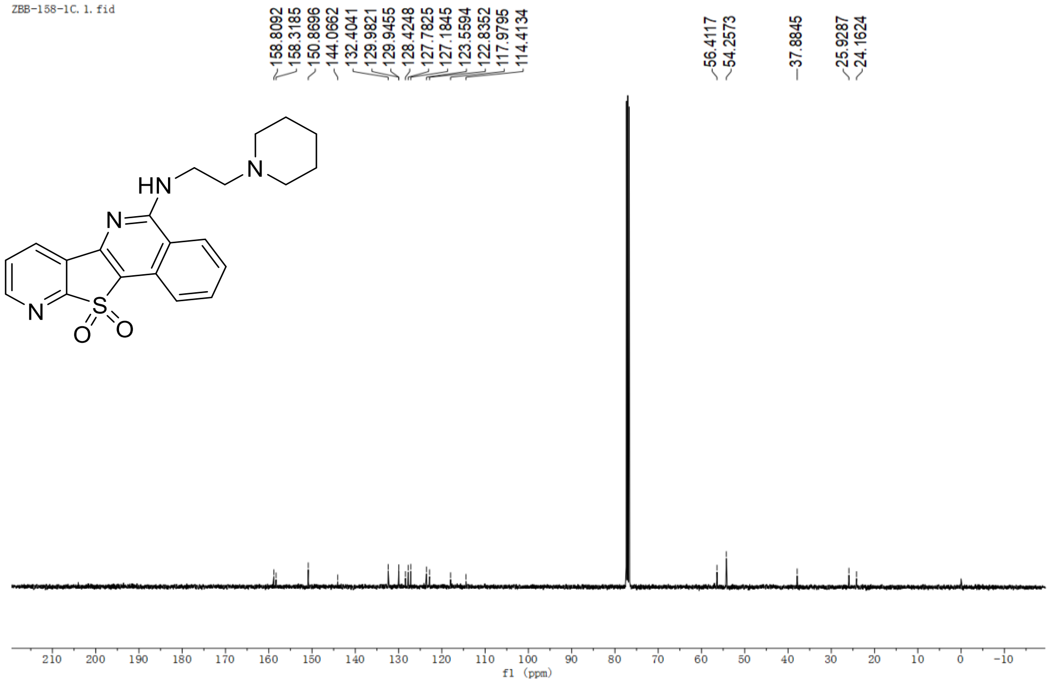
ZBB-158-1C. 1. fid
158.8092
158.3185
150.8696
144.0662
132.4041
129.9821
129.9455
128.4248
127.7825
127.1845
123.5594
122.8352
117.9795
114.4134
56.4117
54.2573
37.8845
25.9287
24.1624
HN
N
N
N
S
O
O
f1 (ppm)

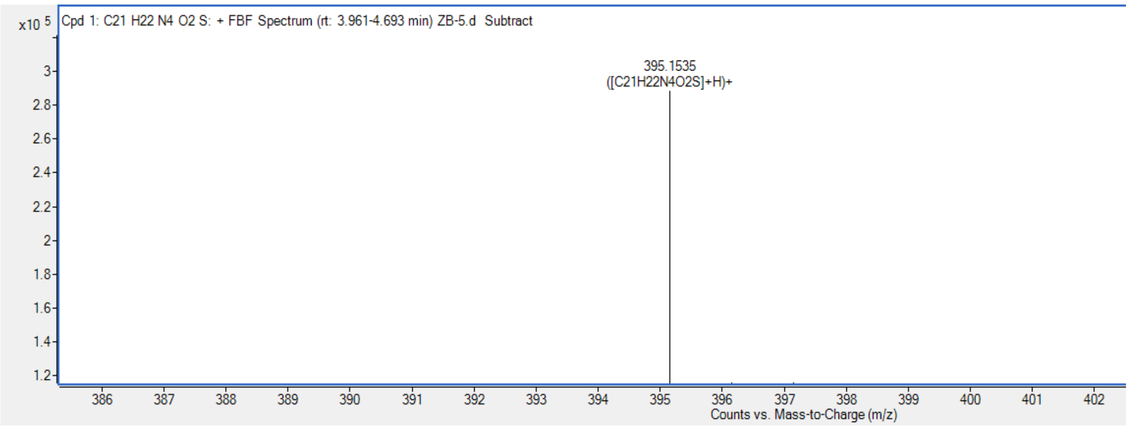
x10 5
Cpd 1: C21 H22 N4 O2 S: + FBF Spectrum (rt: 3.961-4.693 min) ZB-5.d Subtract
395.1535
([C21H22N4O2S]+H)+
Counts vs. Mass-to-Charge (m/z)

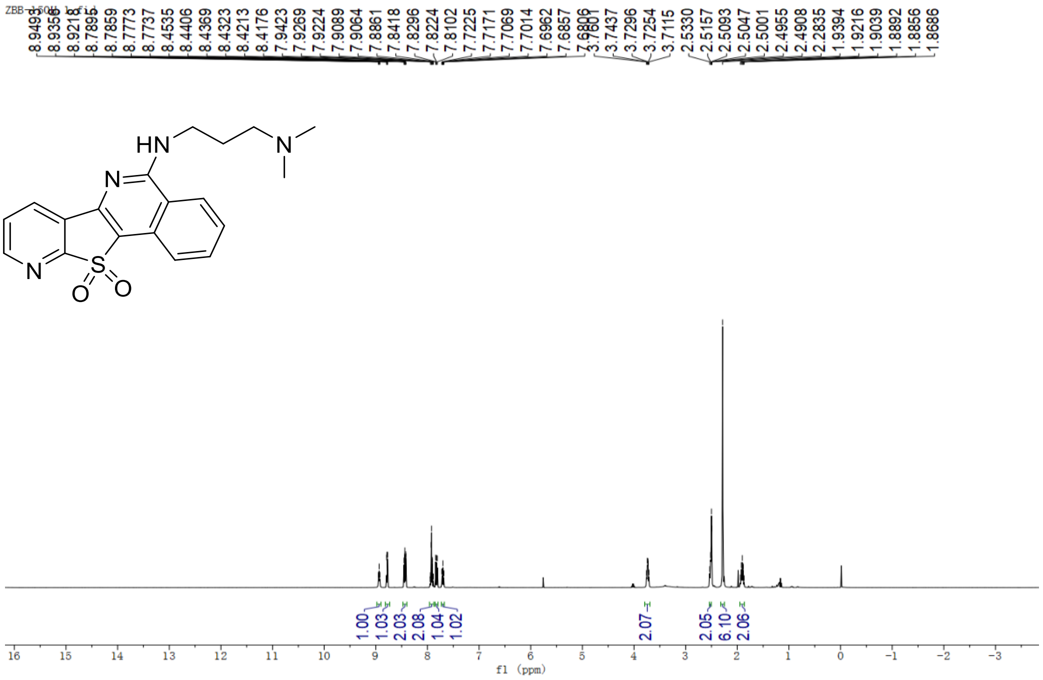
8.9493
8.9356
8.9218
8.7895
8.7859
8.7773
8.7737
8.4535
8.4406
8.4369
8.4323
8.4213
8.4176
7.9423
7.9269
7.9224
7.9089
7.9064
7.8861
7.8418
7.8296
7.8224
7.8102
7.7225
7.7171
7.7069
7.7014
7.6962
7.6857
7.6806
3.7601
3.7437
3.7296
3.7254
3.7115
2.5330
2.5157
2.5093
2.5047
2.5001
2.4955
2.4908
2.2835
1.9394
1.9216
1.9039
1.8892
1.8856
1.8686
HN
N
N
N
S
O
O
1.00
1.03
2.03
2.08
1.04
1.02
2.07
2.05
6.10
2.06
f1 (ppm)

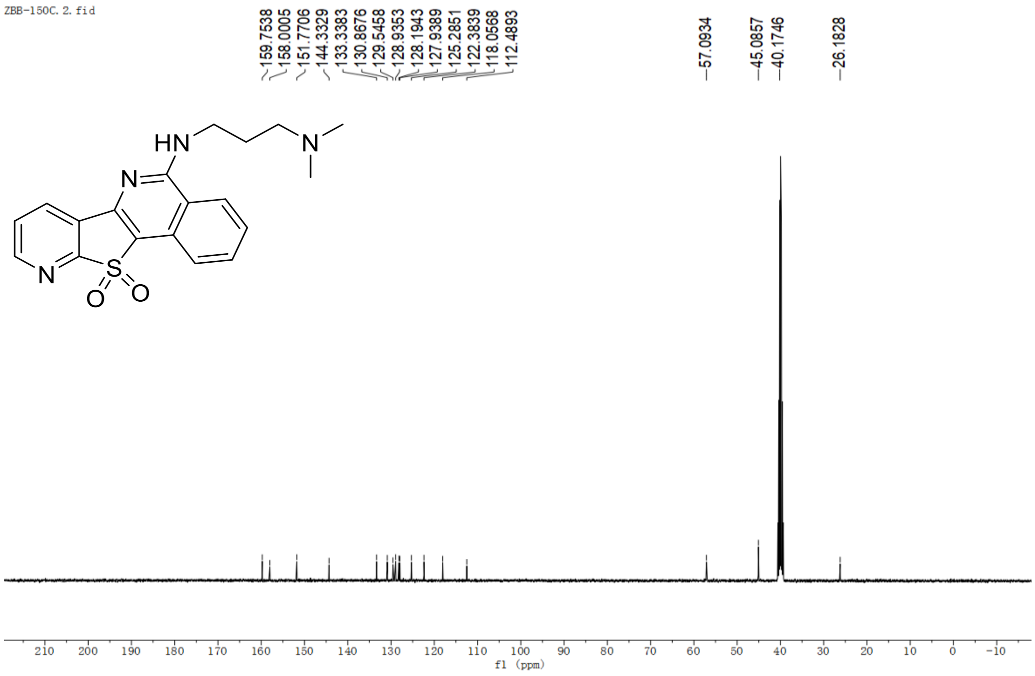
ZBB-150C. 2. fid
159.7538
158.0005
151.7706
144.3329
133.3383
130.8676
129.5458
128.9353
128.1943
127.9389
125.2851
122.3839
118.0568
112.4893
57.0934
45.0857
40.1746
26.1828
HN
N
N
N
S
O
O
f1 (ppm)

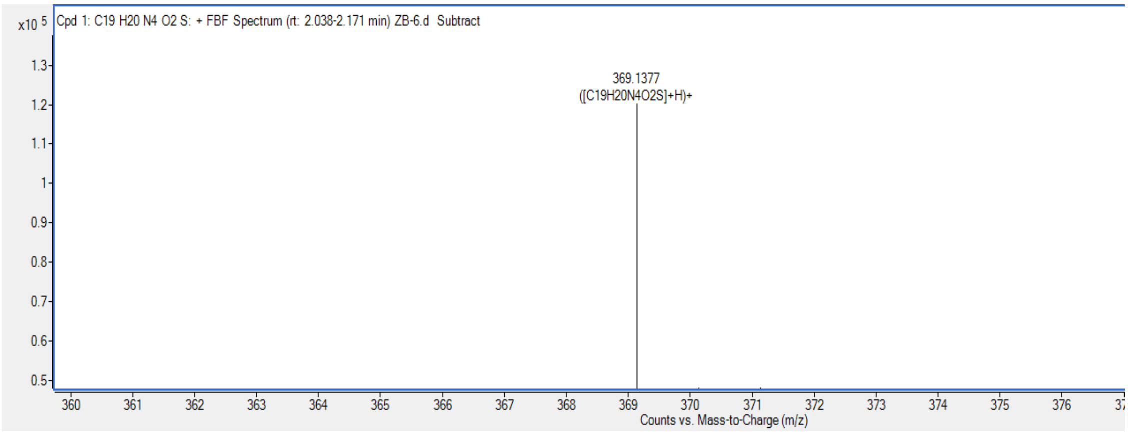
x10 5
Cpd 1: C19 H20 N4 O2 S: + FBF Spectrum (rt: 2.038-2.171 min) ZB-6.d Subtract
369.1377
([C19H20N4O2S]+H)+
Counts vs. Mass-to-Charge (m/z)

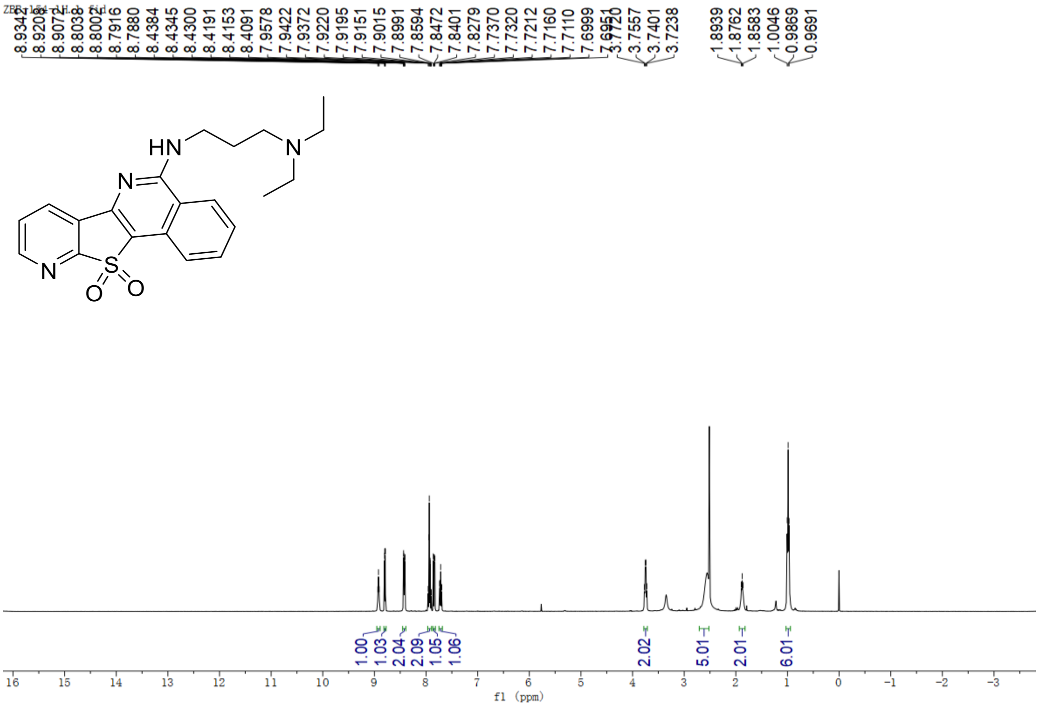
8.9342
8.9208
8.9073
8.8038
8.8002
8.7916
8.7880
8.4384
8.4345
8.4300
8.4191
8.4153
8.4091
7.9578
7.9422
7.9372
7.9220
7.9195
7.9151
7.9015
7.8991
7.8594
7.8472
7.8401
7.8279
7.7370
7.7320
7.7212
7.7160
7.7110
7.6999
3.7557
3.7401
3.7238
1.8939
1.8762
1.8583
1.0046
0.9869
0.9691
HN
N
N
N
S
O
O
1.00
1.03
2.04
2.09
1.05
1.06
2.02
5.01
2.01
6.01
f1 (ppm)

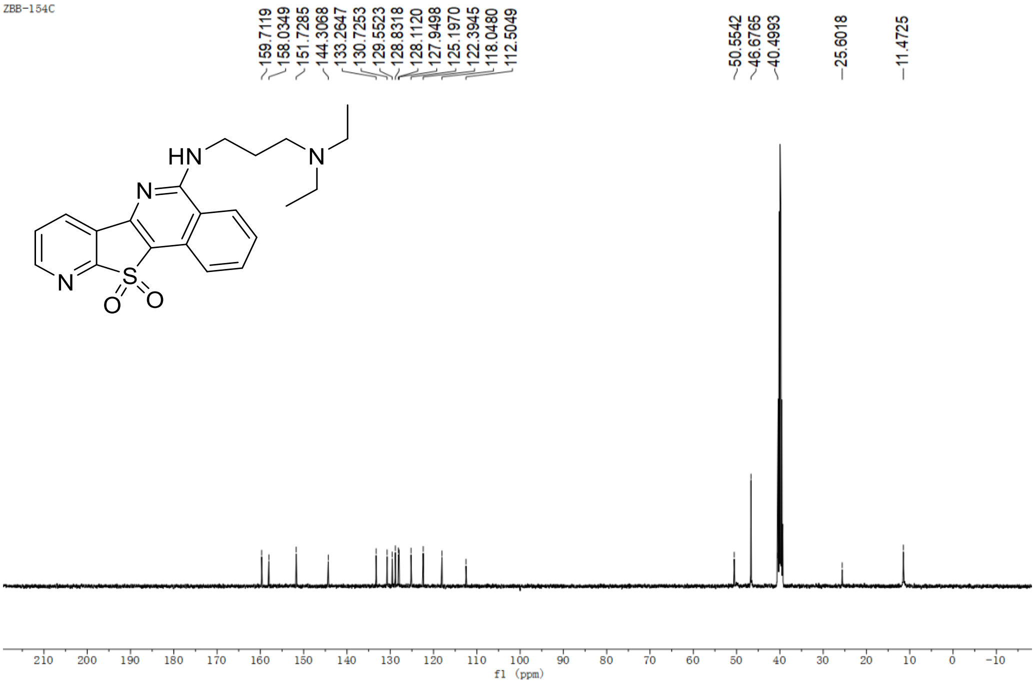
ZBB-154C
159.7119
158.0349
151.7285
144.3068
133.2647
130.7253
129.5523
128.8318
128.1120
127.9498
125.1970
122.3945
118.0480
112.5049
50.5542
46.6765
40.4993
25.6018
11.4725
HN
N
N
N
S
O
O
f1 (ppm)

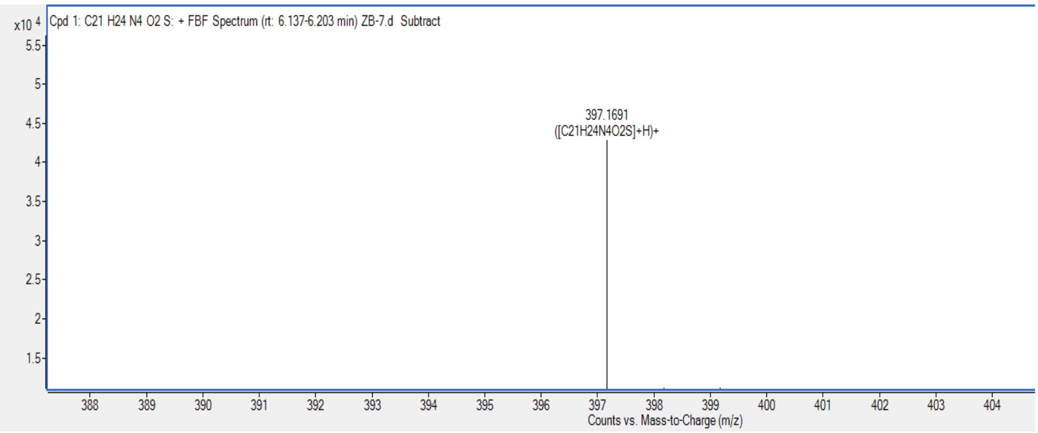
x10 4
Cpd 1: C21 H24 N4 O2 S: + FBF Spectrum (rt: 6.137-6.203 min) ZB-7.d Subtract
397.1691
([C21H24N4O2S]+H)+
Counts vs. Mass-to-Charge (m/z)

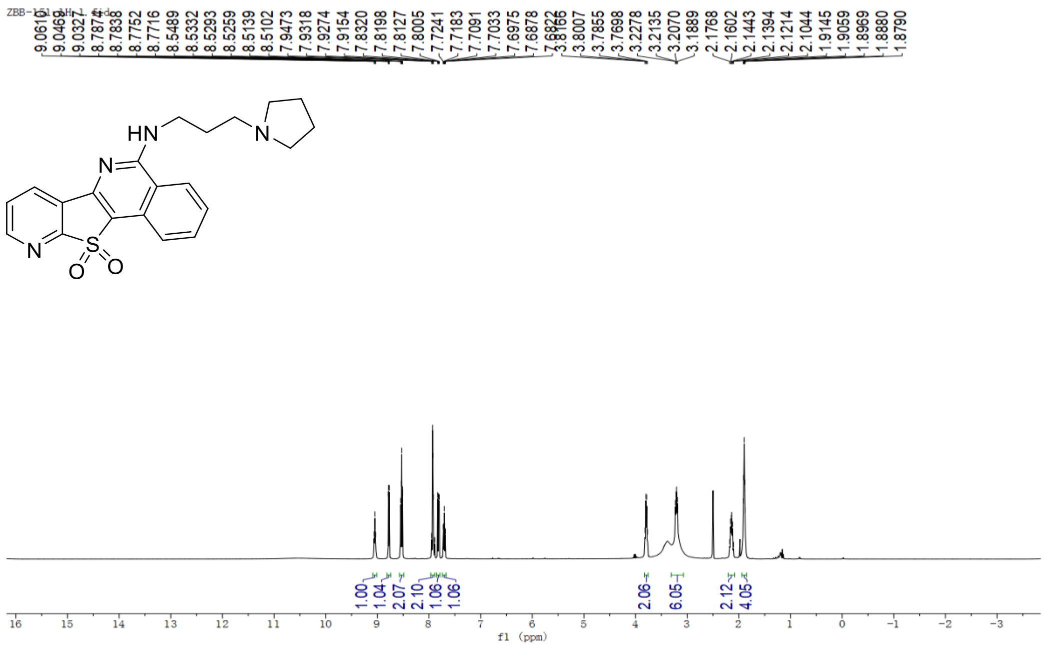
9.0610
9.0469
9.0327
8.7874
8.7838
8.7752
8.7716
8.5489
8.5332
8.5293
8.5259
8.5139
8.5102
7.9473
7.9318
7.9274
7.9154
7.8320
7.8198
7.8127
7.8005
7.7241
7.7183
7.7091
7.7033
7.6975
7.6878
7.6822
3.8166
3.8007
3.7855
3.7698
3.2278
3.2135
3.2070
3.1889
2.1768
2.1602
2.1443
2.1394
2.1214
2.1044
1.9145
1.9059
1.8969
1.8880
1.8790
HN
N
N
N
S
O
O
1.00
1.04
2.07
2.10
1.06
1.06
2.06
6.05
2.12
4.05
f1 (ppm)

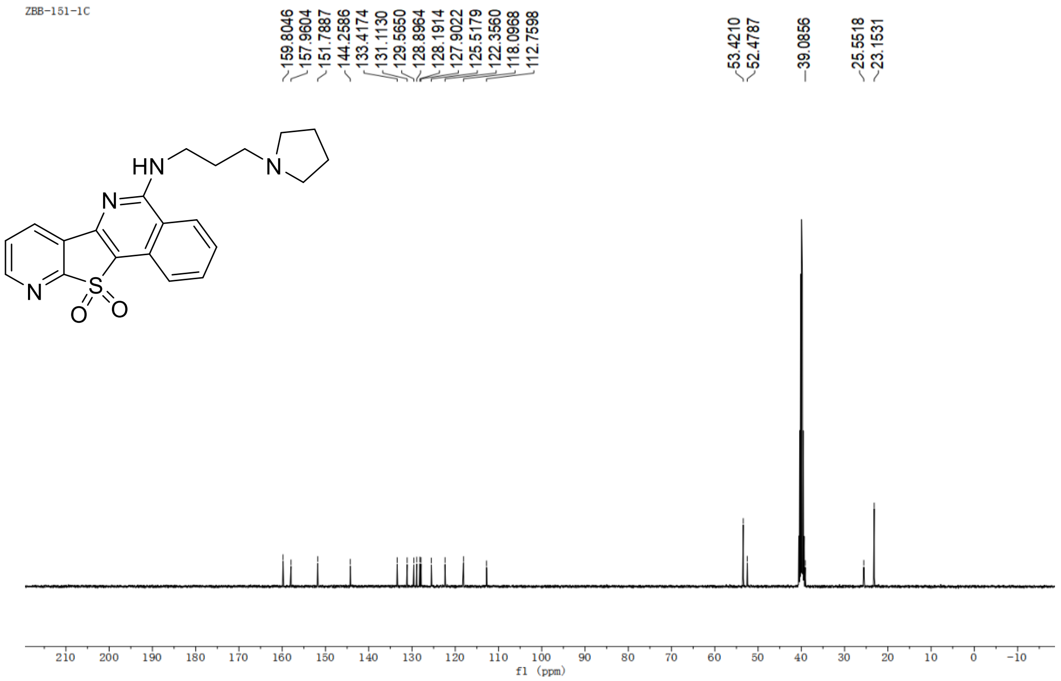
ZBB-151-1C
159.8046
157.9604
151.7887
144.2586
133.4174
131.1130
129.5650
128.8964
128.1914
127.9022
125.5179
122.3560
118.0968
112.7598
53.4210
52.4787
39.0856
25.5518
23.1531
HN
N
N
N
S
O
O
f1 (ppm)

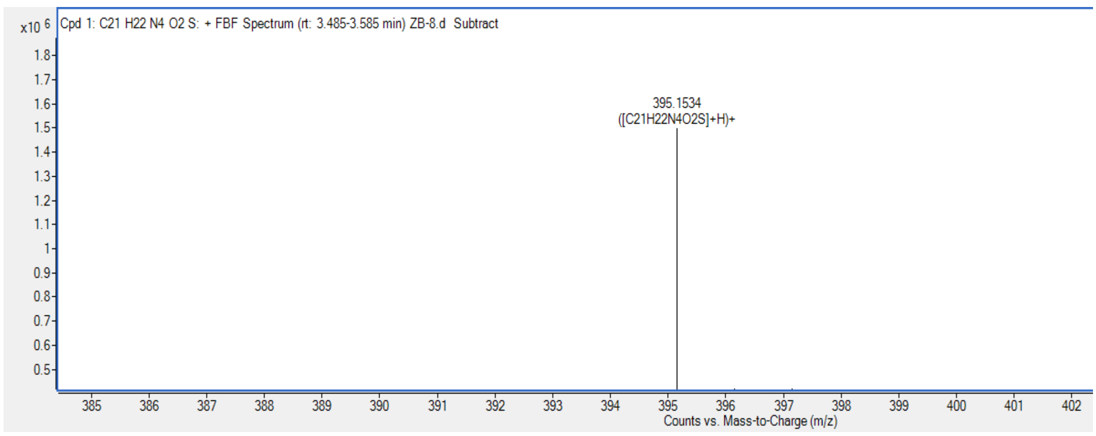
x10 6
Cpd 1: C21 H22 N4 O2 S: + FBF Spectrum (rt: 3.485-3.585 min) ZB-8.d Subtract
395.1534
([C21H22N4O2S]+H)+
Counts vs. Mass-to-Charge (m/z)

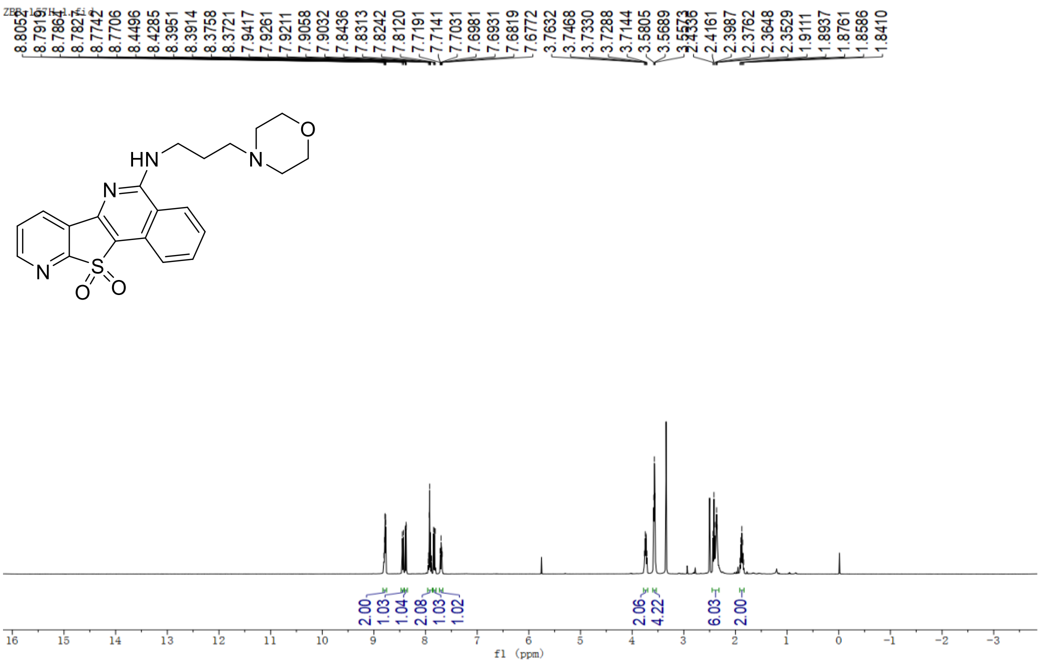
ZBB-157H.1.fid
8.8052
8.7919
8.7863
8.7821
8.7742
8.7706
8.4496
8.4285
8.3951
8.3914
8.3758
8.3721
7.9417
7.9261
7.9211
7.9058
7.9032
7.8436
7.8313
7.8242
7.8120
7.7191
7.7141
7.7031
7.6981
7.6931
7.6819
7.6772
3.7632
3.7468
3.7330
3.7288
3.7144
3.5805
3.5689
3.5573
2.4336
2.4161
2.3987
2.3762
2.3648
2.3529
1.9111
1.8937
1.8761
1.8586
1.8410
HN
N
O
N
N
S
O
O
2.00
1.03
1.04
2.08
1.03
1.02
2.06
4.22
6.03
2.00
16 15 14 13 12 11 10 9 8 7 6 5 4 3 2 1 0 -1 -2 -3
f1 (ppm)

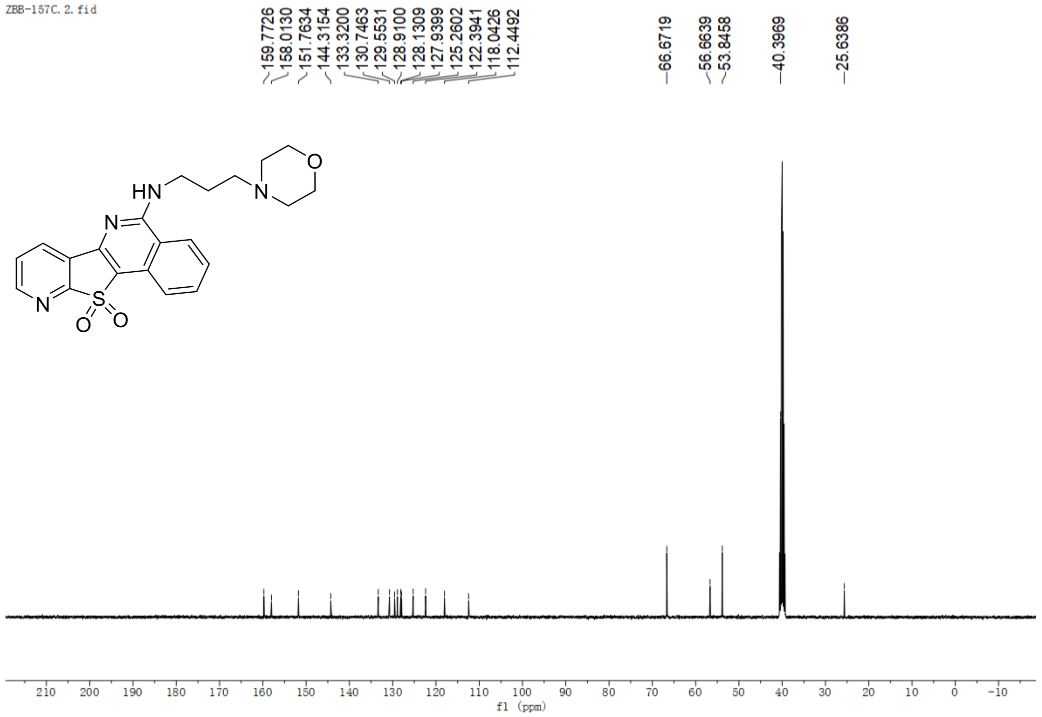
ZBB-157C.2.fid
159.7726
158.0130
151.7634
144.3154
133.3200
130.7463
129.5531
128.9100
128.1309
127.9399
125.2602
122.3941
118.0426
112.4492
66.6719
56.6639
53.8458
40.3969
25.6386
HN
N
O
N
N
S
O
O
210 200 190 180 170 160 150 140 130 120 110 100 90 80 70 60 50 40 30 20 10 0 -10
f1 (ppm)

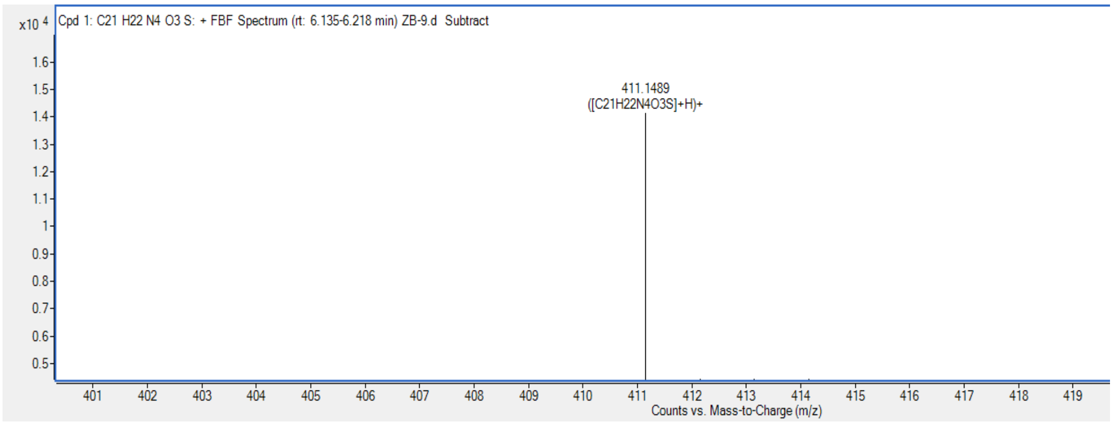
x10 4
Cpd 1: C21 H22 N4 O3 S: + FBF Spectrum (rt: 6.135-6.218 min) ZB-9.d Subtract
411.1489
([C21H22N4O3S]+H)+
1.6 1.5 1.4 1.3 1.2 1.1 1 0.9 0.8 0.7 0.6 0.5
401 402 403 404 405 406 407 408 409 410 411 412 413 414 415 416 417 418 419
Counts vs. Mass-to-Charge (m/z)

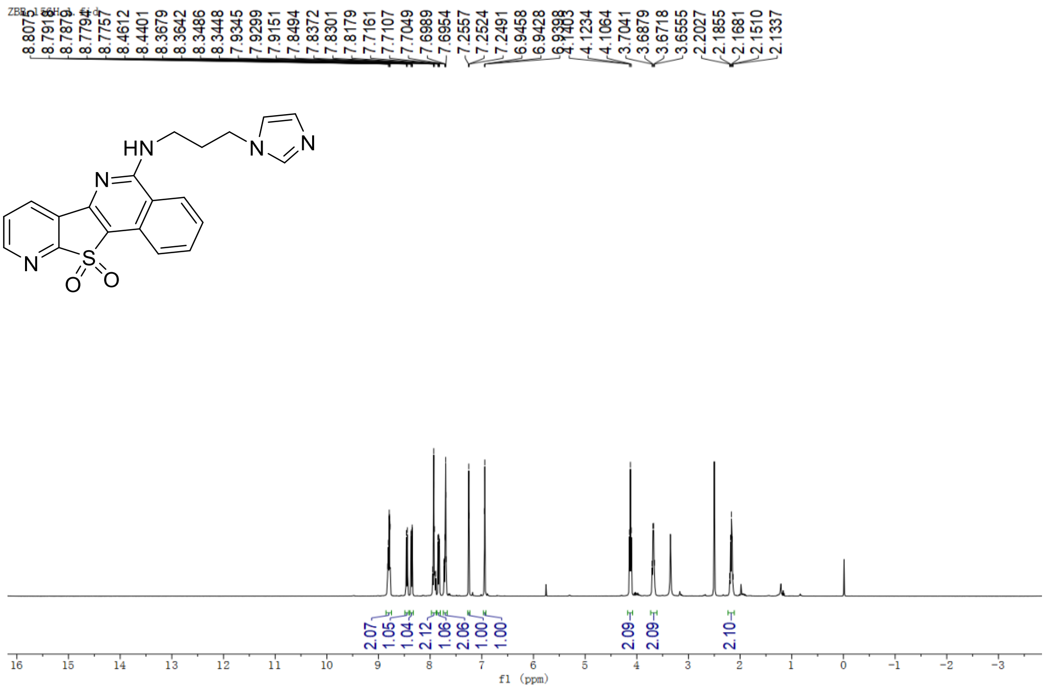
8.8075
8.7916
8.7879
8.7794
8.7757
8.4612
8.4401
8.3679
8.3642
8.3486
8.3448
7.9345
7.9299
7.9151
7.8494
7.8372
7.8301
7.8179
7.7161
7.7107
7.7049
7.6989
7.6954
7.2557
7.2524
7.2491
6.9458
6.9428
4.1234
4.1064
3.7041
3.6879
3.6718
3.6555
2.2027
2.1855
2.1681
2.1510
2.1337
HN
N
N
N
N
S
O
O
2.07
1.05
1.04
2.12
1.06
2.06
1.00
1.00
2.09
2.09
2.10
f1 (ppm)

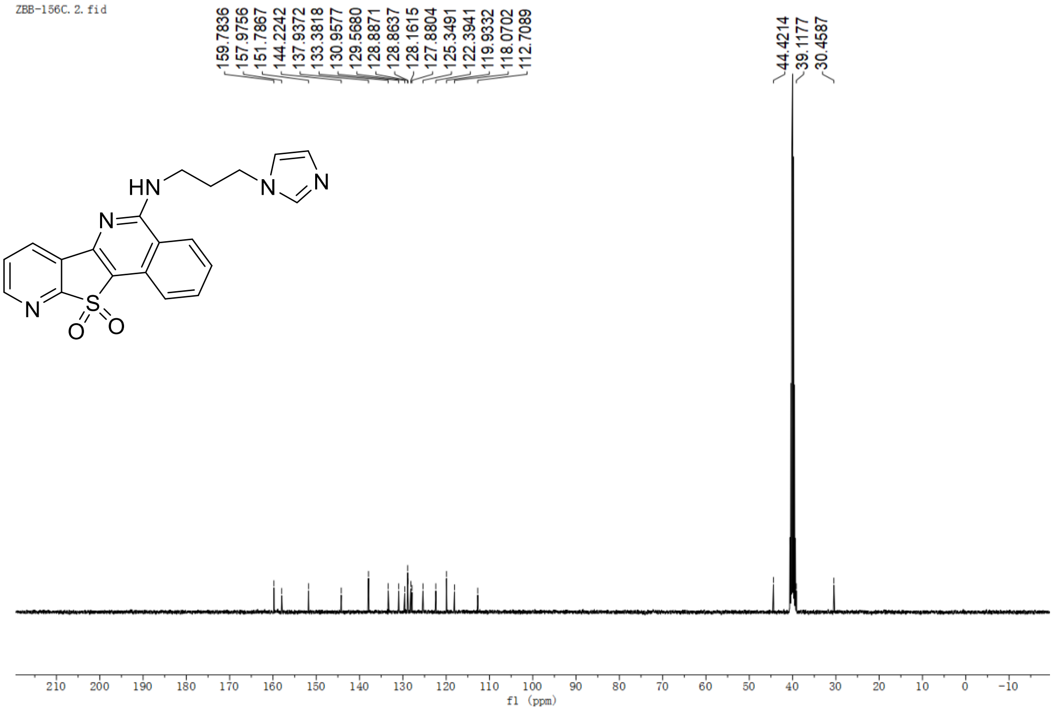
ZBB-156C.2.fid
159.7836
157.9756
151.7867
144.2242
137.9372
133.3818
130.9577
129.5680
128.8871
128.8637
128.1615
127.8804
125.3491
122.3941
119.9332
118.0702
112.7089
44.4214
39.1177
30.4587
HN
N
N
N
N
S
O
O
f1 (ppm)

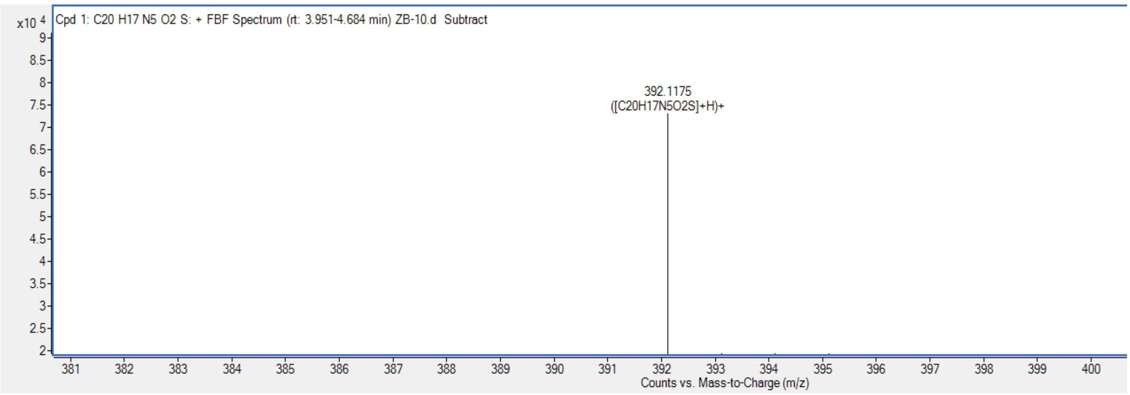
x10 4
Cpd 1: C20 H17 N5 O2 S: + FBF Spectrum (rt: 3.951-4.684 min) ZB-10.d Subtract
392.1175
([C20H17N5O2S]+H)+
Counts vs. Mass-to-Charge (m/z)

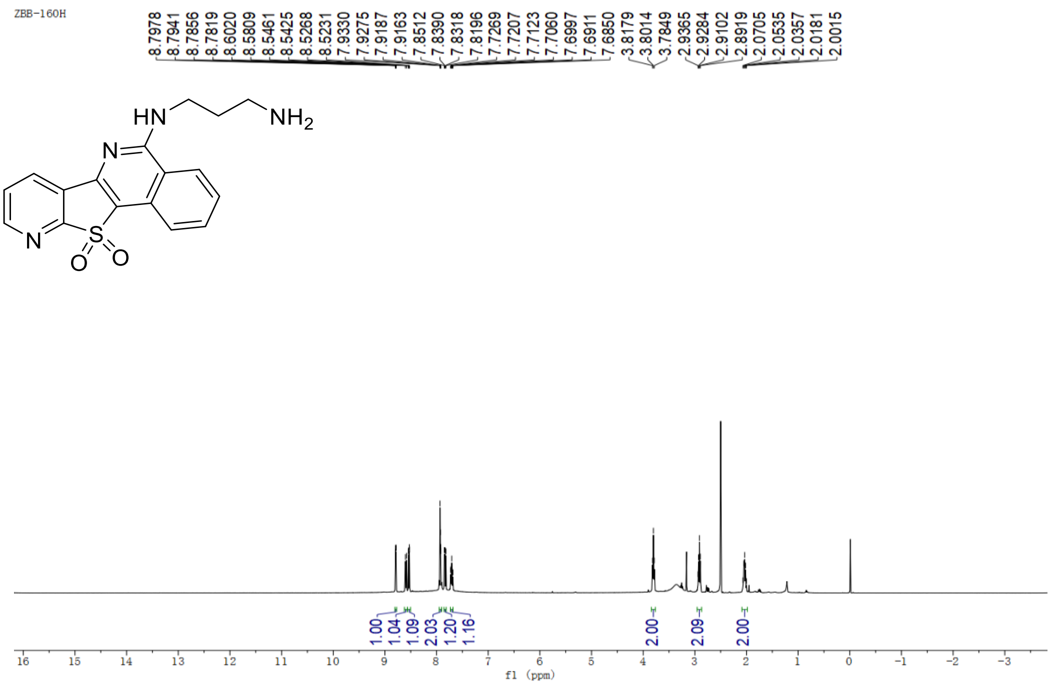
ZBB-160H
8.7978
8.7941
8.7856
8.7819
8.6020
8.5809
8.5461
8.5425
8.5268
8.5231
7.9330
7.9275
7.9187
7.9163
7.8512
7.8390
7.8318
7.8196
7.7269
7.7207
7.7123
7.7060
7.6997
7.6911
7.6850
3.8179
3.8014
3.7849
2.9365
2.9284
2.9102
2.8919
2.0705
2.0535
2.0357
2.0181
2.0015
HN
NH2
N
N
S
O
O
1.00
1.04
1.09
2.03
1.20
1.16
2.00
2.09
2.00
f1 (ppm)

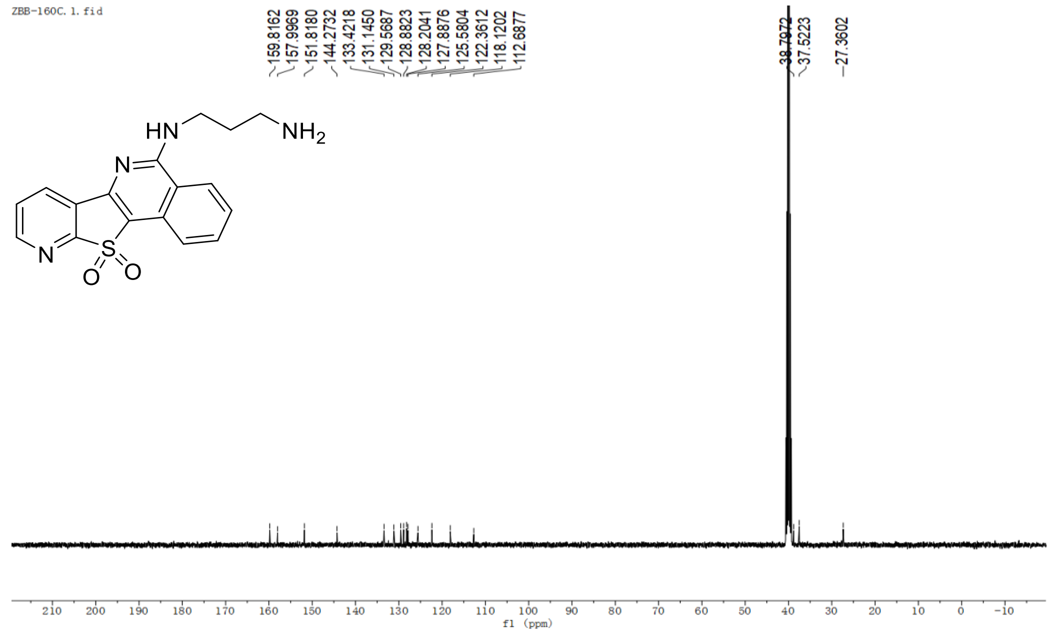
ZBB-160C.1.fid
159.8162
157.9969
151.8180
144.2732
133.4218
131.1450
129.5687
128.8823
128.2041
127.8876
125.5804
122.3612
118.1202
112.6877
38.7972
37.5223
27.3602
HN
NH2
N
N
S
O
O
f1 (ppm)

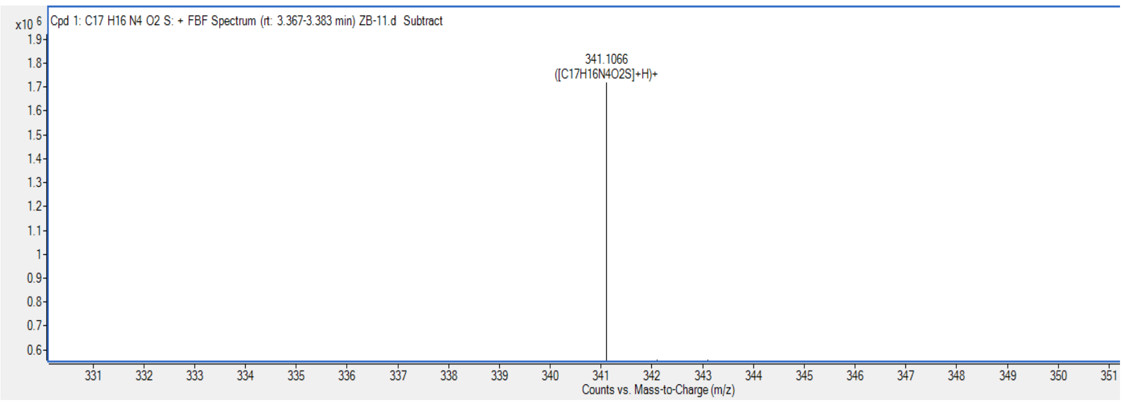
Cpd 1: C17 H16 N4 O2 S: + FBF Spectrum (rt: 3.367-3.383 min) ZB-11.d Subtract
x10 6
341.1066
([C17H16N4O2S]+H)+
Counts vs. Mass-to-Charge (m/z)

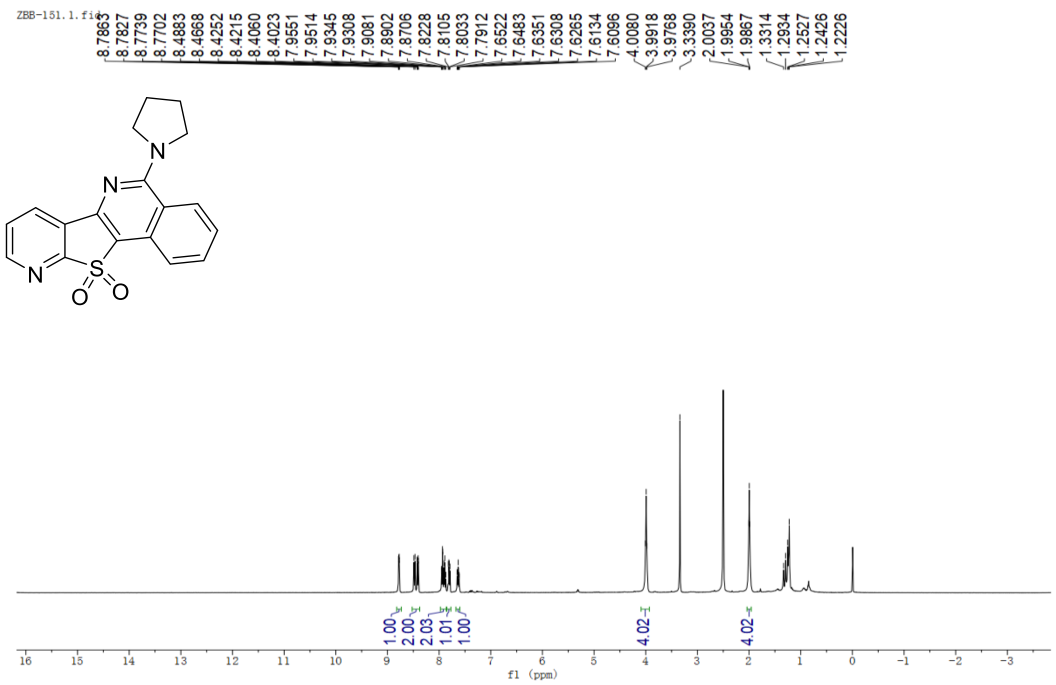
ZBB-151.1.fid
8.7863
8.7827
8.7739
8.7702
8.4883
8.4668
8.4252
8.4215
8.4060
8.4023
7.9551
7.9514
7.9345
7.9308
7.9081
7.8902
7.8706
7.8228
7.8105
7.8033
7.7912
7.6522
7.6483
7.6351
7.6308
7.6265
7.6134
7.6096
4.0080
3.9918
3.9768
3.3390
2.0037
1.9954
1.9867
1.3314
1.2934
1.2527
1.2426
1.2226
N
N
N
S
O
O
1.00
2.00
2.03
1.01
1.00
4.02
4.02
f1 (ppm)

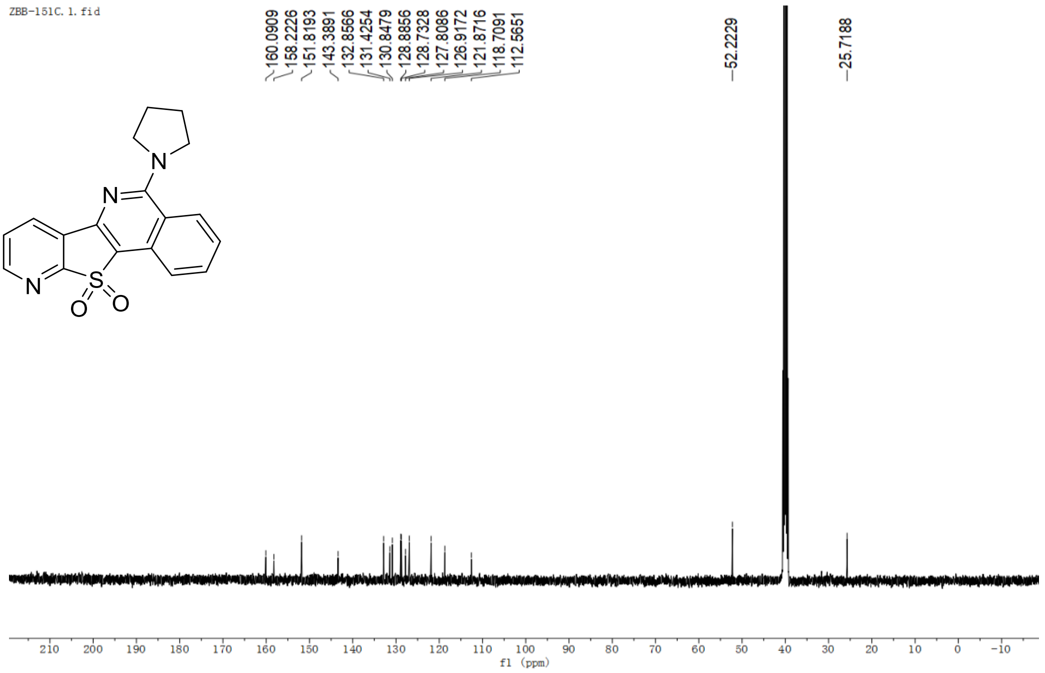
ZBB-151C.1.fid
160.0909
158.2226
151.8193
143.3891
132.8566
131.4254
130.8479
128.8856
128.7328
127.8086
126.9172
121.8716
118.7091
112.5651
52.2229
25.7188
N
N
N
S
O
O
f1 (ppm)

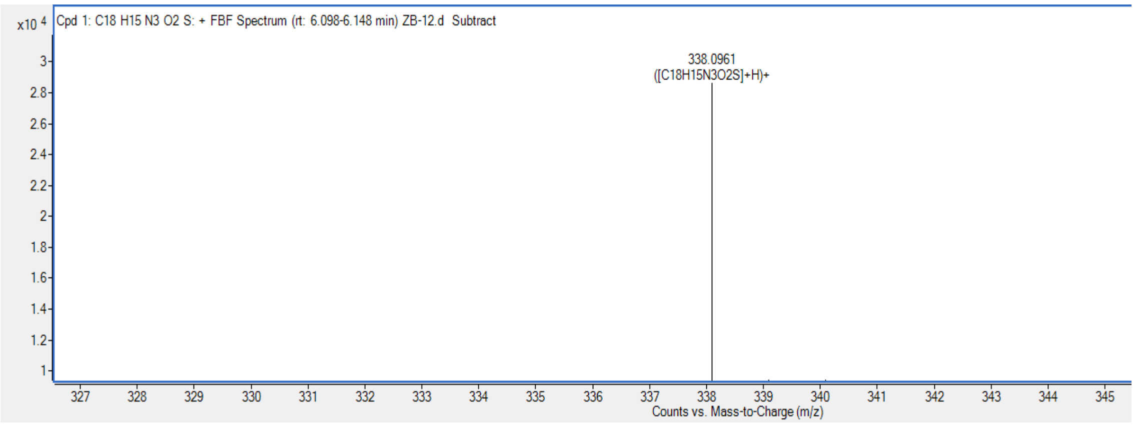
x10 4
Cpd 1: C18 H15 N3 O2 S: + FBF Spectrum (rt: 6.098-6.148 min) ZB-12.d Subtract
338.0961
([C18H15N3O2S]+H)+
Counts vs. Mass-to-Charge (m/z)

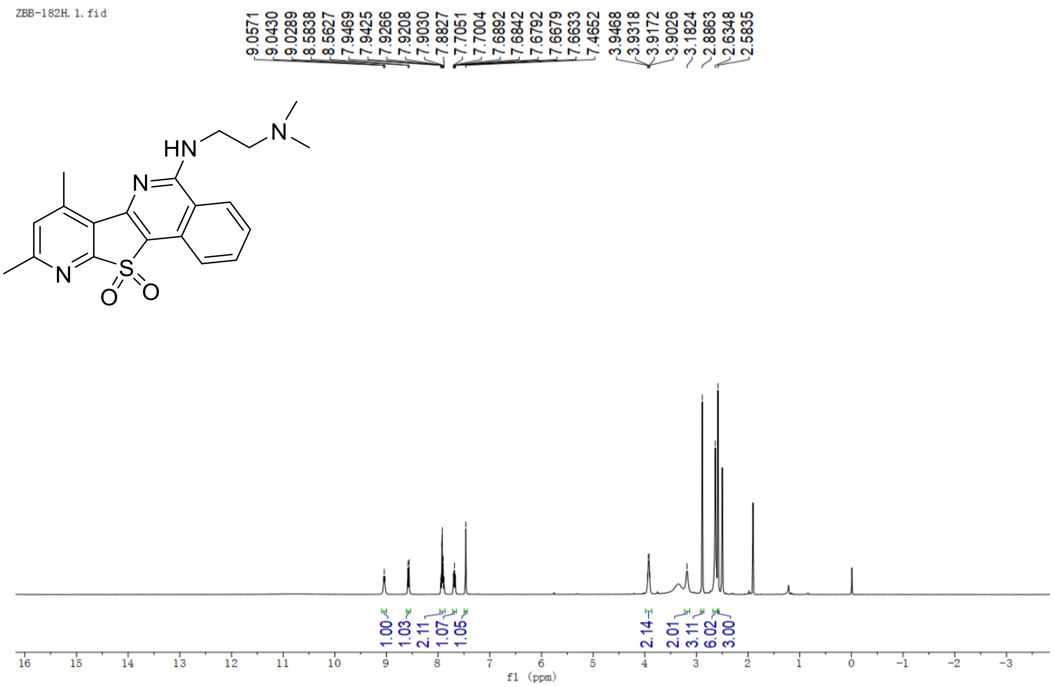
ZBB-182H.1.fid
9.0571
9.0430
9.0289
8.5838
8.5627
7.9469
7.9425
7.9266
7.9208
7.9030
7.8827
7.7051
7.7004
7.6892
7.6842
7.6792
7.6679
7.6633
7.4652
3.9468
3.9318
3.9172
3.9026
3.1824
2.8863
2.6348
2.5835
HN
N
N
N
S
O
O
1.00
1.03
2.11
1.07
1.05
2.14
2.01
3.11
6.02
3.00
f1 (ppm)

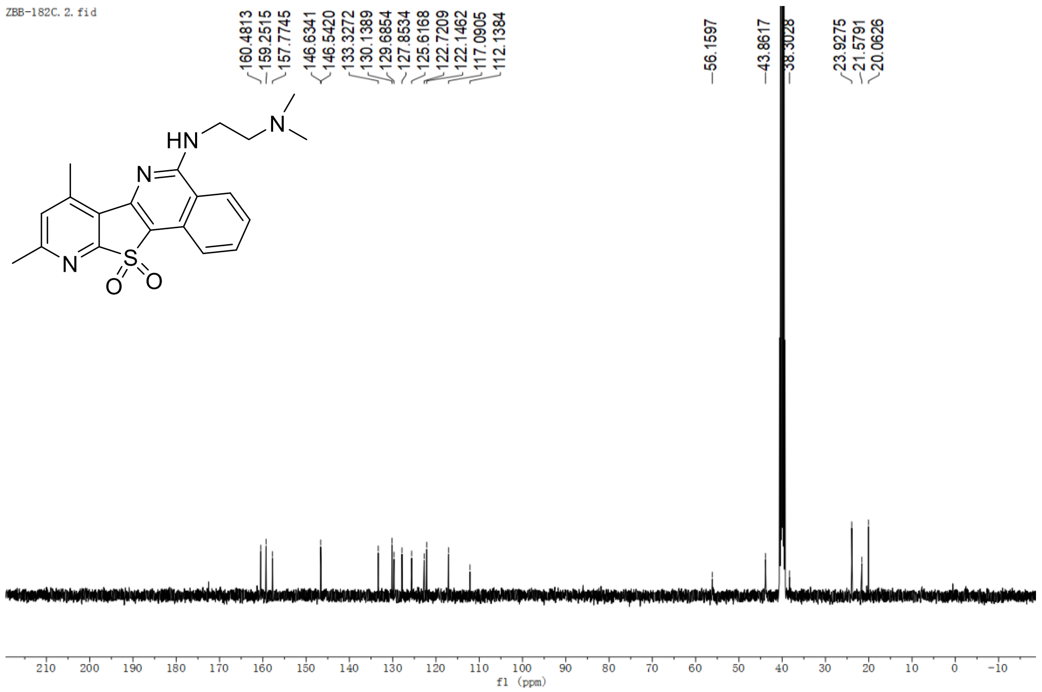
ZBB-182C.2.fid
160.4813
159.2515
157.7745
146.6341
146.5420
133.3272
130.1389
129.6854
127.8534
125.6168
122.7209
122.1462
117.0905
112.1384
56.1597
43.8617
38.3028
23.9275
21.5791
20.0626
HN
N
N
N
S
O
O
f1 (ppm)

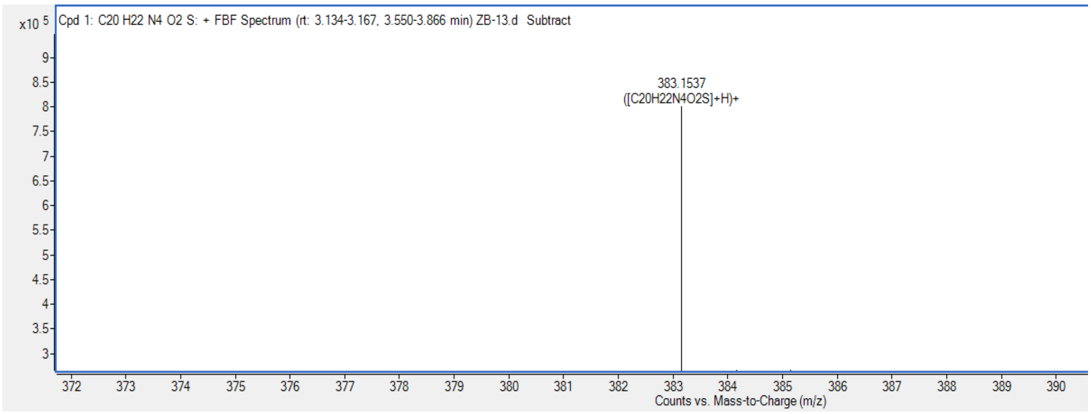
x10 5
Cpd 1: C20 H22 N4 O2 S: + FBF Spectrum (rt: 3.134-3.167, 3.550-3.866 min) ZB-13.d Subtract
383.1537
([C20H22N4O2S]+H)+
Counts vs. Mass-to-Charge (m/z)

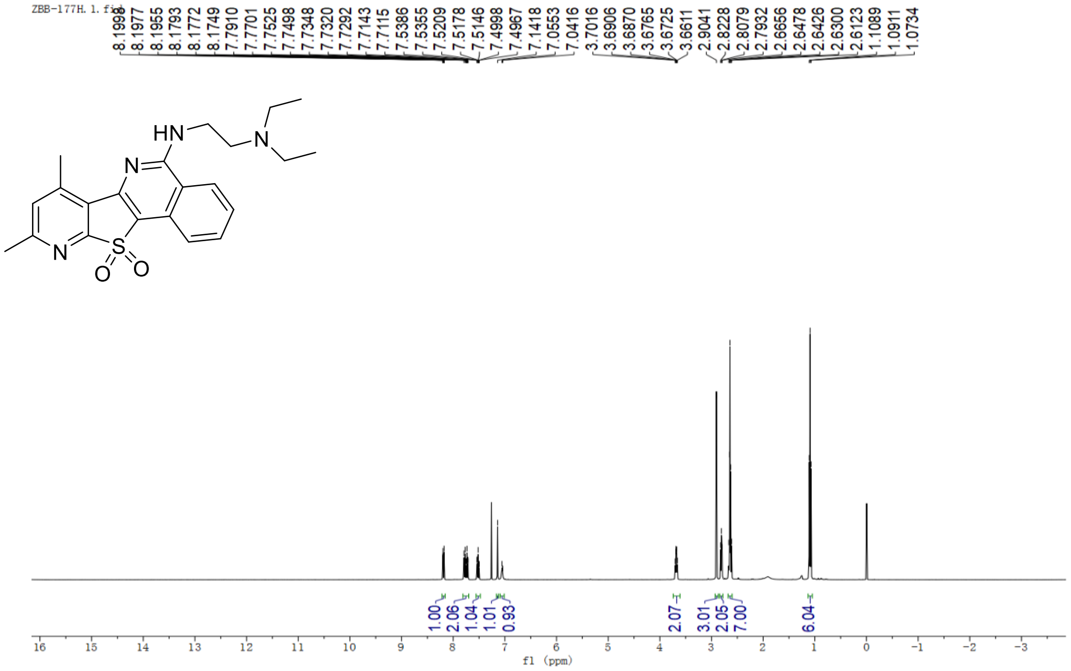
ZBB-177H.1.fid
8.1998
8.1977
8.1955
8.1793
8.1772
8.1749
7.7910
7.7701
7.7525
7.7498
7.7348
7.7320
7.7292
7.7143
7.7115
7.5386
7.5355
7.5209
7.5178
7.5146
7.4998
7.4967
7.1418
7.0553
7.0416
3.7016
3.6906
3.6870
3.6765
3.6725
3.6611
2.9041
2.8228
2.8079
2.7932
2.6656
2.6478
2.6426
2.6300
2.6123
1.1089
1.0911
1.0734
HN
N
N
S
O
O
1.00
2.06
1.04
1.01
0.93
2.07
3.01
2.05
7.00
6.04
f1 (ppm)

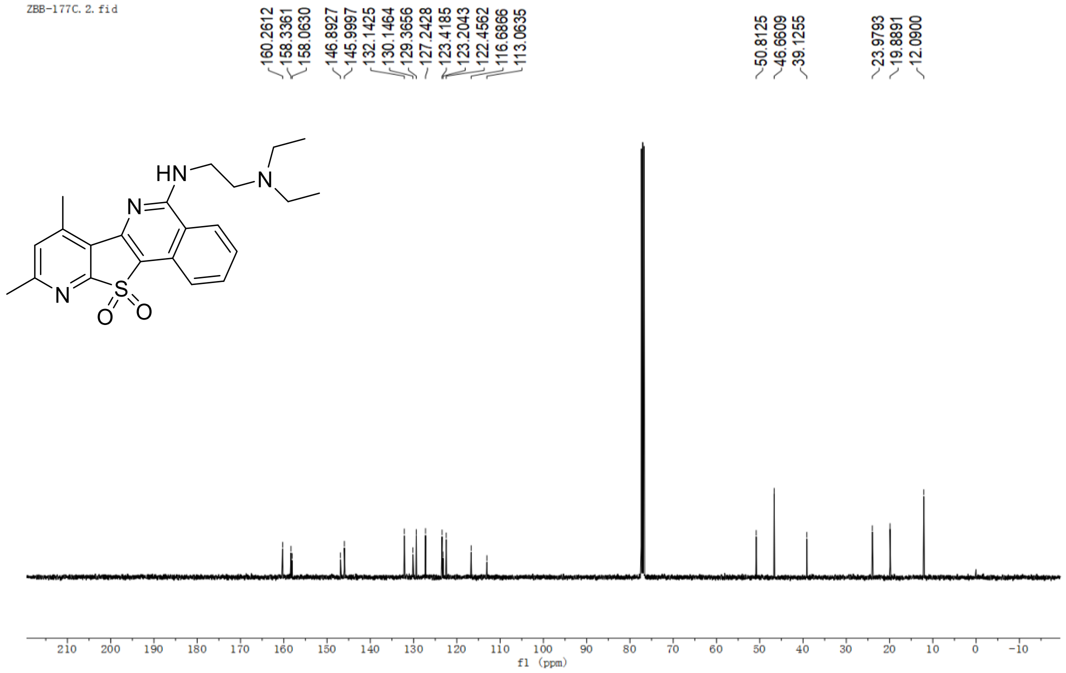
ZBB-177C.2.fid
160.2612
158.3361
158.0630
146.8927
145.9997
132.1425
130.1464
129.3656
127.2428
123.4185
123.2043
122.4562
116.6866
113.0635
50.8125
46.6609
39.1255
23.9793
19.8891
12.0900
HN
N
N
S
O
O
f1 (ppm)

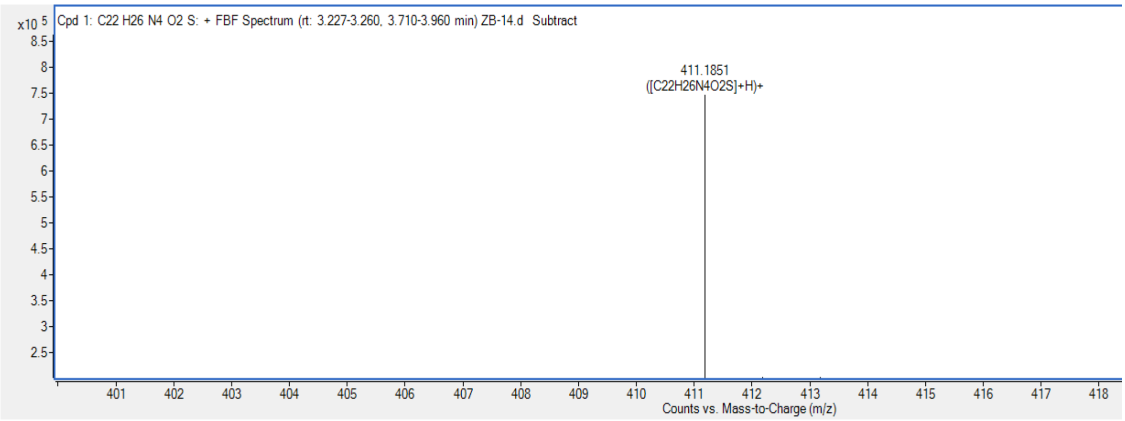
x10 5
Cpd 1: C22 H26 N4 O2 S: + FBF Spectrum (rt: 3.227-3.260, 3.710-3.960 min) ZB-14.d Subtract
411.1851
([C22H26N4O2S]+H)+
Counts vs. Mass-to-Charge (m/z)

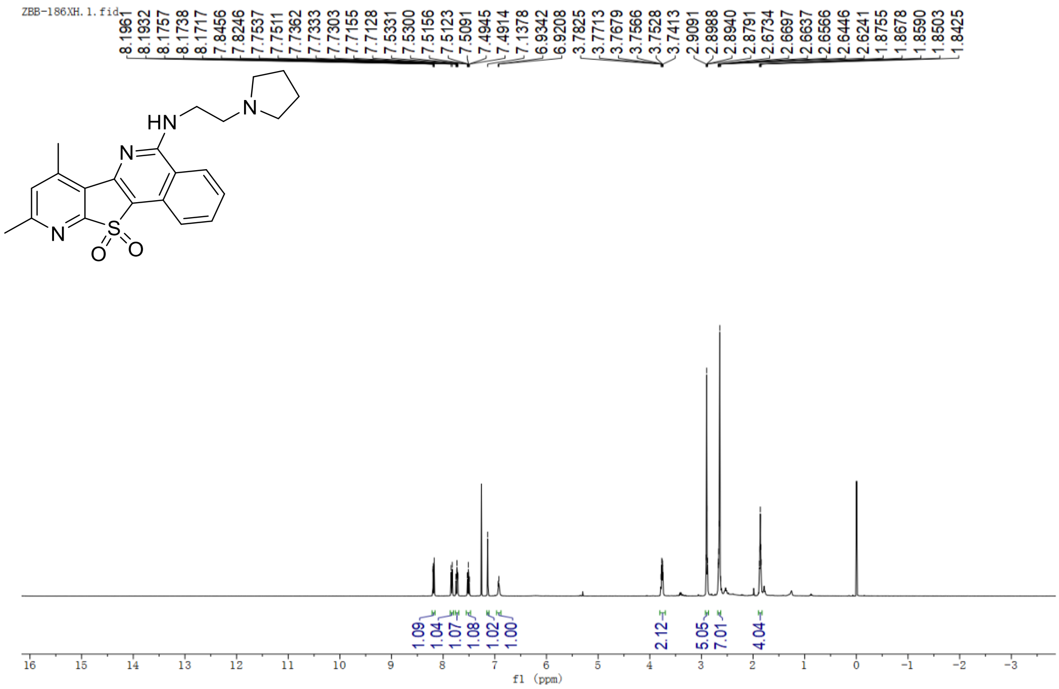
ZBB-186XH.1.fid
8.1961
8.1932
8.1757
8.1738
8.1717
7.8456
7.8246
7.7537
7.7511
7.7362
7.7333
7.7303
7.7155
7.7128
7.5331
7.5300
7.5156
7.5123
7.5091
7.4945
7.4914
7.1378
6.9342
6.9208
3.7825
3.7713
3.7679
3.7566
3.7528
3.7413
2.9091
2.8988
2.8940
2.8791
2.6734
2.6697
2.6637
2.6566
2.6446
2.6241
1.8755
1.8678
1.8590
1.8503
1.8425
HN
N
N
N
S
O
O
1.09
1.04
1.07
1.08
1.02
1.00
2.12
5.05
7.01
4.04
f1 (ppm)

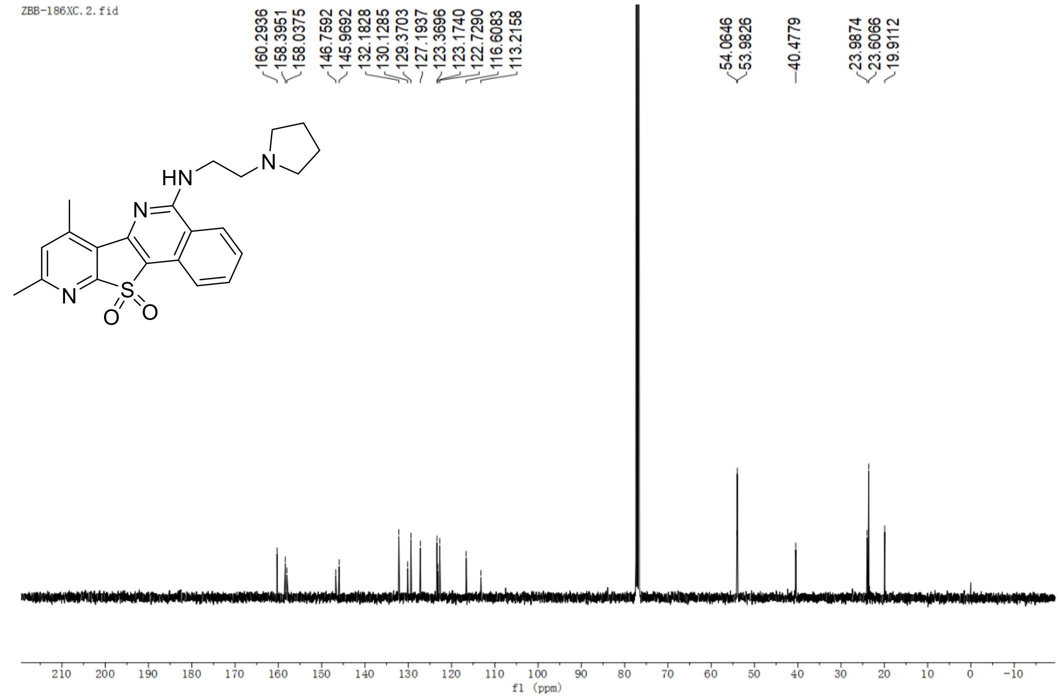
ZBB-186XC.2.fid
160.2936
158.3951
158.0375
146.7592
145.9692
132.1828
130.1285
129.3703
127.1937
123.3696
123.1740
122.7290
116.6083
113.2158
54.0646
53.9826
40.4779
23.9874
23.6066
19.9112
HN
N
N
N
S
O
O
f1 (ppm)

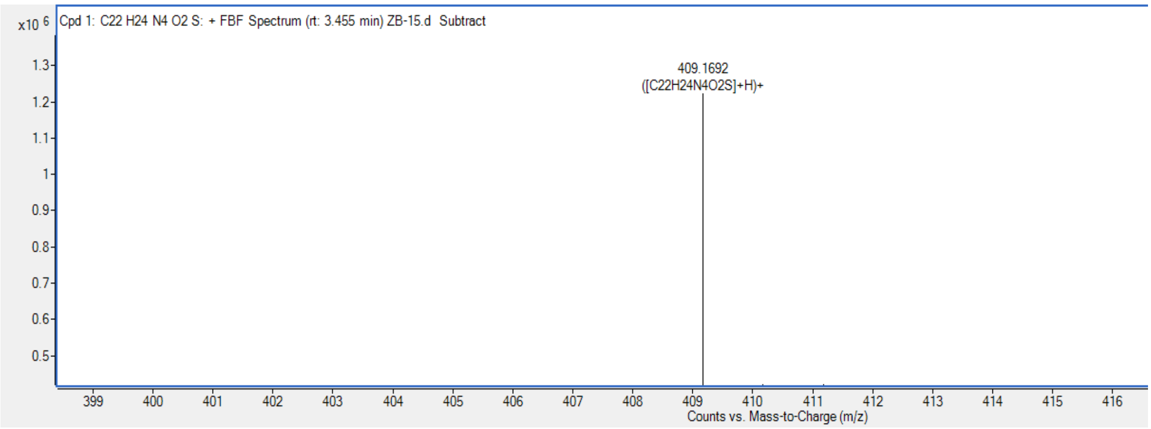
x10 6
Cpd 1: C22 H24 N4 O2 S: + FBF Spectrum (rt: 3.455 min) ZB-15.d Subtract
409.1692
([C22H24N4O2S]+H)+
Counts vs. Mass-to-Charge (m/z)

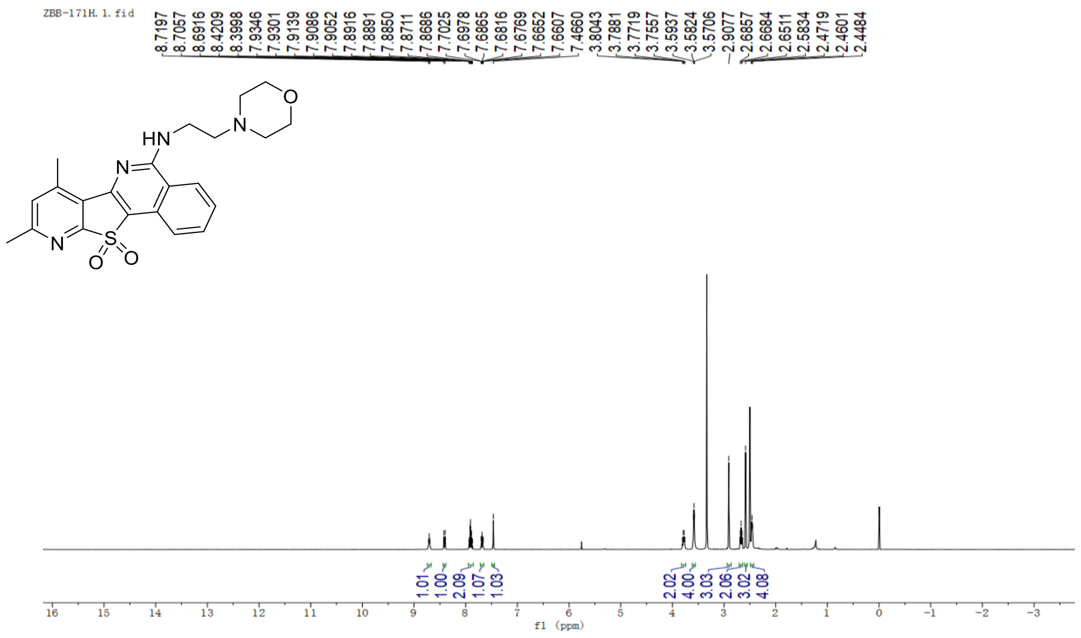
ZBB-171H. 1. fid
8.7197
8.7057
8.6916
8.4209
8.3998
7.9346
7.9301
7.9139
7.9086
7.9052
7.8916
7.8891
7.8850
7.8711
7.8686
7.7025
7.6978
7.6865
7.6816
7.6769
7.6652
7.6607
7.4660
3.8043
3.7881
3.7719
3.7557
3.5937
3.5824
3.5706
2.9077
2.6857
2.6684
2.6511
2.5834
2.4719
2.4601
2.4484
HN
N
N
O
S
O
O
N
1.01
1.00
2.09
1.07
1.03
2.02
4.00
3.03
2.06
3.02
4.08
f1 (ppm)

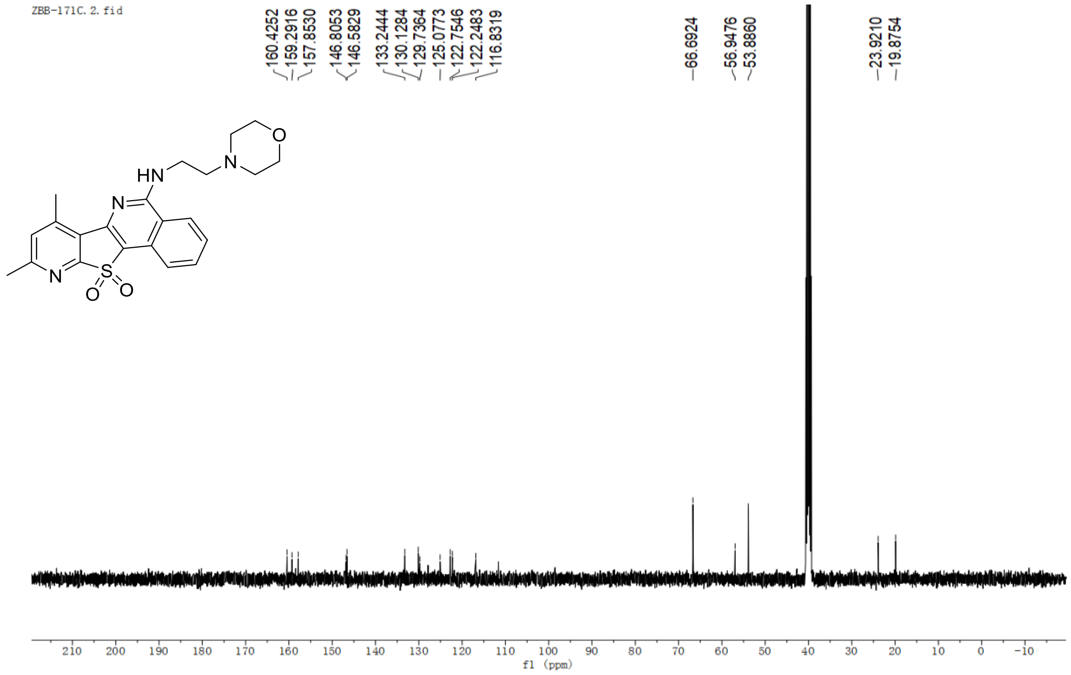
ZBB-171C. 2. fid
160.4252
159.2916
157.8530
146.8053
146.5829
133.2444
130.1284
129.7364
125.0773
122.7546
122.2483
116.8319
66.6924
56.9476
53.8860
23.9210
19.8754
HN
N
N
O
S
O
O
N
f1 (ppm)

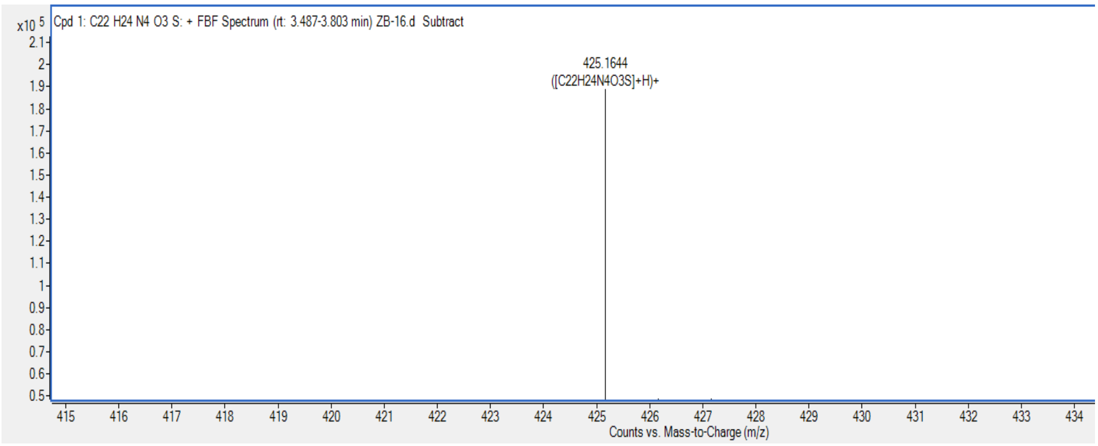
x10 5
Cpd 1: C22 H24 N4 O3 S: + FBF Spectrum (rt: 3.487-3.803 min) ZB-16.d Subtract
425.1644
([C22H24N4O3S]+H)+
Counts vs. Mass-to-Charge (m/z)

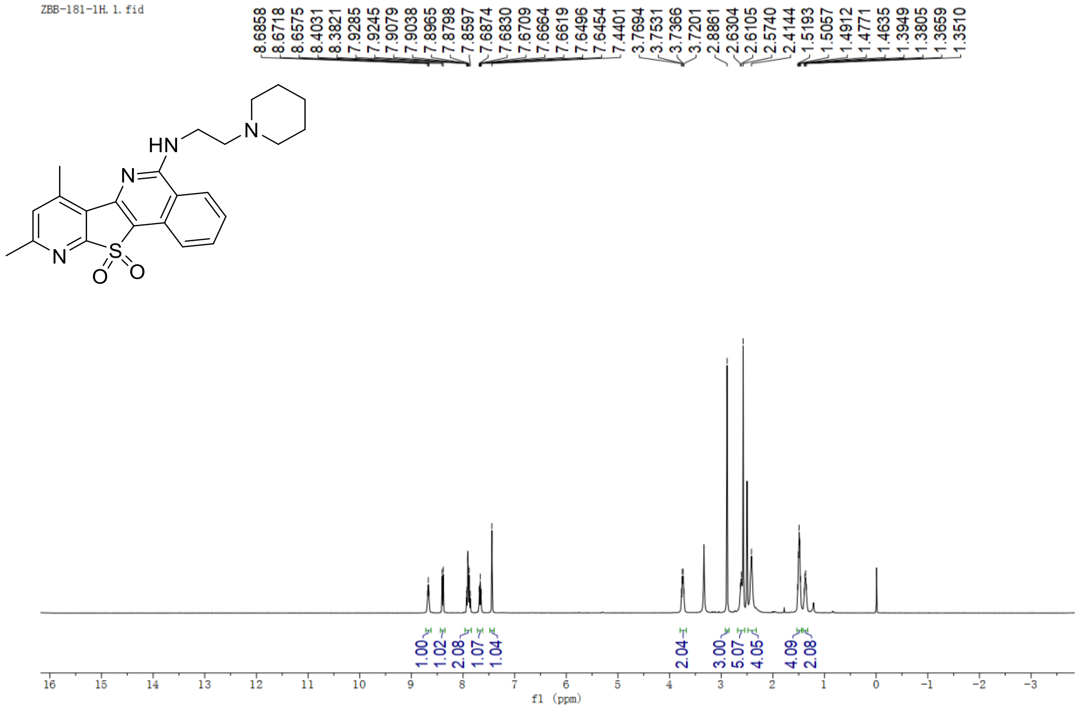
ZBB-181-1H. 1. fid
8.6858
8.6718
8.6575
8.4031
8.3821
7.9285
7.9245
7.9079
7.9038
7.8965
7.8798
7.8597
7.6874
7.6830
7.6709
7.6664
7.6619
7.6496
7.6454
7.4401
3.7694
3.7531
3.7366
3.7201
2.8861
2.6304
2.6105
2.5740
2.4144
1.5193
1.5057
1.4912
1.4771
1.4635
1.3949
1.3805
1.3659
1.3510
1.00
1.02
2.08
1.07
1.04
2.04
3.00
5.07
4.05
4.09
2.08
f1 (ppm)

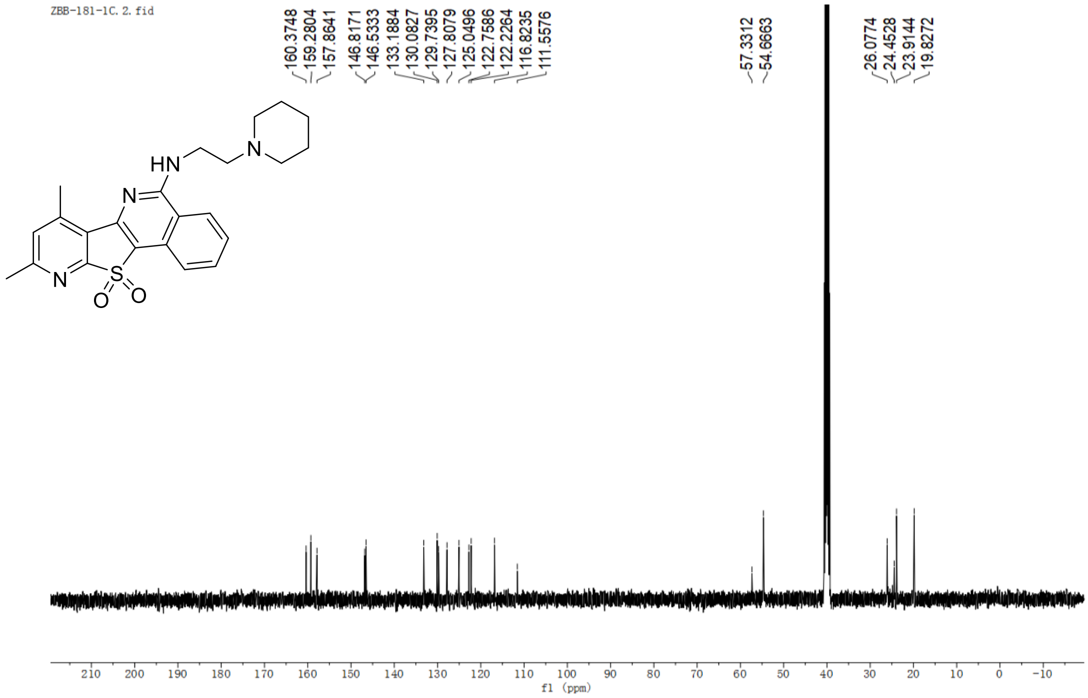
ZBB-181-1C. 2. fid
160.3748
159.2804
157.8641
146.8171
146.5333
133.1884
130.0827
129.7395
127.8079
125.0496
122.7586
122.2264
116.8235
111.5576
57.3312
54.6663
26.0774
24.4528
23.9144
19.8272
f1 (ppm)

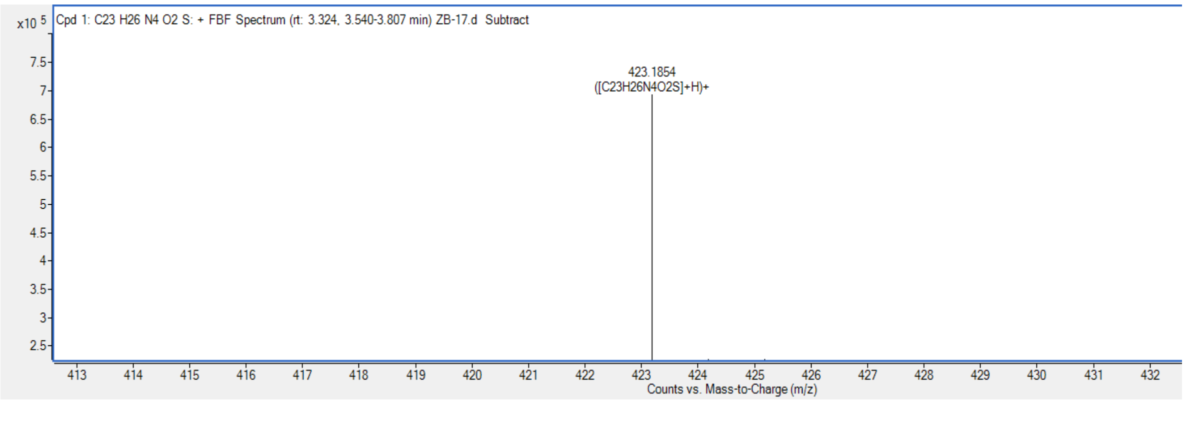
x10 5
Cpd 1: C23 H26 N4 O2 S: + FBF Spectrum (rt: 3.324, 3.540-3.807 min) ZB-17.d Subtract
423.1854
([C23H26N4O2S]+H)+
Counts vs. Mass-to-Charge (m/z)

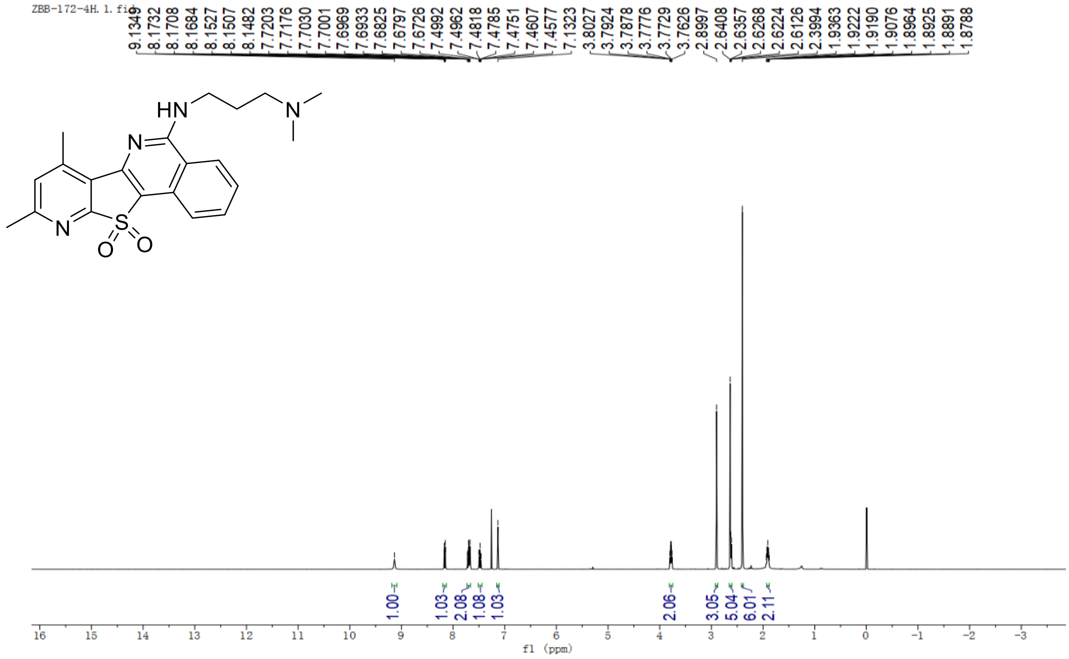
ZBB-172-4H. 1. fid
9.1349
8.1732
8.1708
8.1684
8.1527
8.1507
8.1482
7.7203
7.7176
7.7030
7.7001
7.6969
7.6933
7.6825
7.6797
7.6726
7.4992
7.4962
7.4818
7.4785
7.4751
7.4607
7.4577
7.1323
3.8027
3.7924
3.7878
3.7776
3.7729
3.7626
2.8997
2.6408
2.6357
2.6268
2.6224
2.6126
2.3894
1.9363
1.9222
1.9190
1.9076
1.8964
1.8925
1.8891
1.8788
HN
N
N
N
S
O
O
1.00
1.03
2.08
1.08
1.03
2.06
3.05
5.04
6.01
2.11
f1 (ppm)

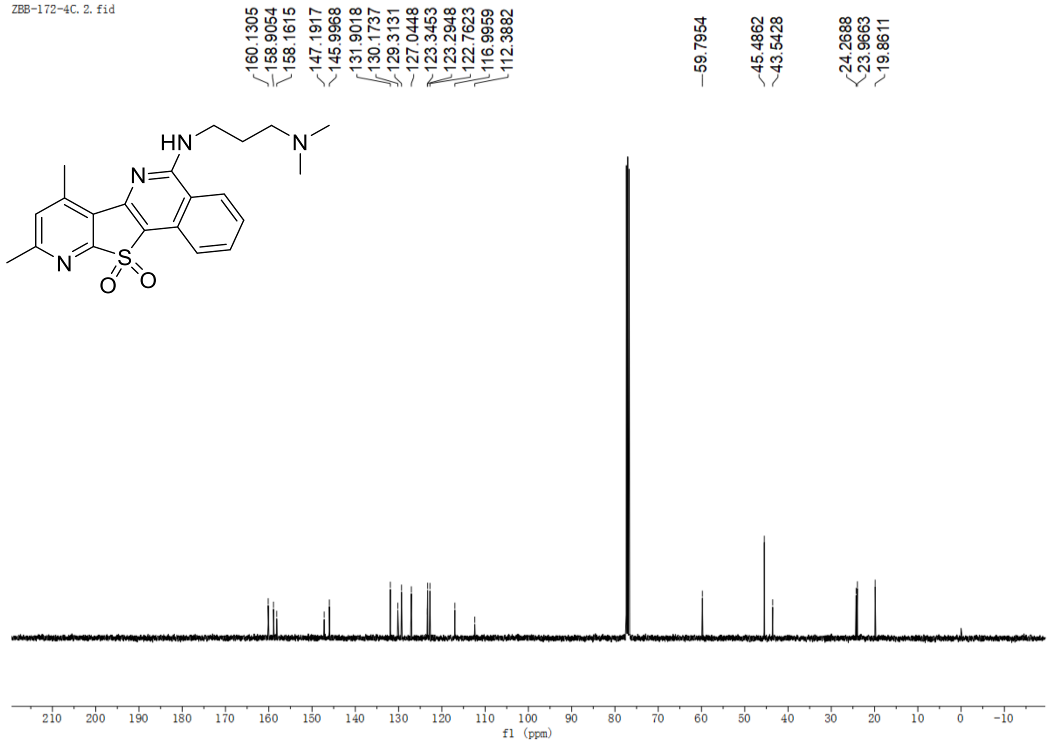
ZBB-172-4C. 2. fid
160.1305
158.9054
158.1615
147.1917
145.9968
131.9018
130.1737
129.3131
127.0448
123.3453
123.2948
122.7623
116.9959
112.3882
59.7954
45.4862
43.5428
24.2688
23.9663
19.8611
HN
N
N
N
S
O
O
f1 (ppm)

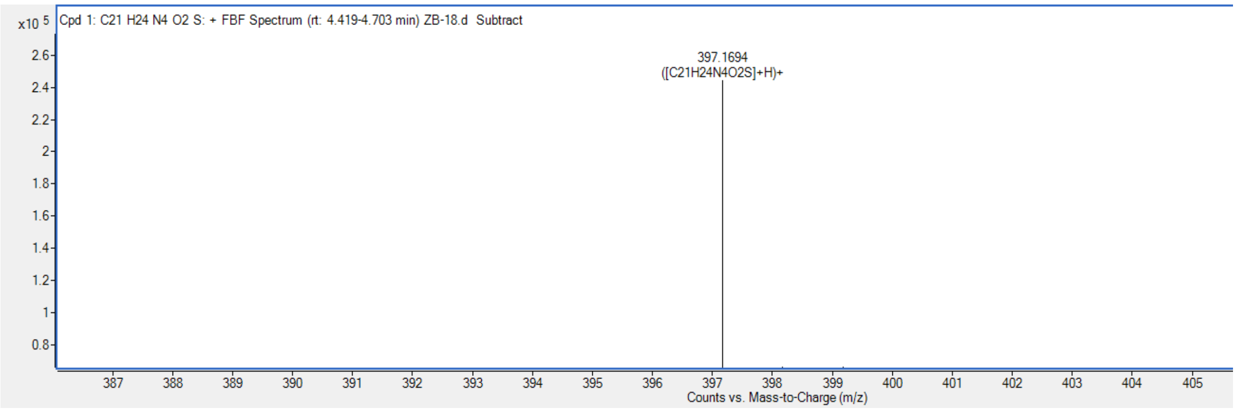
x10 5
Cpd 1: C21 H24 N4 O2 S: + FBF Spectrum (rt: 4.419-4.703 min) ZB-18.d Subtract
397.1694
([C21H24N4O2S]+H)+
Counts vs. Mass-to-Charge (m/z)

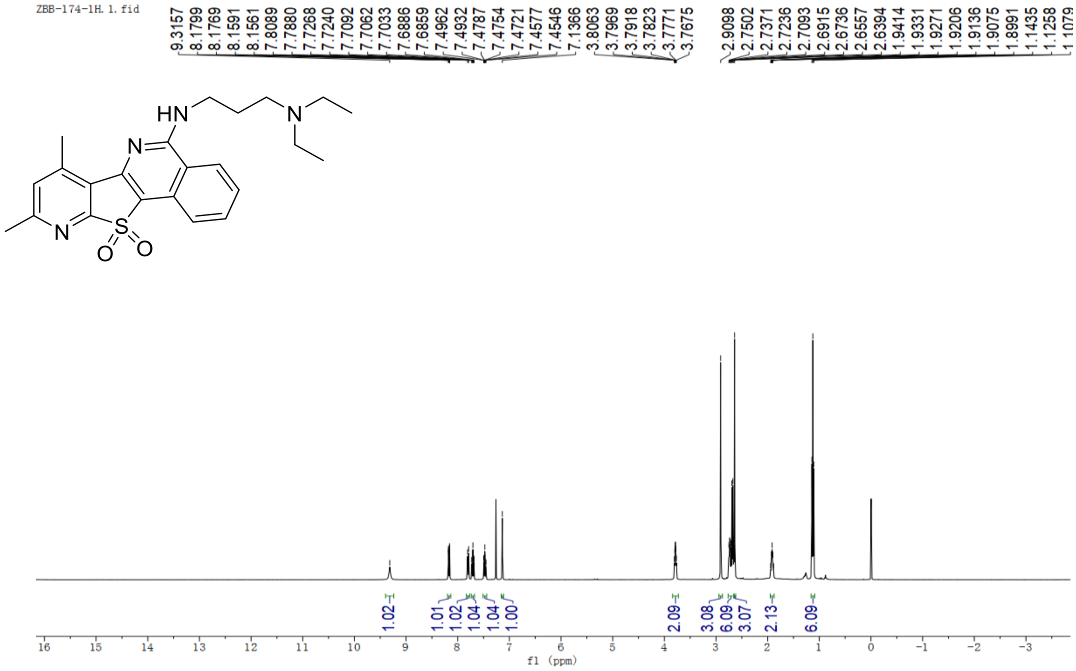
ZBB-174-1H. 1. fid
9.3157
8.1799
8.1769
8.1591
8.1561
7.8089
7.7880
7.7268
7.7240
7.7092
7.7062
7.7033
7.6886
7.6859
7.4962
7.4932
7.4787
7.4754
7.4721
7.4577
7.4546
7.1366
3.8063
3.7969
3.7918
3.7823
3.7771
3.7675
2.9098
2.7502
2.7371
2.7236
2.7093
2.6915
2.6736
2.6557
2.6394
1.9414
1.9331
1.9271
1.9206
1.9136
1.9075
1.8991
1.1435
1.1258
1.1079
HN
N
N
N
S
O
O
1.02
1.01
1.02
1.04
1.04
1.00
2.09
3.08
6.09
3.07
2.13
6.09
f1 (ppm)

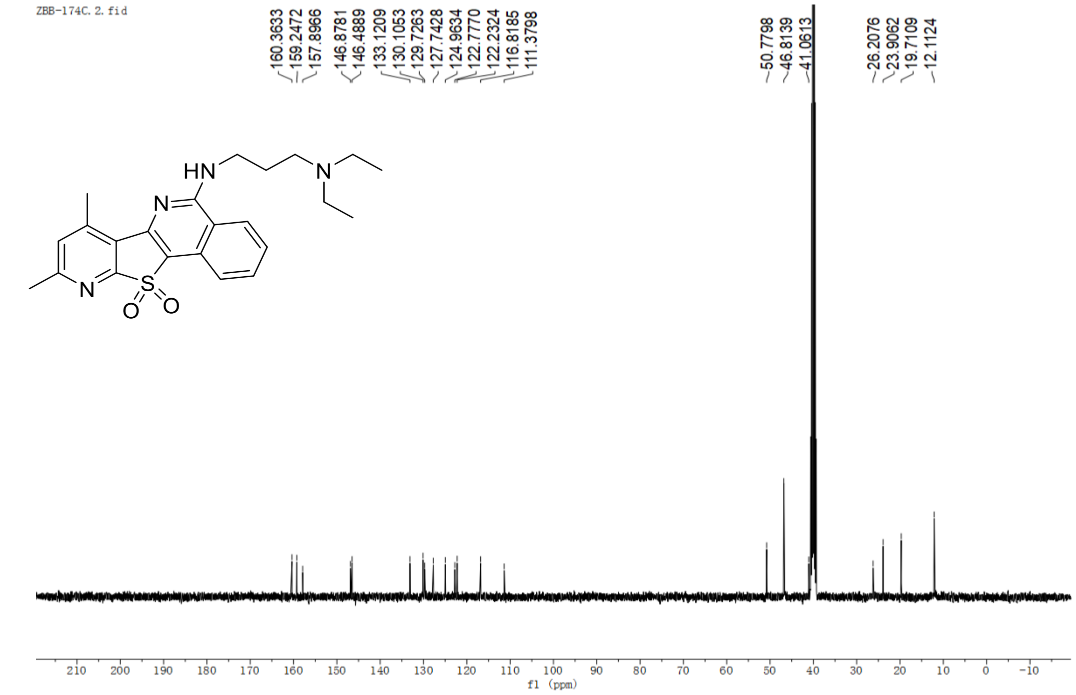
ZBB-174C. 2. fid
160.3633
159.2472
157.8966
146.8781
146.4889
133.1209
130.1053
129.7263
127.7428
124.9634
122.7770
122.2324
116.8185
111.3798
50.7798
46.8139
41.0613
26.2076
23.9062
19.7109
12.1124
HN
N
N
N
S
O
O
f1 (ppm)

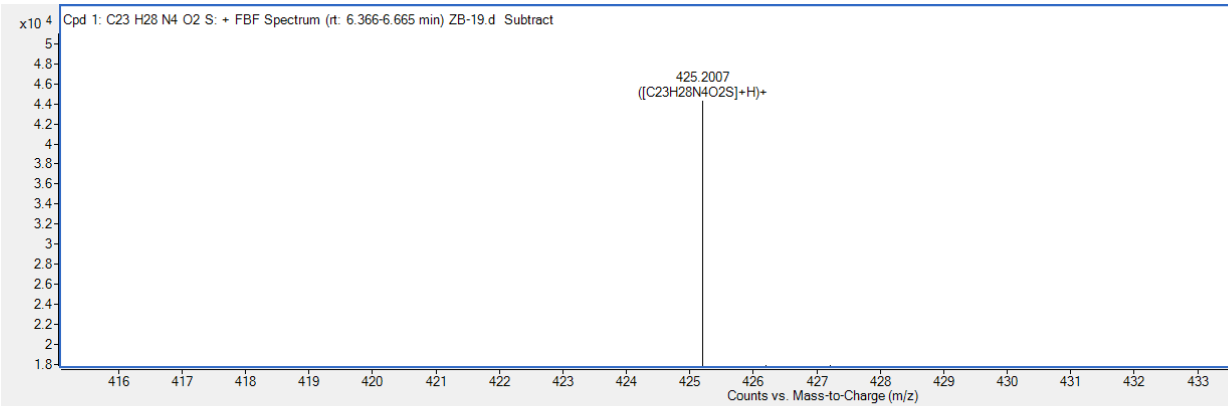
x10 4
Cpd 1: C23 H28 N4 O2 S: + FBF Spectrum (rt: 6.366-6.665 min) ZB-19.d Subtract
425.2007
([C23H28N4O2S]+H)+
Counts vs. Mass-to-Charge (m/z)

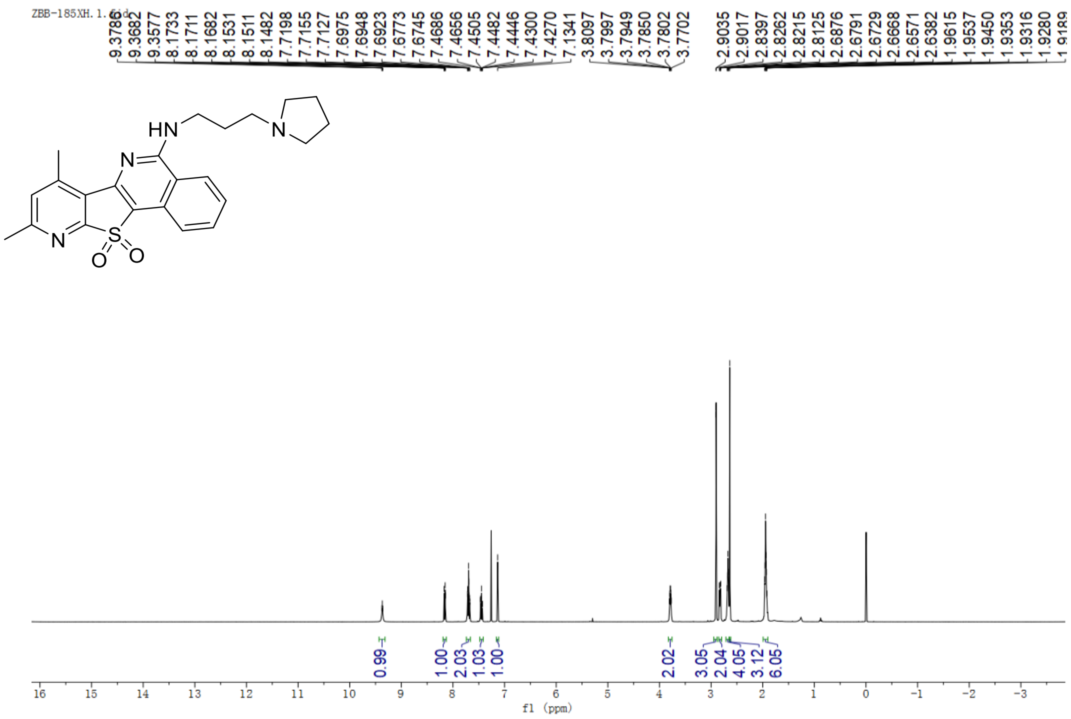
ZBB-185XH.1.fid
9.3786
9.3682
9.3577
8.1733
8.1711
8.1682
8.1531
8.1511
8.1482
7.7198
7.7155
7.7127
7.6975
7.6948
7.6923
7.6773
7.6745
7.4686
7.4656
7.4505
7.4482
7.4446
7.4300
7.4270
7.1341
3.8097
3.7997
3.7949
3.7850
3.7802
3.7702
2.9035
2.9017
2.8397
2.8262
2.8215
2.8125
2.6876
2.6791
2.6729
2.6668
2.6571
2.6382
1.9615
1.9537
1.9450
1.9353
1.9316
1.9280
1.9189
HN
N
N
N
S
O
O
0.99
1.00
2.03
1.03
1.00
2.02
3.05
2.04
4.05
3.12
6.05
f1 (ppm)

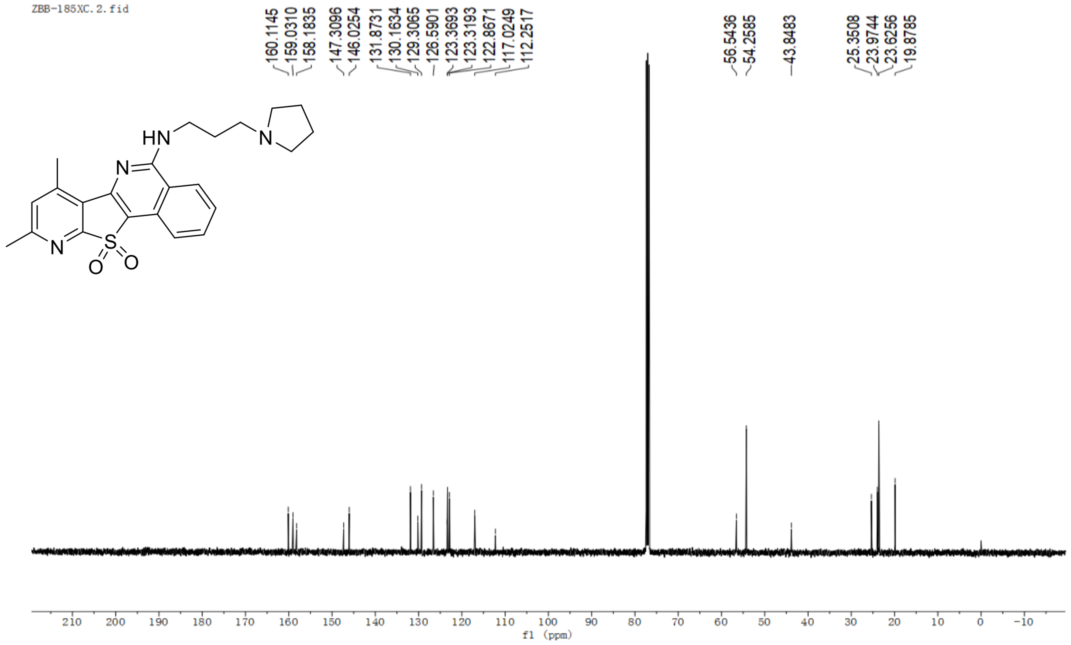
ZBB-185XC.2.fid
160.1145
159.0310
158.1835
147.3096
146.0254
131.8731
130.1634
129.3065
126.5901
123.3693
123.3193
122.8671
117.0249
112.2517
56.5436
54.2585
43.8483
25.3508
23.9744
23.6256
19.8785
HN
N
N
N
S
O
O
f1 (ppm)

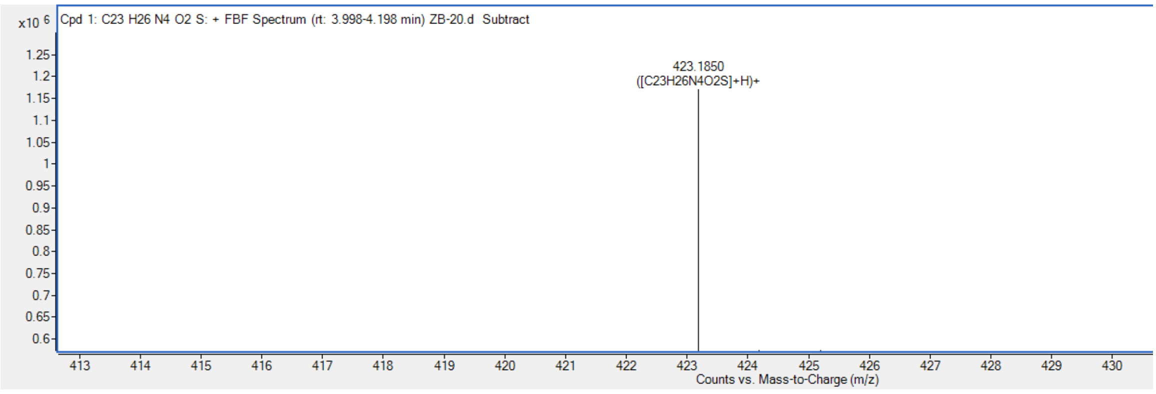
Cpd 1: C23 H26 N4 O2 S: + FBF Spectrum (rt: 3.998-4.198 min) ZB-20.d Subtract
x10 6
423.1850
([C23H26N4O2S]+H)+
Counts vs. Mass-to-Charge (m/z)

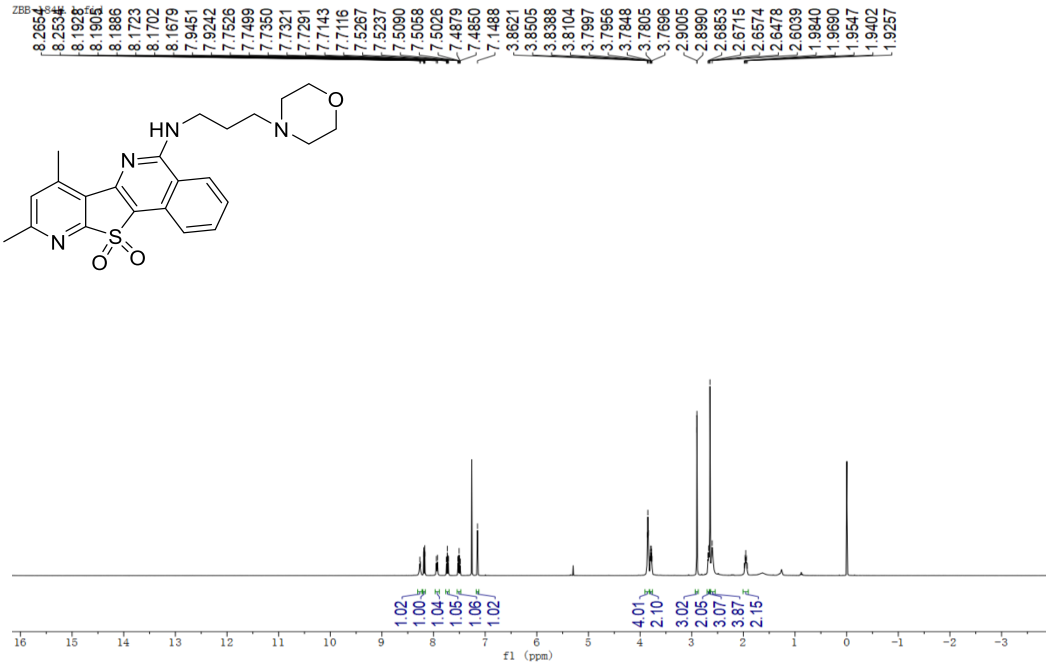

8.2654
8.2534
8.1928
8.1905
8.1886
8.1723
8.1702
8.1679
7.9451
7.9242
7.7526
7.7499
7.7350
7.7321
7.7291
7.7143
7.7116
7.5267
7.5237
7.5090
7.5058
7.5026
7.4879
7.4850
7.1488
3.8621
3.8505
3.8388
3.8104
3.7997
3.7956
3.7848
3.7805
3.7696
2.9005
2.8990
2.6853
2.6715
2.6574
2.6478
2.6039
1.9840
1.9690
1.9547
1.9402
1.9257
HN
N
O
N
N
S
O
O
1.02
1.00
1.04
1.05
1.06
1.02
4.01
2.10
3.02
2.05
3.07
3.87
2.15
f1 (ppm)

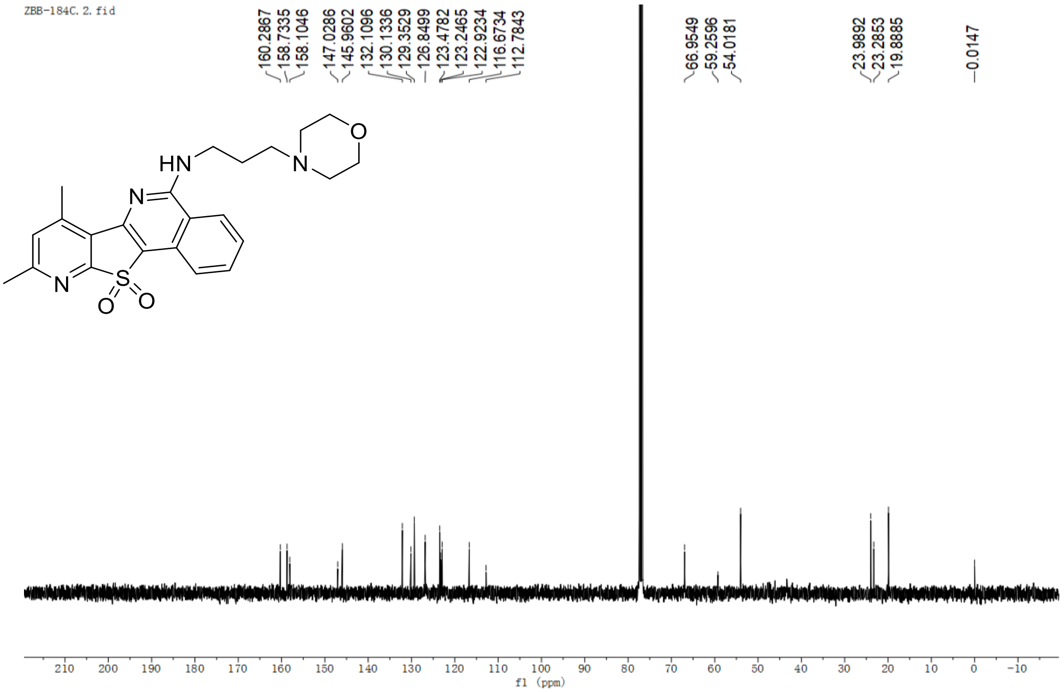

ZBB-184C.2.fid
160.2867
158.7335
158.1046
147.0286
145.9602
132.1096
130.1336
129.3529
126.8499
123.4782
123.2465
122.9234
116.6734
112.7843
66.9549
59.2596
54.0181
23.9892
23.2853
19.8885
-0.0147
HN
N
O
N
N
S
O
O
f1 (ppm)

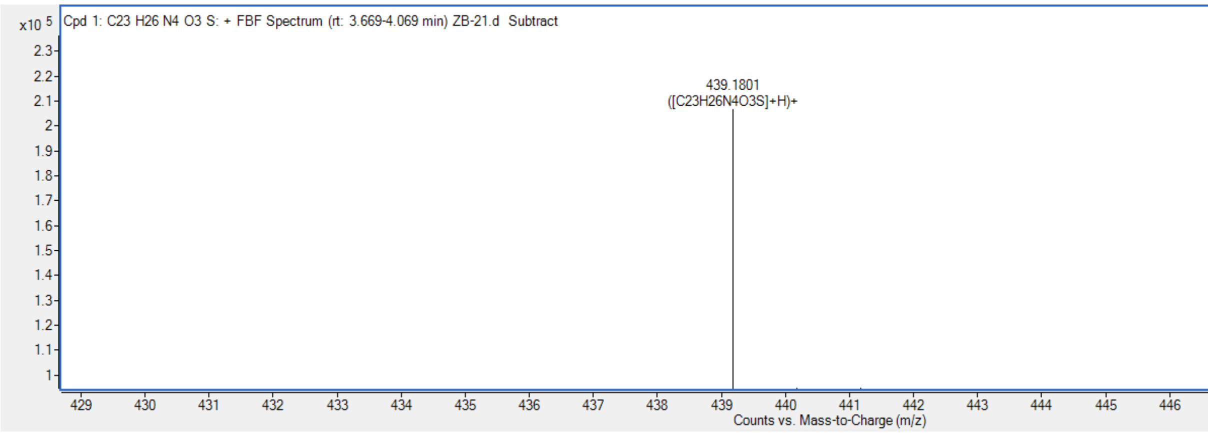

x10 5
Cpd 1: C23 H26 N4 O3 S: + FBF Spectrum (rt: 3.669-4.069 min) ZB-21.d Subtract
439.1801
([C23H26N4O3S]+H)+
Counts vs. Mass-to-Charge (m/z)

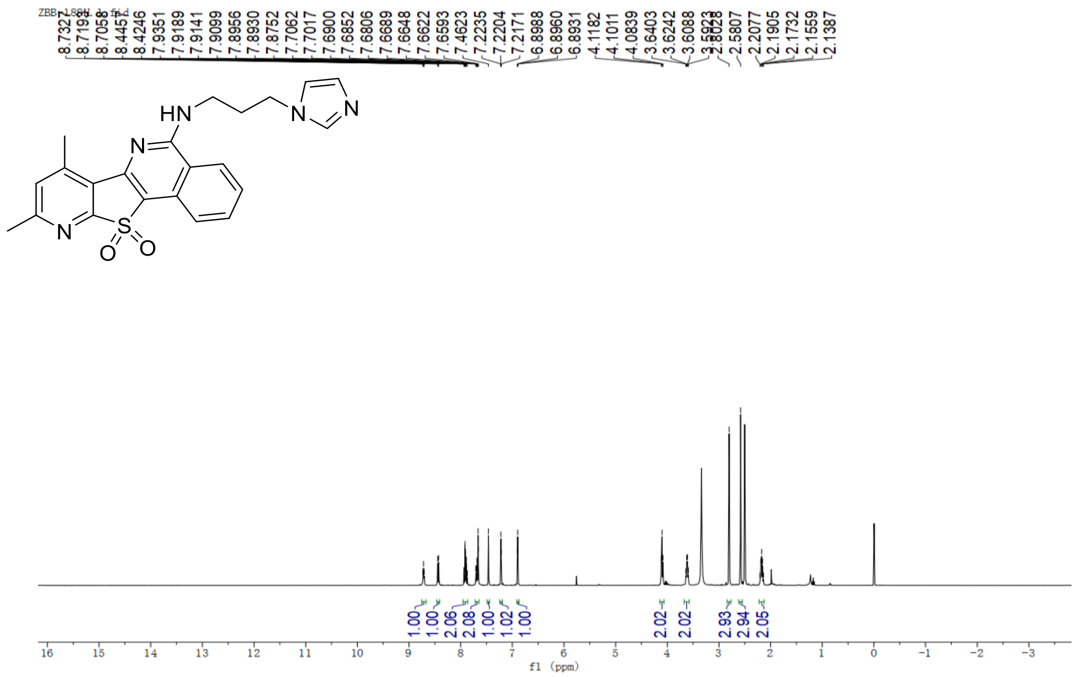
ZBB-188H.1.fid
8.7327 8.7193 8.7058 8.4457 8.4246 7.9351 7.9189 7.9141 7.9099 7.8956 7.8930 7.8752 7.7062 7.7017 7.6900 7.6852 7.6806 7.6689 7.6648 7.6622 7.6593 7.4623 7.2235 7.2204 7.2171 6.8988 6.8960 6.8931 4.1182 4.1011 4.0839 3.6403 3.6242 3.6088 2.5807 2.2077 2.1905 2.1732 2.1559 2.1387
HN
N
N
N
N
S
O
O
1.00 1.00 2.06 2.08 1.00 1.02 1.00 2.02 2.02 2.93 2.94 2.05
16 15 14 13 12 11 10 9 8 7 6 5 4 3 2 1 0 -1 -2 -3
f1 (ppm)

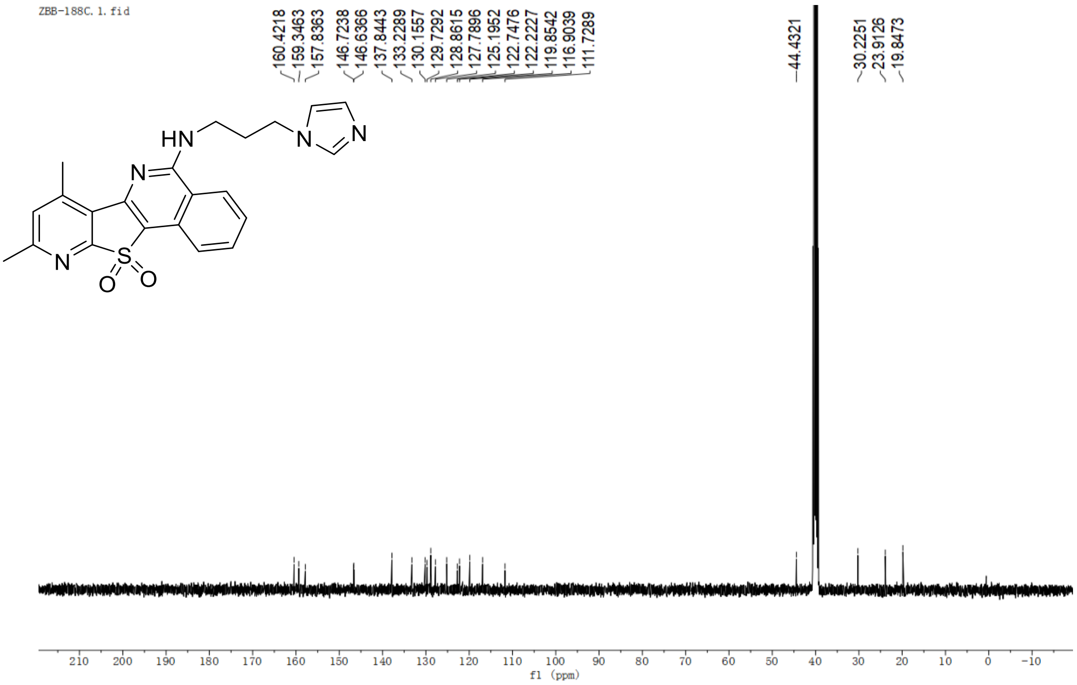
ZBB-188C.1.fid
160.4218 159.3463 157.8363 146.7238 146.6366 137.8443 133.2289 130.1557 129.7292 128.8615 127.7896 125.1952 122.7476 122.2227 119.8542 116.9039 111.7289
44.4321
30.2251 23.9126 19.8473
HN
N
N
N
N
S
O
O
210 200 190 180 170 160 150 140 130 120 110 100 90 80 70 60 50 40 30 20 10 0 -10
f1 (ppm)

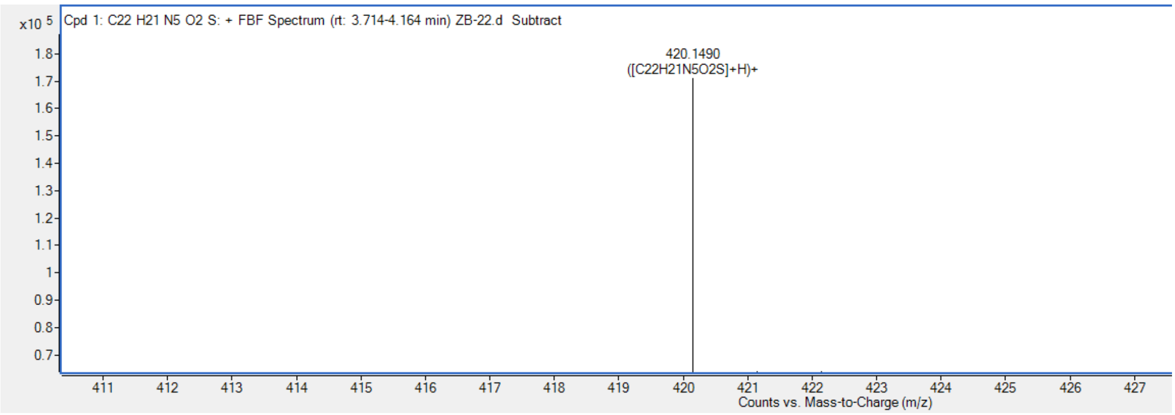
x10 5
Cpd 1: C22 H21 N5 O2 S: + FBF Spectrum (rt: 3.714-4.164 min) ZB-22.d Subtract
420.1490
([C22H21N5O2S]+H)+
1.8 1.7 1.6 1.5 1.4 1.3 1.2 1.1 1 0.9 0.8 0.7
411 412 413 414 415 416 417 418 419 420 421 422 423 424 425 426 427
Counts vs. Mass-to-Charge (m/z)

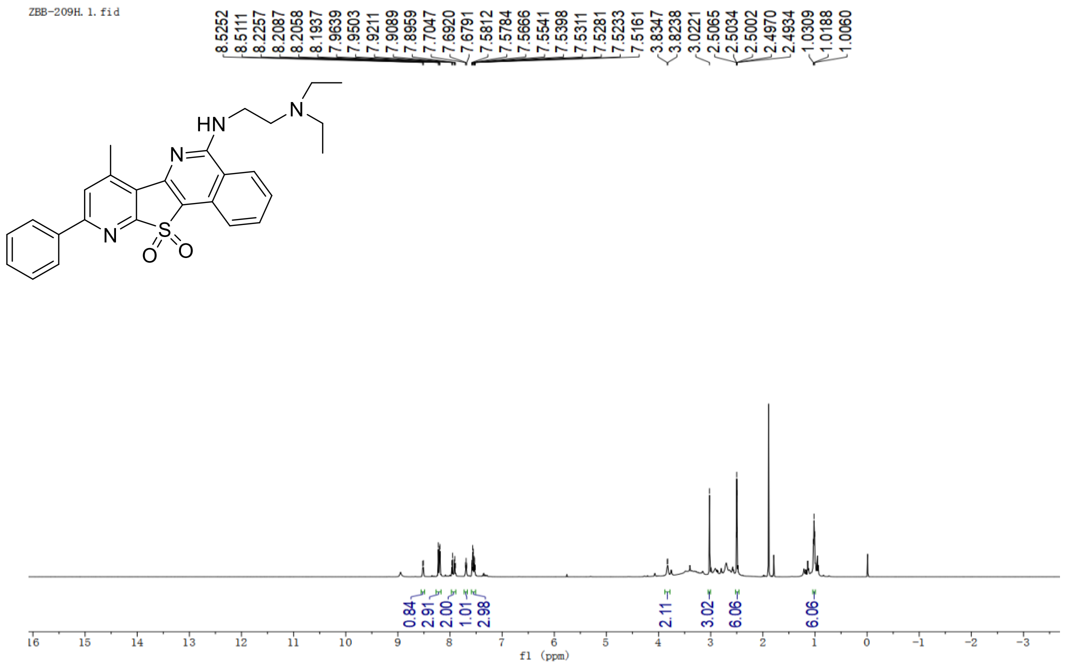
ZBB-209H.1.fid
8.5252
8.5111
8.2257
8.2087
8.2058
8.1937
7.9639
7.9503
7.9211
7.9089
7.8959
7.7047
7.6920
7.6791
7.5812
7.5784
7.5666
7.5541
7.5398
7.5311
7.5281
7.5233
7.5161
3.8347
3.8238
3.0221
2.5065
2.5034
2.5002
2.4970
2.4934
1.0309
1.0188
1.0060
HN
N
N
N
S
O
O
0.84
2.91
2.00
1.01
2.98
2.11
3.02
6.06
6.06
f1 (ppm)

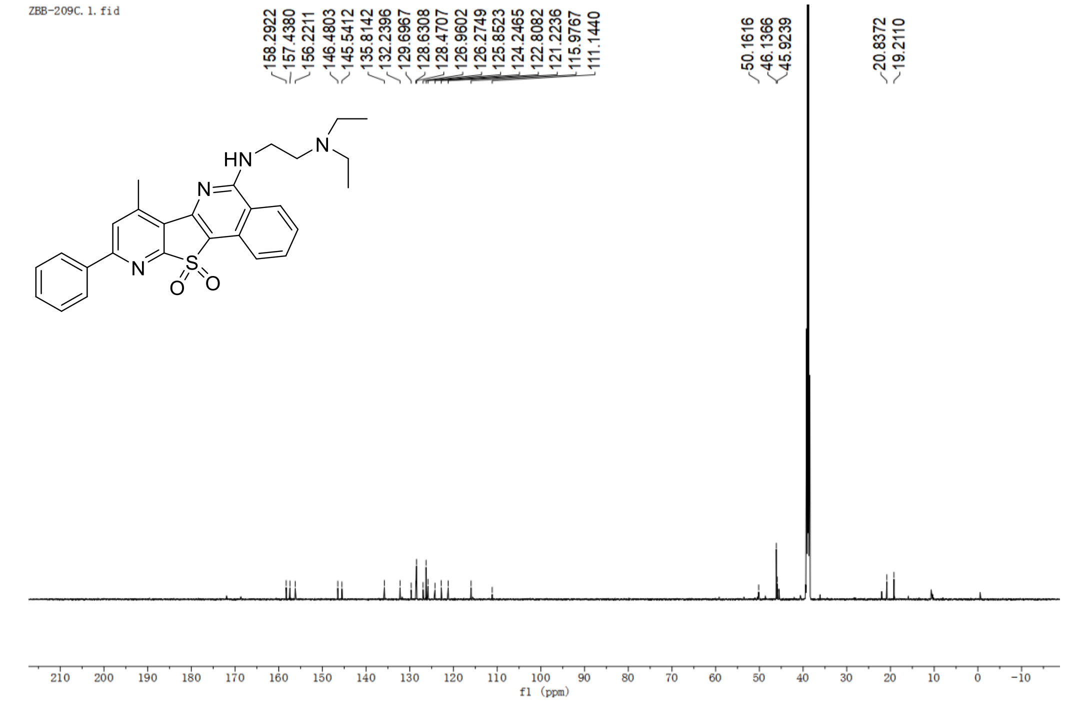
ZBB-209C.1.fid
158.2922
157.4380
156.2211
146.4803
145.5412
135.8142
132.2396
129.6967
128.6308
128.4707
126.9602
126.2749
125.8523
124.2465
122.8082
121.2236
115.9767
111.1440
50.1616
46.1366
45.9239
20.8372
19.2110
HN
N
N
N
S
O
O
f1 (ppm)

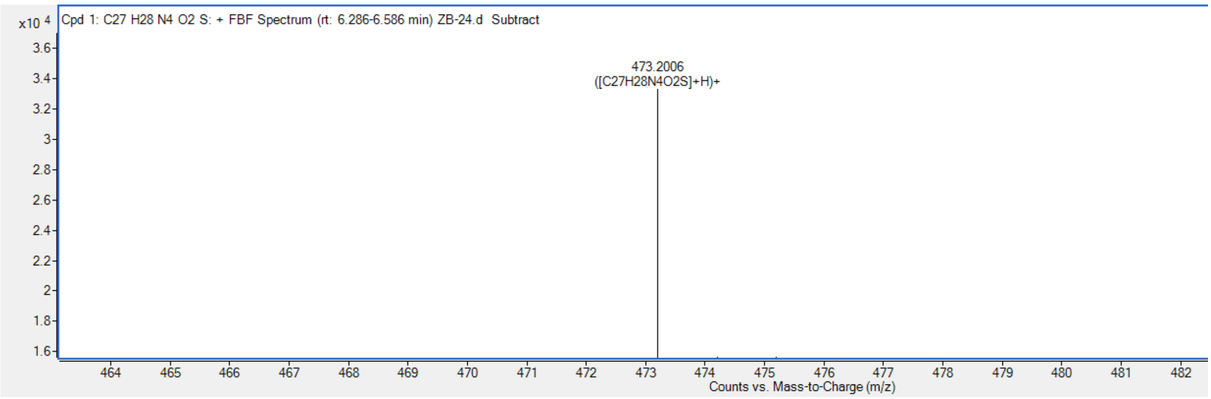
x10 4
Cpd 1: C27 H28 N4 O2 S: + FBF Spectrum (rt: 6.286-6.586 min) ZB-24.d Subtract
473.2006
([C27H28N4O2S]+H)+
Counts vs. Mass-to-Charge (m/z)

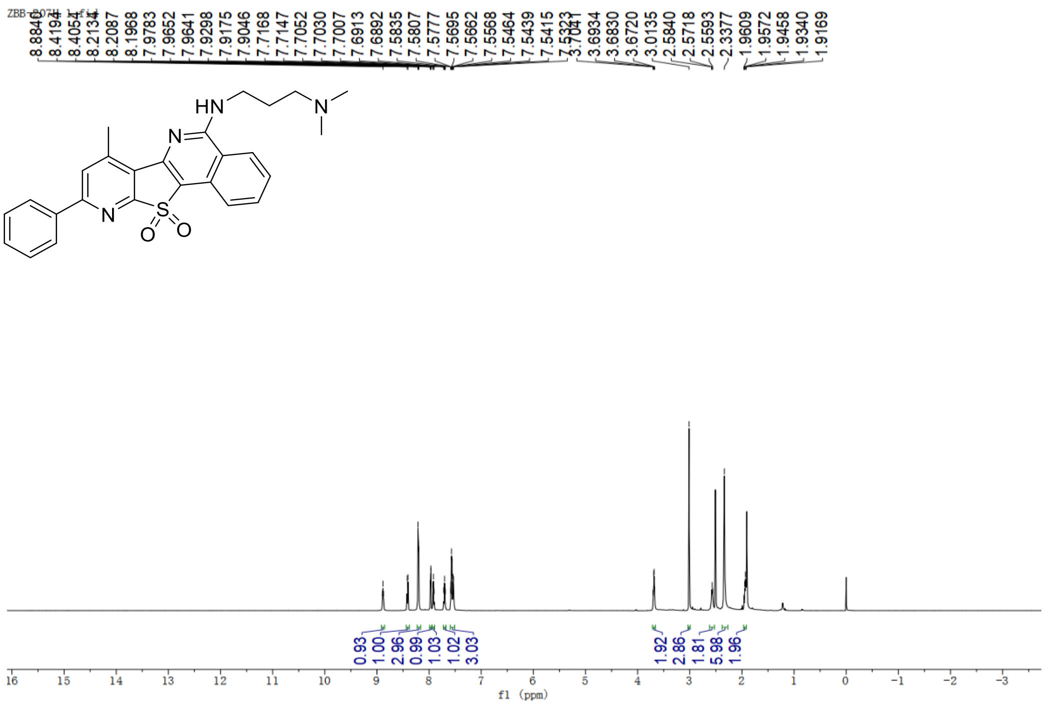
8.8840 8.4194 8.4054 8.2134 8.2087 8.1968 7.9783 7.9652 7.9641 7.9298 7.9175 7.9046 7.7168 7.7147 7.7052 7.7030 7.7007 7.6913 7.6892 7.5835 7.5807 7.5777 7.5695 7.5662 7.5568 7.5464 7.5439 7.5415 7.5323 3.7041 3.6934 3.6830 3.6720 3.0135 2.5840 2.5718 2.5593 2.3377 1.9609 1.9572 1.9458 1.9340 1.9169
HN
N
N
S
O
O
0.93 1.00 2.96 0.99 1.03 1.02 3.03 1.92 2.86 1.81 5.98 1.96
f1 (ppm)

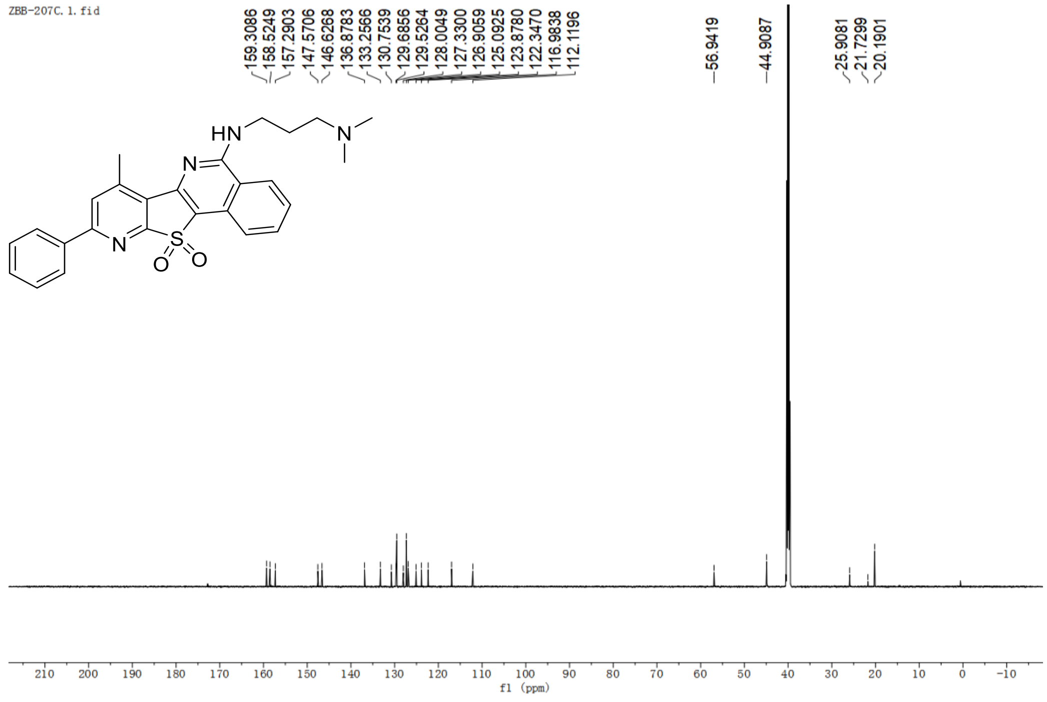
ZBB-207C.1.fid
159.3086 158.5249 157.2903 147.5706 146.6268 136.8783 133.2566 130.7539 129.6856 129.5264 128.0049 127.3300 126.9059 125.0925 123.8780 122.3470 116.9838 112.1196
56.9419
44.9087
25.9081 21.7299 20.1901
HN
N
N
S
O
O
f1 (ppm)

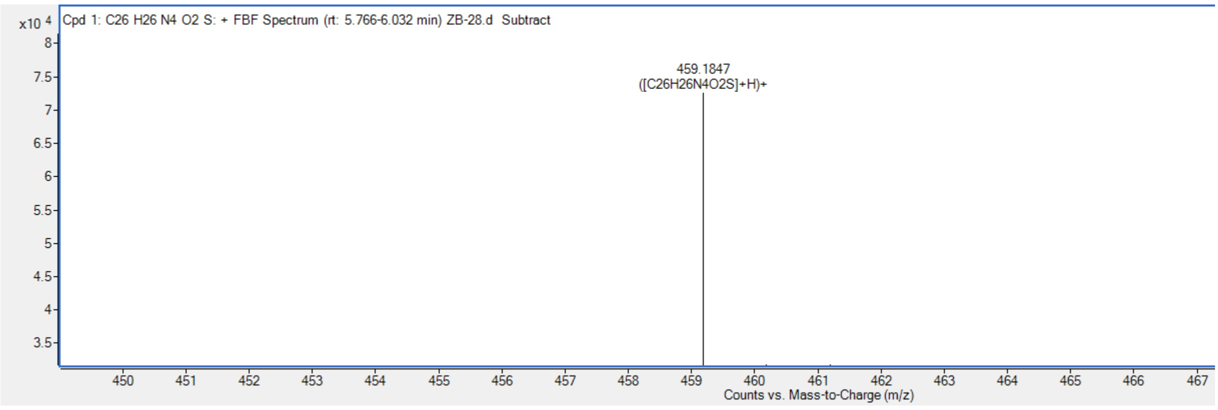
Cpd 1: C26 H26 N4 O2 S: + FBF Spectrum (rt: 5.766-6.032 min) ZB-28.d Subtract
x10 4
459.1847
([C26H26N4O2S]+H)+
Counts vs. Mass-to-Charge (m/z)

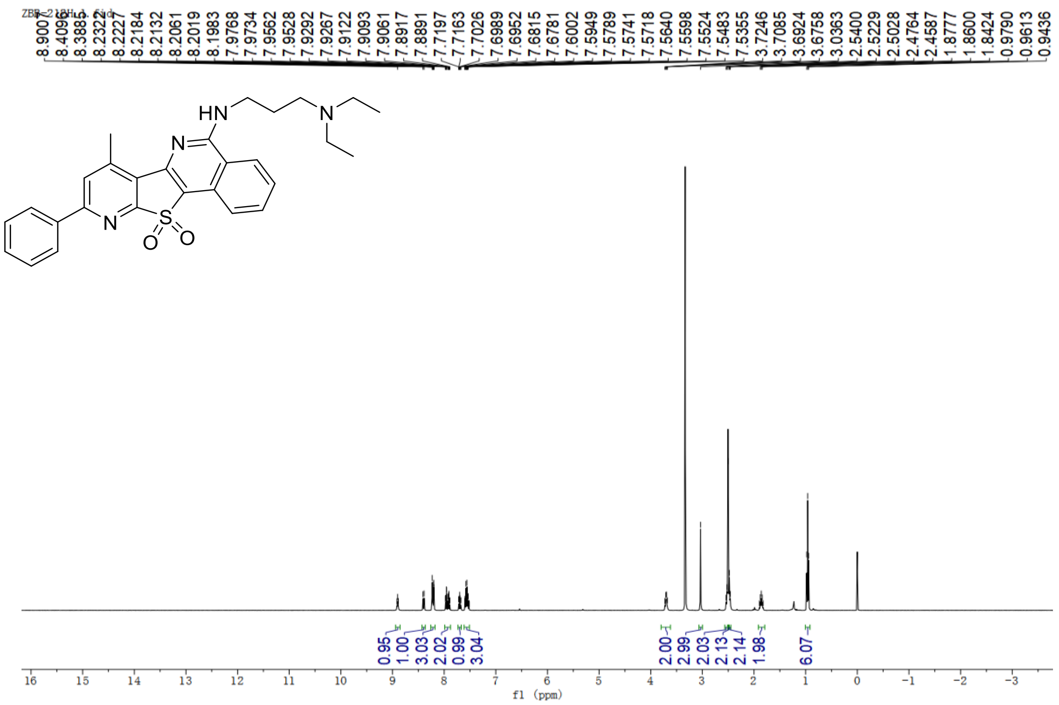
8.9007
8.4096
8.3883
8.2327
8.2227
8.2184
8.2132
8.2061
8.2019
8.1983
7.9768
7.9734
7.9562
7.9528
7.9292
7.9267
7.9122
7.9093
7.9061
7.8917
7.8891
7.7197
7.7163
7.7026
7.6989
7.6952
7.6815
7.6781
7.6002
7.5949
7.5789
7.5741
7.5718
7.5640
7.5598
7.5524
7.5483
7.5355
3.7246
3.7085
3.6924
3.6758
3.0363
2.5400
2.5229
2.5028
2.4764
2.4587
1.8777
1.8600
1.8424
0.9790
0.9613
0.9436
HN
N
N
N
S
O
O
0.95
1.00
3.03
2.02
0.99
3.04
2.00
2.99
2.03
2.13
2.14
1.98
6.07
f1 (ppm)

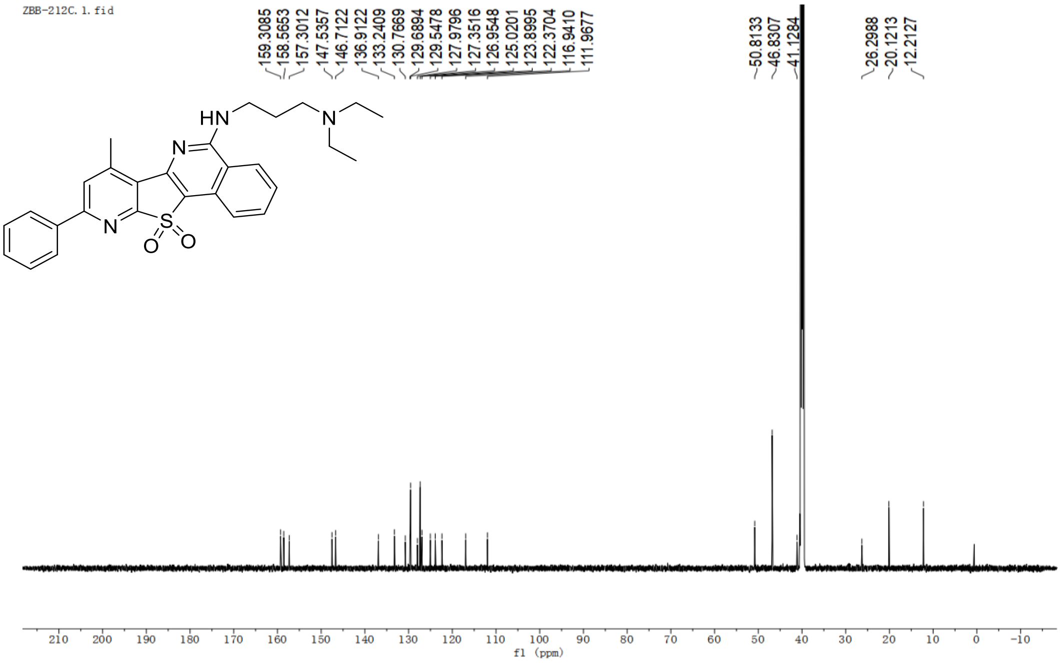
ZBB-212C.1.fid
159.3085
158.5653
157.3012
147.5357
146.7122
136.9122
133.2409
130.7669
129.6894
129.5478
127.9796
127.3516
126.9548
125.0201
123.8995
122.3704
116.9410
111.9677
50.8133
46.8307
41.1284
26.2988
20.1213
12.2127
HN
N
N
N
S
O
O
f1 (ppm)

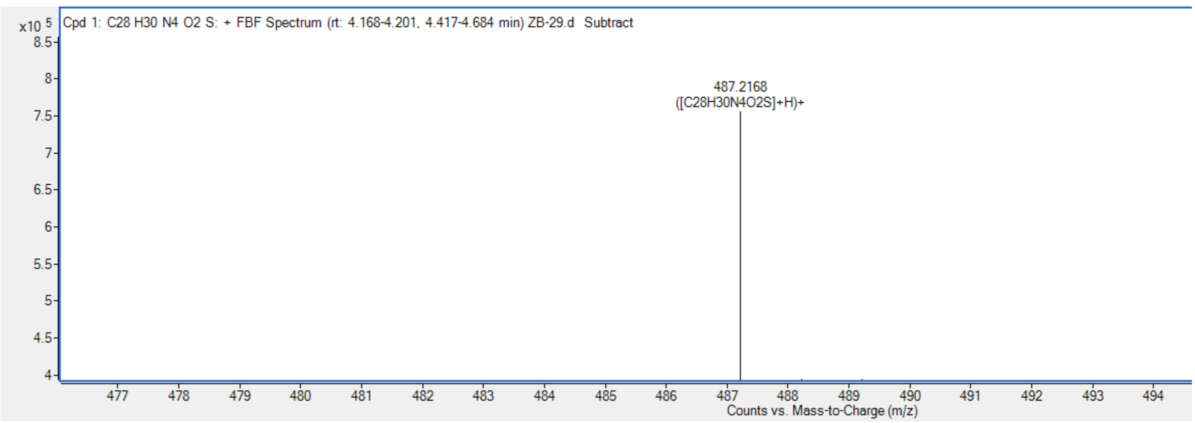
x10 5
Cpd 1: C28 H30 N4 O2 S: + FBF Spectrum (rt: 4.168-4.201, 4.417-4.684 min) ZB-29.d Subtract
487.2168
([C28H30N4O2S]+H)+
Counts vs. Mass-to-Charge (m/z)

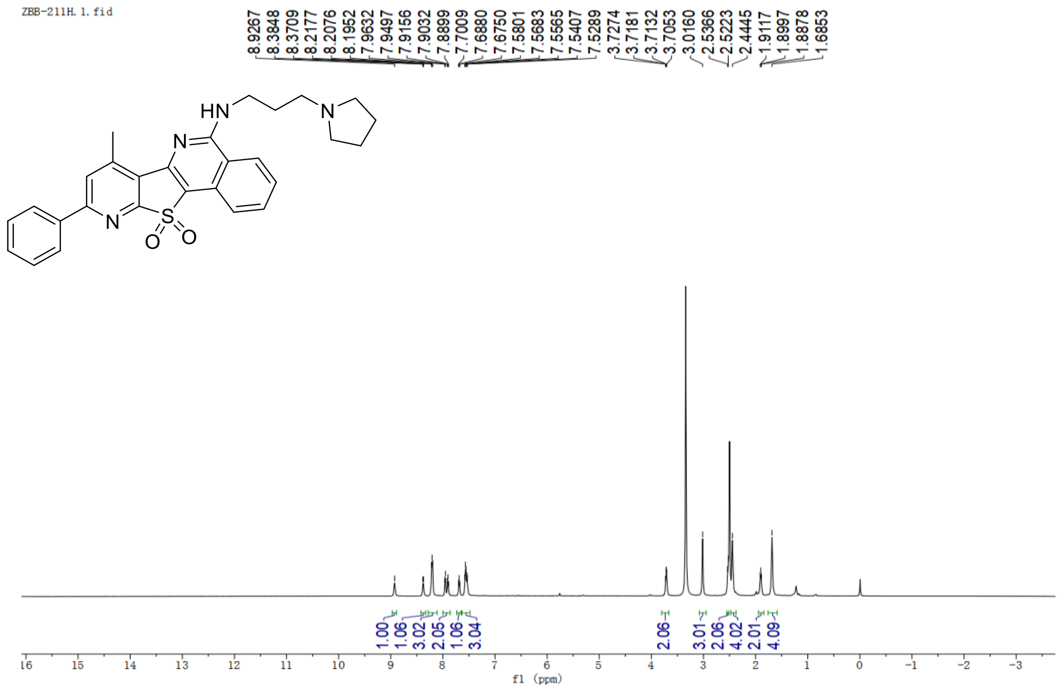
ZBB-211H. 1. fid
8.9267
8.3848
8.3709
8.2177
8.2076
8.1952
7.9632
7.9497
7.9156
7.9032
7.8899
7.7009
7.6880
7.6750
7.5801
7.5683
7.5565
7.5407
7.5289
3.7274
3.7181
3.7132
3.7053
3.0160
2.5366
2.5223
2.4445
1.9117
1.8997
1.8878
1.6853
HN
N
N
N
S
O
O
1.00
1.06
3.02
2.05
1.06
3.04
2.06
3.01
2.06
4.02
2.01
4.09
f1 (ppm)

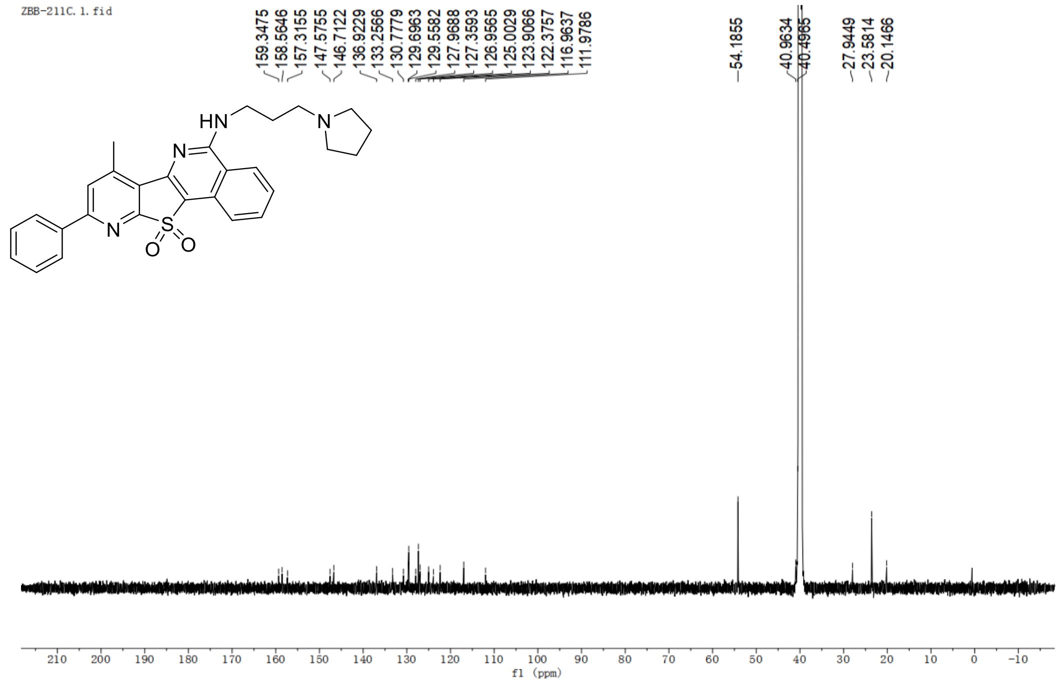
ZBB-211C. 1. fid
159.3475
158.5646
157.3155
147.5755
146.7122
136.9229
133.2566
130.7779
129.6963
129.5582
127.9688
127.3593
126.9565
125.0029
123.9066
122.3757
116.9637
111.9786
54.1855
40.9634
40.4965
27.9449
23.5814
20.1466
HN
N
N
N
S
O
O
f1 (ppm)

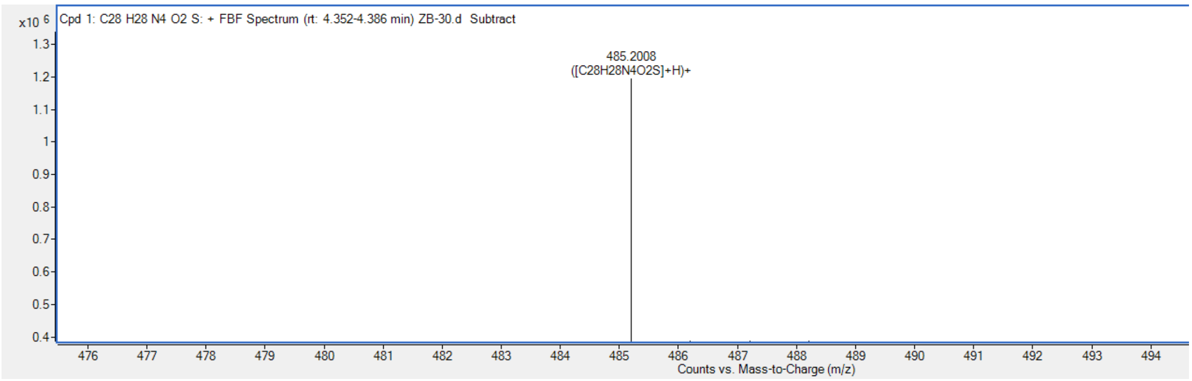
x10 6
Cpd 1: C28 H28 N4 O2 S: + FBF Spectrum (rt: 4.352-4.386 min) ZB-30.d Subtract
485.2008
([C28H28N4O2S]+H)+
Counts vs. Mass-to-Charge (m/z)

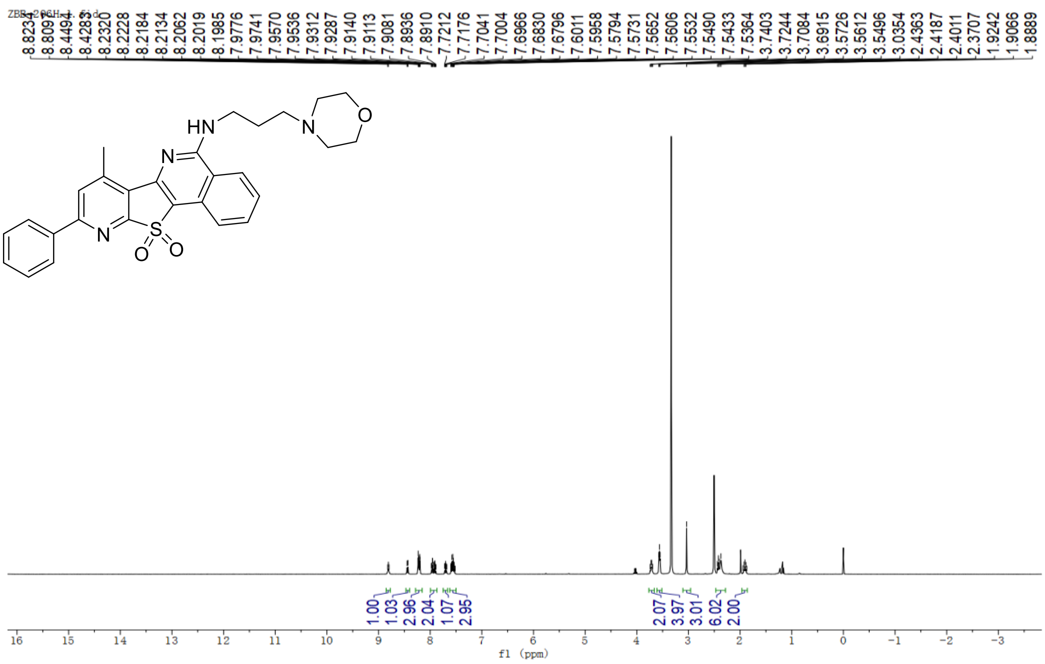
8.8231
8.8091
8.4494
8.4283
8.2320
8.2228
8.2184
8.2134
8.2062
8.2019
8.1985
7.9776
7.9741
7.9570
7.9536
7.9312
7.9287
7.9140
7.9113
7.9081
7.8936
7.8910
7.7212
7.7176
7.7041
7.7004
7.6966
7.6830
7.6796
7.6011
7.5958
7.5794
7.5731
7.5652
7.5606
7.5532
7.5490
7.5433
7.5364
3.7403
3.7244
3.7084
3.6915
3.5726
3.5612
3.5496
3.0354
2.4363
2.4187
2.4011
2.3707
1.9242
1.9066
1.8889
HN
N
O
N
N
S
O
O
1.00
1.03
2.96
2.04
1.07
2.95
2.07
3.97
3.01
6.02
2.00
f1 (ppm)

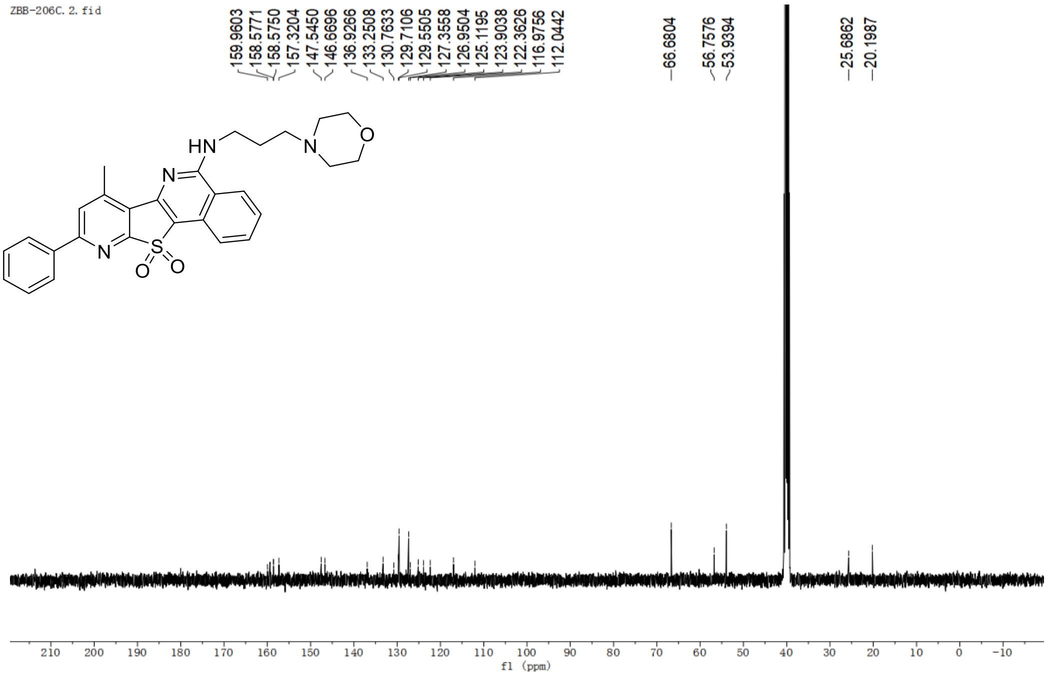
ZBB-206C.2.fid
159.9603
158.5771
158.5750
157.3204
147.5450
146.6696
136.9266
133.2508
130.7633
129.7106
129.5505
127.3558
126.9504
125.1195
123.9038
122.3626
116.9756
112.0442
66.6804
56.7576
53.9394
25.6862
20.1987
HN
N
O
N
N
S
O
O
f1 (ppm)

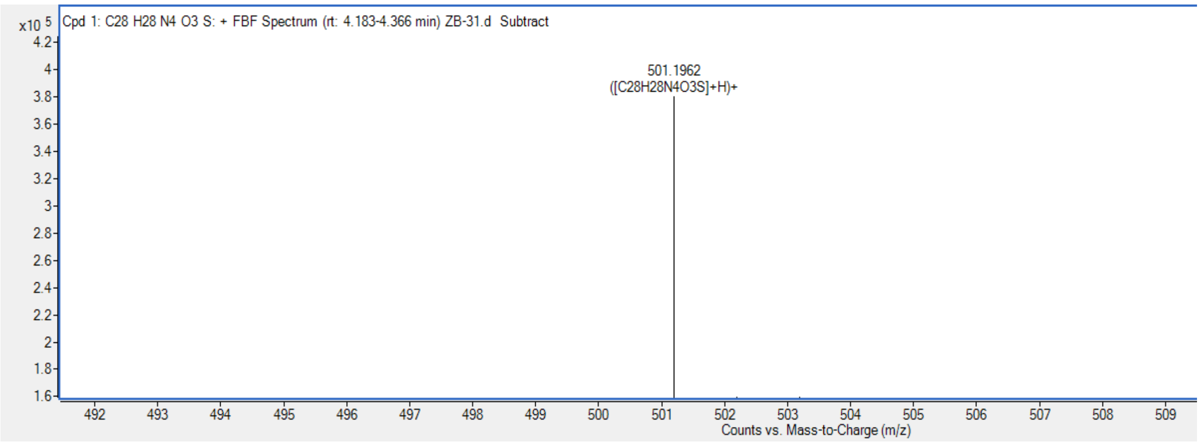
x10 5
Cpd 1: C28 H28 N4 O3 S: + FBF Spectrum (rt: 4.183-4.366 min) ZB-31.d Subtract
501.1962
([C28H28N4O3S]+H)+
Counts vs. Mass-to-Charge (m/z)
